# scAmp enables focal gene amplification analysis from single-cell data

**DOI:** 10.1038/s41467-026-76417-3

**Published:** 2026-08-08

**Authors:** Matthew G. Jones, Natasha E. Weiser, King L. Hung, Xiaowei Yan, Sangya Agarwal, Jens Luebeck, Aditi Gnanasekar, Shu Zhang, Ivy Tsz-Lo Wong, Jun Tang, Brooke E. Howitt, Ellis J. Curtis, Kevin Yu, John C. Rose, Katerina Kraft, Valeh Valiollah Pour Amiri, Leena Satpathy, Vineet Bafna, Paul S. Mischel, Howard Y. Chang

**Affiliations:** 1 Center for Personal Dynamic Regulomes, Stanford University, Stanford, CA USA; 2 Department of Pathology, Stanford University, Stanford, CA USA; 3 Sarafan ChEM-H, Stanford University, Stanford, CA USA; 4 Department of Computer Science and Engineering, University of California at San Diego, La Jolla, CA USA; 5 Department of Genetics, School of Medicine, Stanford University, Stanford, CA USA; 6 Medical Scientist Training Program, University of California San Diego, La Jolla, CA USA; 7 Koch Institute for Integrative Cancer Research, Massachusetts Institute of Technology, Cambridge, MA USA; 8 Arc Institute, Palo Alto, CA USA; 9 Present Address: Koch Institute for Integrative Cancer Research, Massachusetts Institute of Technology, Cambridge, MA USA; 10 Present Address: Institute for Medical Engineering and Science, Massachusetts Institute of Technology, Cambridge, MA USA; 11 Present Address: Department of Biology, Massachusetts Institute of Technology, Cambridge, MA USA; 12 Present Address: Department of Laboratory Medicine and Yale Cancer Center, Yale School of Medicine, New Haven, CT USA; 13 Present Address: Department of Neuroscience, Scripps Research, La Jolla, CA USA; 14 Present Address: Amgen Research, South San Francisco, CA USA; 15 Present Address: Department of Pediatrics, University of California, San Diego, La Jolla, CA USA

**Keywords:** Computational biology and bioinformatics, Cancer genetics, Genome-wide analysis of gene expression, Genetic mapping

## Abstract

Oncogene amplification on extrachromosomal DNA is a common driver of tumor progression and is associated with acquired drug resistance and poor patient survival. While bulk whole genome sequencing studies have revealed the landscape of genes amplified on extrachromosomal DNA in tumors, it remains challenging to study the subclonal heterogeneity and functional (e.g., transcriptomic) consequences of extrachromosomal DNA on tumors. To address this, we introduce *scAmp*: a probabilistic algorithm for detecting and analyzing extrachromosomal DNA from single-cell datasets. Using well-characterized cell lines, we demonstrate that *scAmp* has improved specificity over bulk genome sequencing in predicting extrachromosomal DNA status and can resolve the status of chromosomal amplifications that were historically extrachromosomal. We further showcase *scAmp* by analyzing 73 patient tumors profiled with single-cell assay for transposase-accessible chromatin by sequencing, where we characterize the subclonal evolution of subclones with extrachromosomal DNA and identify the effect of these amplifications on the chromatin accessibility landscape of cancer cells. Finally, we provide proof-of-concept analyses that *scAmp* aids in the detection of extrachromosomal DNA from clinical histopathology assays. Together, we anticipate that *scAmp* will broadly enable further studies – both retrospective and prospective – that dissect critical questions of how extrachromosomal DNAs affect cancer cells and the tumors in which they reside.

## Abstract (web summary)

Oncogene amplification on extrachromosomal DNA (ecDNA) is associated with poor patient prognosis and drug resistance but can be difficult to detect. Here, the authors describe a new computational tool, single-cell amplicon (“*scAmp*”), which enables the study of ecDNA from single-cell assays.

## Introduction

Focal amplification of DNA is a prevalent driver of tumor evolution^1,2^, but the mode of amplification can profoundly alter progression^3^. Circular extrachromosomal DNA (ecDNA, or “double minutes”) amplifications are found in approximately 17% of primary tumors overall and are associated with significantly worse patient outcomes and increased drug resistance as compared to chromosomal amplifications (including linear amplifications and complex non-cyclic amplifications like those formed via breakage-fusion-bridge [BFB] processes)^3–5^. While whole-genome sequencing (WGS) analyses of large patient cohorts have revealed the landscape of genes amplified on ecDNA^3,4,6–8^ (including canonical oncogenes like *MYC*, regulatory elements, and immunomodulatory genes like *SOCS1*), several questions remain that require increased resolution and molecular characterization of ecDNA+ cells: for example, what is the distribution of ecDNAs among cancer cells within a tumor? How are ecDNAs associated with differential cancer cell programs and immune or stromal populations?

**Fig. 1:**
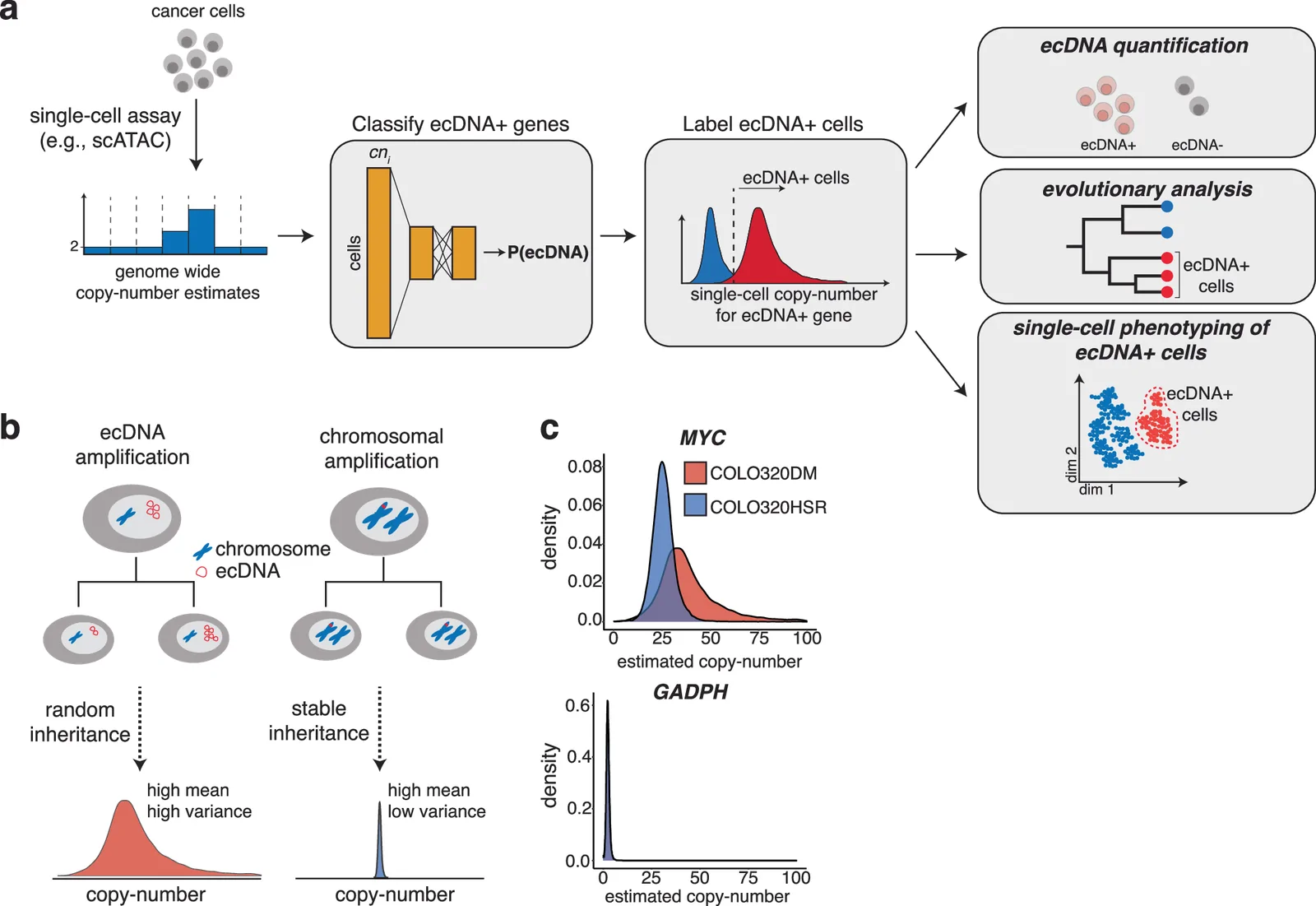
*scAmp* detects ecDNA-amplified genes from single-cell copy-number data. **a** An overview of the *scAmp* workflow for predicting extrachromosomal DNA (ecDNA) status. Copy numbers (*cn)* are inferred from individual cells (e.g., from single-cell ATAC-seq [scATAC-seq] data). For each gene, *scAmp* then infers the likelihood of that gene being amplified on extrachromosomal DNA (*P(ecDNA)*) based on the properties of its copy-number distribution across cells. Then, cells are labeled as ecDNA+ or ecDNA- based on a heuristic thresholding scheme. This enables downstream analyses such as quantifying the prevalence of ecDNA in a tumor, assessing an ecDNA’s evolutionary history, and investigating the phenotypic effects of ecDNA on cancer cell state. **b** A schematic representation of how random ecDNA segregation leads to greater copy-number variance. **c** Evaluation of single-cell copy-number distributions of *MYC* in COLO320DM (ecDNA+) and COLO320HSR (non-ecDNA), as compared to *GAPDH*.

Current approaches for ecDNA detection focus on specific sequence features defined by bulk WGS^8–10^. However, these bulk WGS-based approaches can have difficulty discriminating amplicons that persist as ecDNAs from those that have integrated back into chromosomes, often because the integrated amplicons retain the rearranged sequences and circular features derived from their ancestral ecDNAs. To overcome this limitation, we hypothesized that a defining feature of ecDNA biology – the random inheritance of individual ecDNA molecules during cell division^11^ – could enable an unbiased discovery of ecDNA from single-cell assays and address fundamental questions of how ecDNAs affect tumor biology^12–14^ (Fig. 1).

In this work, we leverage the discriminative ability of single-cell copy-number distributions to enable single-cell analysis of ecDNA with a computational framework called *scAmp* (“single-cell amplicon” analysis; Fig. 1a). Specifically, due to their lack of centromeres, ecDNAs are randomly inherited during mitosis and thus drive greater copy-number variance in a population compared to chromosomal amplifications^11,15^ (Fig. 1b). We use this principle to build *scAmp* and demonstrate that it has higher specificity than bulk sequencing approaches alone, leading to the discovery of a class of chromosomal amplifications that were likely historically extrachromosomal. We then employ *scAmp* in retrospective analyses of single-cell patient data, illustrating its ability to quantify ecDNA and dissect the subclonal architecture of ecDNA tumors.

## Results

### Developing an ecDNA classifier from single-cell copy-numbers

*scAmp* operates on single-cell copy-number data (which can be obtained from assays such as single-cell WGS [scWGS] ^16–18^ or single-cell assay for transposase-accessible chromatin by sequencing [scATAC-seq]^17^) and predicts a likelihood for each gene that it is amplified on ecDNA. An analysis of the isogenic cell line pair – COLO320DM (containing ecDNA-amplified *MYC*) and COLO320HSR (containing chromosomally-amplified *MYC*)—underscores the discriminative properties of single-cell copy-number distributions: while *MYC* is amplified in both cell lines, the variance is substantially greater in COLO320DM cells (Fig. 1c and Supplementary Fig. 1a; “Methods”). This principle is similar to algorithms for predicting ecDNA-amplified regions from allelic imbalance in single-cell gene expression data^19^, though the focus on copy-number distributions extends across many platforms. From these predicted ecDNA amplifications, cells can be labeled as ecDNA+ based on their copy numbers (for example, a threshold based on copy-number heuristics).

**Fig. 2:**
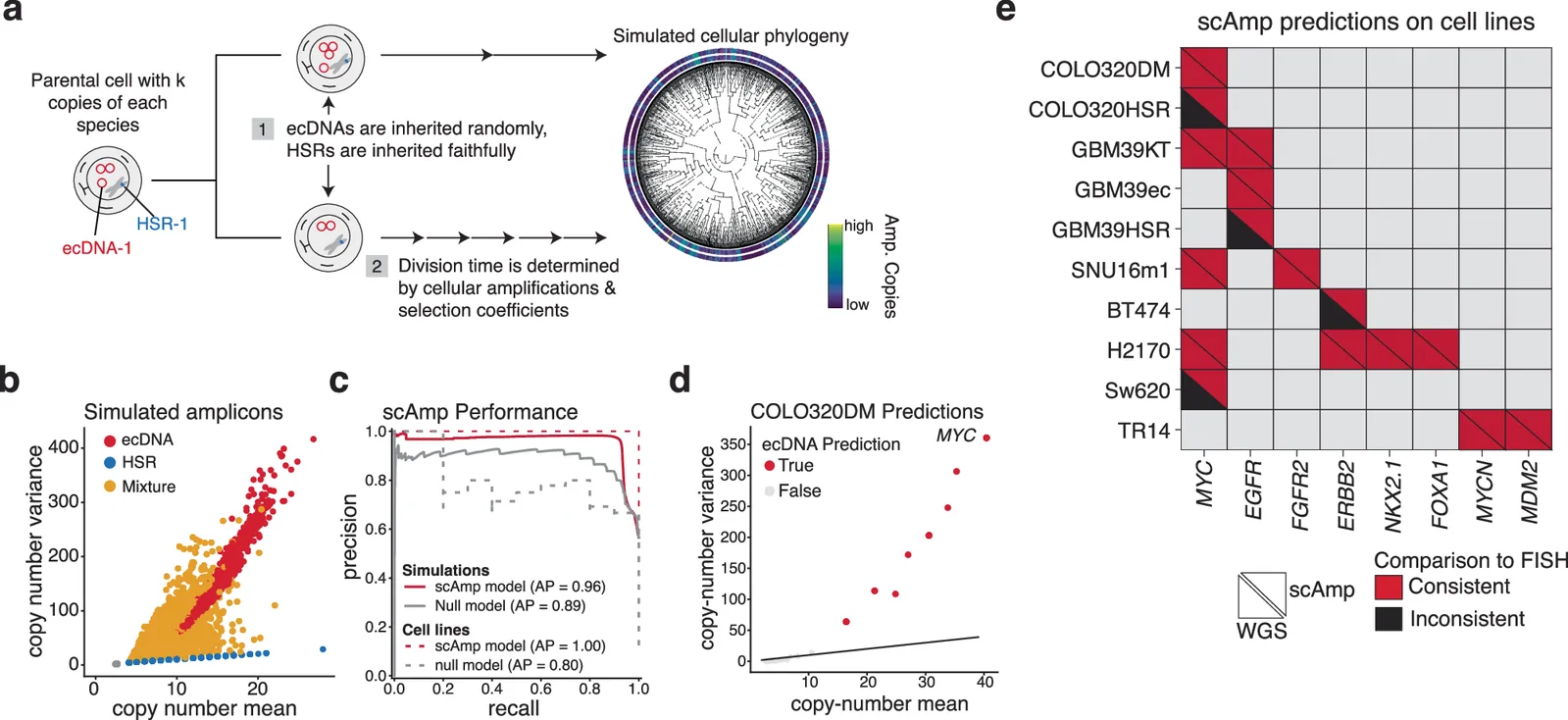
Training and benchmarking *scAmp* on well-characterized cell-line data. **a** A schematic representation of the simulation framework for generating training data for machine learning models. **b** Overview of simulated amplicon distributions from the simulation framework displayed in (**a**). **c** ROC curves showing the performance of *scAmp* versus a “null” model based on mean copy-number alone on training data. **d** Summary of ecDNA predictions and copy-number distributions in the COLO320DM cell line. Each point indicates an individual gene, and genes validated by DNA fluorescence in situ hybridization (FISH) are annotated. **e** Summary of gene-level predictions from *scAmp* and bulk whole genome sequencing (WGS) across cell lines. Agreement between an assay and DNA FISH on metaphase spread ground-truth is marked in red; disagreement is marked in black. Source data are provided as a Source Data file.

We trained gene-level classifiers from simulated copy-number distributions of ecDNA and chromosomal amplifications using a previously-described forward-time evolutionary model of focal amplifications^11,14^ (Fig. 2a; “Methods”). After simulating copy-number trajectories, we created a range of synthetic single-cell datasets, including those with mixed ecDNA and chromosomal amplifications (Fig. 2b; “Methods”).

We first evaluated model performance on a scATAC-seq dataset consisting of 10 well-characterized cell lines profiled with fluorescence in situ hybridization (FISH) of DNA on metaphase spreads, the gold-standard for assessing amplification status (including COLO320DM and HSR^5,20^; GBM39ec, HSR, and KT^5,14,21^; SNU16m1^14,22,23^; TR14^14,22^; and H2170^15,24,25^; Supplementary Fig. 1b, c). We estimated single-cell copy numbers from the scATAC-seq data for the FISH-validated oncogene amplifications and found that our simulated data closely recapitulated the dispersion of observed cell line distributions (Supplementary Fig. 2a; “Methods”). After training several models on simulated data and testing on these validated cell lines, we found a set of best-performing models that had high AUPRCs on simulated data and perfect agreement with cell line characterization (Fig. 2c; “Methods”).

To refine our model selection, we assessed the concordance of predictions from single-cell and bulk WGS assays in tumor samples from The Cancer Genome Atlas^6,26^ (Supplementary Fig. 2b–e and Supplementary Fig. 3). From bulk WGS-based analysis, these tumors contained a variety of amplifications (including ecDNA and complex non-cyclic amplifications; Supplementary Fig. 3a, b), and copy-numbers estimates were broadly consistent between scATAC-seq and bulk WGS (Supplementary Fig. 3c; “Methods”). From these analyses, we determined a best-performing multi-layer perceptron (MLP) with an AUPRC of 0.96 on simulated data (as compared to an AUPRC of 0.89 for a logistic regression null model trained just on mean copy-numbers; Fig. 2c), perfect agreement with previous characterization of cell lines as opposed to baseline models (an AUPRC of 1.0 for *scAmp* versus 0.80 for the null model; Fig. 2c–e and Supplementary Fig. 4; “Methods”), and high concordance with bulk WGS-based predictions of patient tumor samples (AUROC of 0.86; Supplementary Fig. 2e and Supplementary Fig. 5).

We further characterized our best-performing model with additional benchmarks that reflect realistic tumor settings. We first found that *scAmp* performed well at predicting oncogene amplifications in cell lines (with a perfect AUROC) across a variety of pre-processing parameter settings (Supplementary Fig. 6a; “Methods”). To test the model’s performance when a gene is amplified on both ecDNA and chromosomes, we next performed in silico mixture experiments with the COLO320DM/HSR and GBM39ec/HSR isogenic pairs (whose oncogene amplification status was verified with DNA FISH on metaphase spreads; Supplementary Fig. 1c; “Methods”). We found that our model was robust to a range of ecDNA purities, remaining highly accurate if at least 15–20% of cells contained ecDNA (Supplementary Fig. 6b). Moreover, unlike the null model, we found that our best-performing model remained accurate at classifying genes as ecDNA+ across a wide range of copy-number regimes (including when the ecDNA+ gene was at low copy-numbers; Supplementary Fig. 6c, d) and levels of noise (Supplementary Fig. 6e).

### scAmp resolves amplification mode ambiguities in bulk assays

Cases in which *scAmp* disagreed with bulk WGS revealed notable limitations of bulk genome sequencing for determining ecDNA status (Fig. 2e). For example, the breast cancer cell line BT474 was predicted by bulk WGS to have an ecDNA containing *ERBB2*^6^, while *scAmp* predicted *ERBB2* to be chromosomally amplified (Fig. 2e and Supplementary Fig. 7a, b). Definitive characterization of *ERBB2’*s amplification mode by DNA FISH on metaphase spreads of BT474 cells revealed that, indeed, *ERBB2* was chromosomally amplified, consistent with the prediction from *scAmp* (Supplementary Fig. 7c, “Methods”). Similarly, in the colorectal cancer cell line Sw620, we found that *scAmp* correctly identified *MYC* as chromosomally amplified (as previously verified with DNA FISH on metaphase spreads^5^; Supplementary Fig. 7d–f), whereas bulk WGS-based profiling predicted this gene to be ecDNA-amplified^6^ (Fig. 2e).

The failure of bulk WGS to correctly determine these gene amplification modes likely derived from either: (1) the ambiguity of head-to-tail reads that could resemble ecDNA or other amplifications like fold-back inversions (FBIs)^15^; or (2) the historical presence of ecDNA that has since integrated into the chromosome, retaining the discordant read structure that would support an ecDNA classification in bulk WGS analysis^6,14,21,27^. Indeed, the difficulty of bulk WGS-based assays in predicting amplification status of integrated ecDNA is also observed in the case of COLO320HSR and GBM39HSR - isogenic variants of ecDNA+ cell lines in which the ecDNA-amplified gene has integrated into the chromosome (Supplementary Fig. 1 and Fig. 2e). These results demonstrate that there exists a class of historical ecDNA amplifications that single-cell characterization with *scAmp* can resolve with higher specificity than bulk sequencing approaches alone.

### scAmp enables retrospective analysis of tumors profiled with single-cell assays

**Fig. 3:**
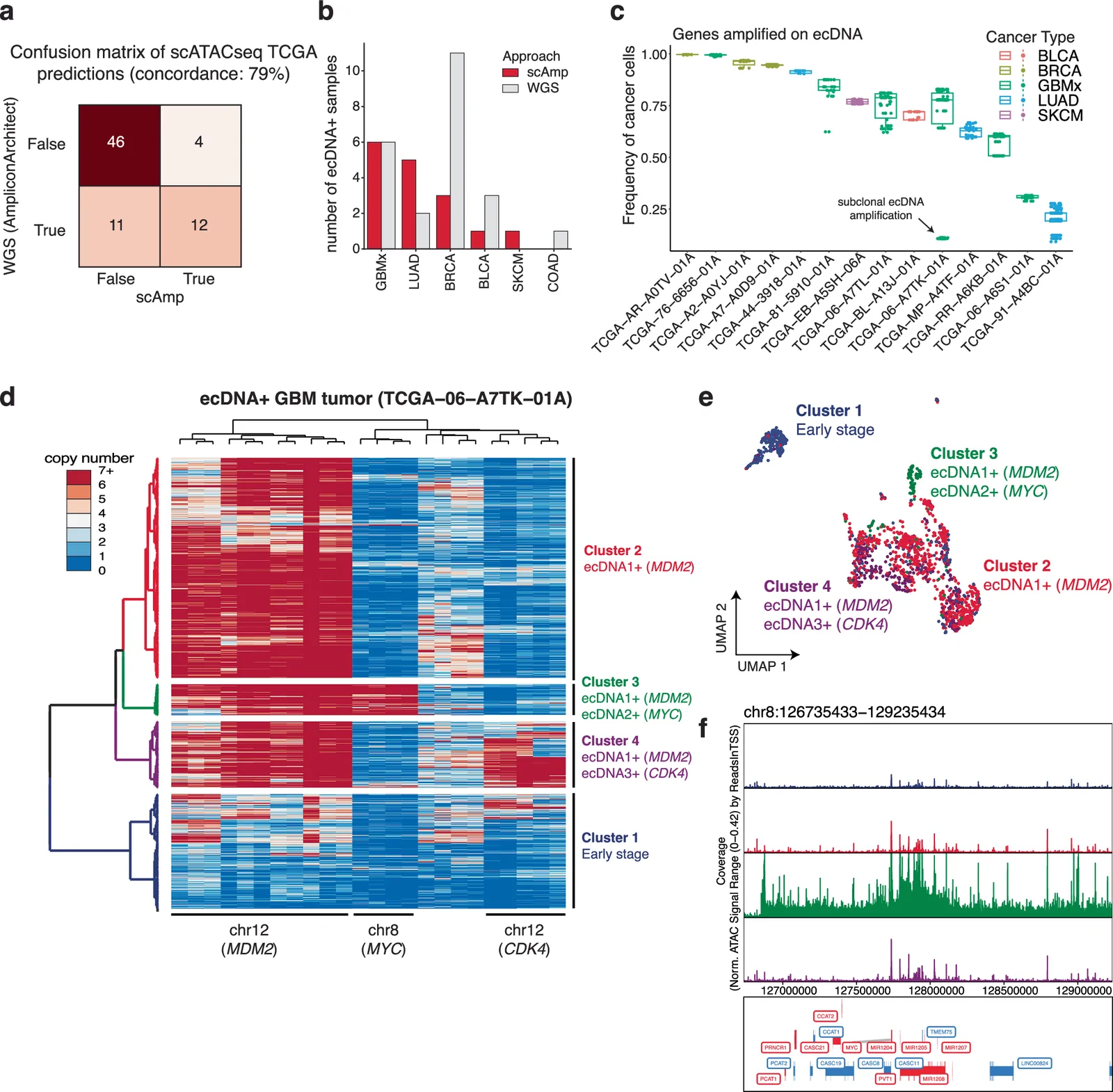
*scAmp* enables retrospective analysis and classification of patient tumors. **a** Confusion matrix reporting the concordance between *scAmp* and whole genome sequencing (WGS) predictions on a cohort of 73 patient tumors from The Cancer Genome Atlas (TCGA) profiled with single-cell assay for transposase-accessible chromatin by sequencing (scATAC-seq). **b** Overview of extrachromosomal DNA (ecDNA) classifications in each tumor type, comparing *scAmp* and WGS calls. **c** Fraction of cancer cells with ecDNA amplifications across all ecDNA+ tumor samples. Each point is a gene predicted to be on ecDNA, and each boxplot corresponds to a single tumor. Boxplots report the quartiles of the distributions (the center corresponding to the median and box edges corresponding to the 25^th^ and 75^th^ percentiles), and whiskers extend to 1.5x the interquartile distance. **d** Clustered heatmap of ecDNA-amplified 3 Mb windows in an example glioblastoma (GBM) tumor sample, TCGA−06−A7TK−01A. Four hierarchical clusters are identified based on tree depth and annotated according to the representative oncogenes found on ecDNA amplifications (“Methods”). Chromosomes and representative focal genes are indicated below the heatmap. **e** Uniform Manifold Approximation and Projection (UMAP) of single-cell chromatin accessibility data, with colors corresponding to the hierarchical clusters presented in (**d**). **f** Chromatin accessibility tracks for a chromosome 8 region centered on *MYC*. Tracks are pseudo-bulked and colored according to the hierarchical clusters identified in (**d**). BLCA = Urothelial Bladder Carcinoma; BRCA = Breast Invasive Carcinoma; GBMx = Glioblastoma Multiforme; LUAD = Lung Adenocarcinoma; SKCM = Skin Cutaneous Melanoma. Source data are provided as a Source Data file.

With *scAmp*’s ability to detect ecDNA status of genes from single-cell data, we next turned to assessing its utility on patient samples and re-analyzed a recent cohort of 73 patient tumors profiled with scATAC-seq through The Cancer Genome Atlas^26^ (Fig. 3a; “Methods”). As reported above, we found that *scAmp*’s predictions were largely concordant with bulk WGS data and improved over Random Forest predictions (80% agreement vs 70% on a per-gene level, and 79% vs 32% that a tumor contained at least one ecDNA-amplified gene**;** Supplementary Fig. 5; and Supplementary Fig. 8a). In this dataset, gliomas (GBMx), lung cancers (LUAD), and breast cancers (BRCA) had the highest prevalence of ecDNA (Fig. 3b). Moreover, scAmp predicts key genes to be commonly amplified on ecDNA, including *EGFR*, *MYC*, and *KRAS* (Supplementary Fig. 8b). Similar to the analysis of BT474 and Sw620 cell lines, in the cases that *scAmp* disagrees with bulk WGS, we find that the copy-number distribution supports *scAmp*’s predictions (Supplementary Fig. 8c, d).

Applying *scAmp* to assays like scATAC-seq enables the phenotypic analysis of cell states within ecDNA+ tumors (Supplementary Fig. 9). Stratifying by tumor type, we find substantial shifts in the immune composition of ecDNA+ tumors: for example, T cells are significantly enriched in BRCA, LUAD, and SKCM ecDNA+ tumors, whereas macrophages were specifically increased in ecDNA+ GBM samples (Supplementary Fig. 9a, b**)**. We further leveraged the single-cell nature of this assay to stratify cancer cells within the same tumor by ecDNA status and inspect cell state changes (Supplementary Fig. 9c). We found consistent changes in ecDNA+ cancer cells, including up-regulation of glycolysis (*p* = 0.02, one-sided Wilcoxon rank-sums test) and hypoxia-sensing pathways (*p* = 0.06, one-sided Wilcoxon rank-sums test) as well as down-regulation of mitotic spindle assembly and reactive oxygen species signatures (*p* = 0.12 and *p* = 0.1, respectively, one-sided Wilcoxon rank-sums test).

Recent literature has highlighted the functional importance of ecDNA in driving distinct stages of tumor progression^28,29^, and the ability to detect ecDNA on a cell-by-cell basis provides the opportunity to infer the subclonal evolution of ecDNA within tumors^12^. Focusing on just cancer cells, we find a striking variability in the fraction of ecDNA-amplified cells across tumors: while ecDNA appears in almost every cancer cell in some tumors, in others, ecDNA amplifications may only be found in ~10% of cancer cells (Fig. 3c and Supplementary Fig. 9d). The distribution of ecDNA within a single tumor may provide insight into the relative evolutionary timing of ecDNA biogenesis. For example, in one GBM tumor (TCGA−06−A7TK−01A) we find that while ~80% of cancer cells contain an ecDNA amplifying *MDM2*, two distinct subsets of cancer cells also contain an additional ecDNA: one encoding *MYC* (Cluster 3) and another encoding *CDK4* (Cluster 4) (Fig. 3d). These data suggest that the additional ecDNAs may have originated in a subclone of the larger ecDNA+ population, either in response to ongoing genomic instability or selection pressure imparted from the tumor microenvironment. We also find that regulatory changes in chromatin accessibility accompany these changes in ecDNA content of subpopulations: cancer cells with the subclonal *MYC* ecDNA cluster separately and exhibit highly accessible chromatin of the amplified chromosome 8 locus as compared to the cells that do not amplify this *MYC* ecDNA, consistent with the previous finding that ecDNA chromatin is highly accessible^30^ (Fig. 3e, f).

**Fig. 4:**
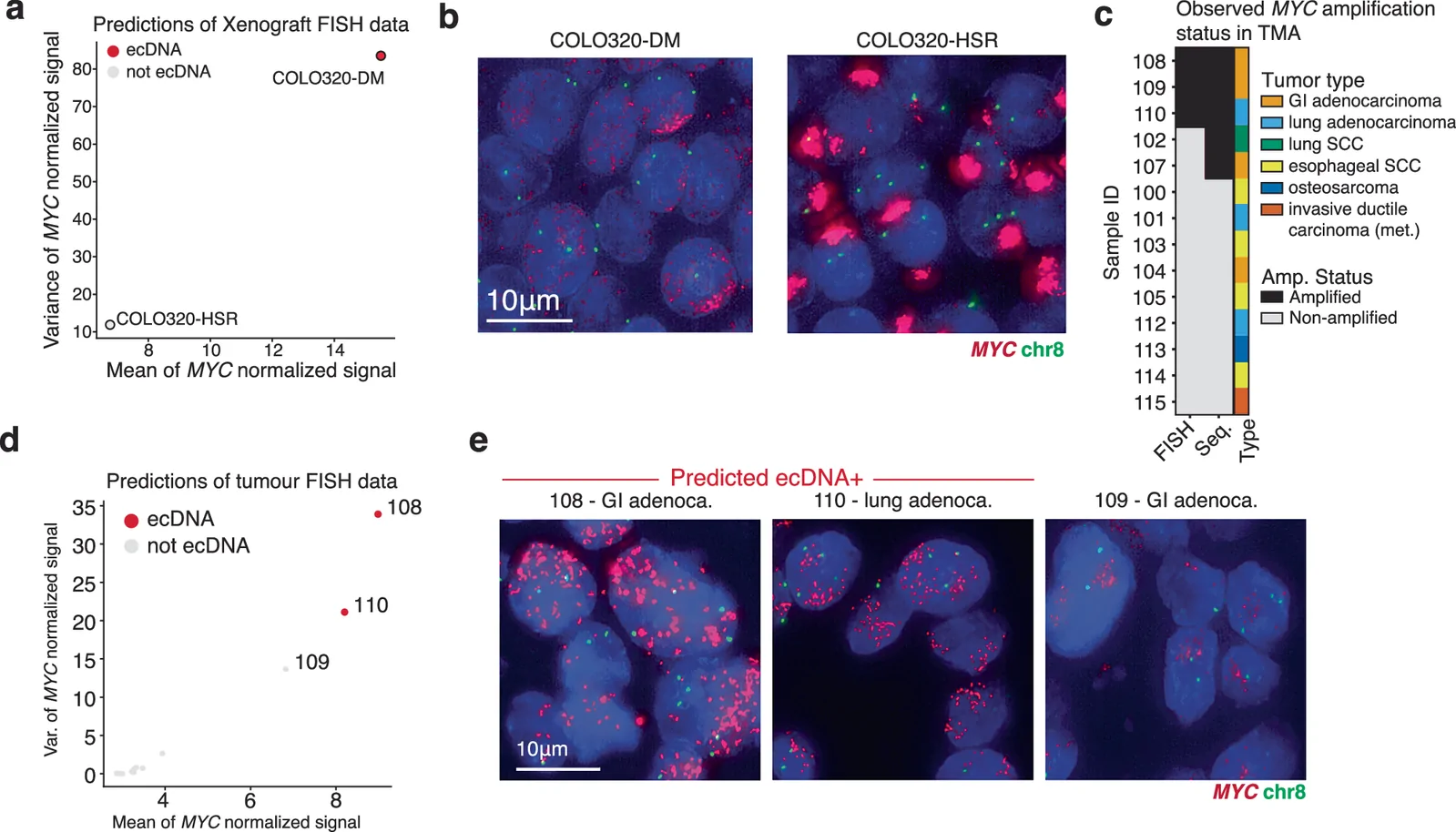
Proof-of-concept classification of ecDNA status in FFPE tumors. **a** Predictions of extrachromosomal DNA (ecDNA) status of *MYC* amplifications in xenograft COLO320DM and COLO320HSR tumors. Scatterplots summarize the mean and variance of normalized *MYC* copy-number distributions for COLO320DM and COLO320HSR xenograft tumors, as measured with DNA fluorescence in situ hybridization (FISH) on interphase cells; colors indicate predictions from *scAmp* (left). **b** Representative DNA FISH images from COLO320DM and COLO320HSR xenograft tumors (single replicate encompassing 10 fields of view, profiling 3875 and 2953 cells for COLO320DM and COLO320HSR, respectively); scale bars indicate 10 μm (right). Images were processed by small-volume computational clearing and maximum intensity projection. The brightness of individual channels was adjusted uniformly for both images. **c** Summary of Tissue Microarray (TMA) obtained from patient tumors; *MYC* amplification status as determined by panel sequencing and reported in the Stanford pathology archive (“Seq”) or DNA FISH (“FISH”) is reported for each sample. Black squares indicate the tumor was amplified by the respective assay. Tumor annotations are shown in distinct colors on the right-most bar. **d** Predictions of ecDNA status of *MYC* amplifications from TMA data profiled with interphase DNA FISH. **e** Representative interphase DNA FISH images from individual tumors of cancer cells are shown: Sample 108 (a gastrointestinal adenocarcinoma [“GI adenoca.”]; 2 cores and 8 fields of view profiling 1519 cells) and Sample 110 (a lung adenocarcinoma [“lung adenoca.”]; 1 core and 4 fields of view profiling 805 cells) were predicted to be ecDNA+; Sample 109 (a gastrointestinal adenocarcinoma; 2 cores and 7 fields of view profiling 1490 cells) is predicted to be chromosomally amplified by *scAmp*. Scale bar indicates 10 μm. Images were processed by small-volume computational clearing and maximum intensity projection. The brightness of individual channels was adjusted uniformly for all three images. Source data are provided as a Source Data file.

Finally, we hypothesized that *scAmp’s* copy-number-based approach to assessing ecDNA status could have applications beyond single-cell genomic data. Most archival patient tumors are stored as formalin-fixed paraffin-embedded (FFPE) tissue, which precludes the use of metaphase FISH for determining ecDNA status, as metaphase FISH requires actively cycling cells. Furthermore, although tumor bulk WGS and single-cell ATAC-seq are not part of routine clinical practice, DNA FISH on FFPE tissue is routinely used in clinical cytogenetics laboratories for assessing oncogene copy number. Therefore, we asked whether *scAmp* has the potential to generalize beyond single-cell genomic assays to enable the classification of ecDNA+ tumors from clinically relevant modalities like DNA FISH in FFPE. To establish the utility of *scAmp* for ecDNA calling in FFPE tissue, we first confirmed that *scAmp* could accurately distinguish ecDNA amplifications from chromosomal amplifications in FFPE tissue from mouse xenografts of COLO320DM and COLO320HSR cells (Fig. 4a, b; “Methods”). In order to control for potential segmentation effects, we normalized the *MYC* signal to the centromere 8 signal prior to *scAmp* analysis. We next extended these results to demonstrate the feasibility of this approach on patient tissues with a proof-of-concept analysis of a tissue microarray (TMA) generated from 14 tumors reported to have focal amplifications in the Stanford Pathology archive, including five tumors reported to amplify *MYC* (Fig. 4c). In the ~1 mm tissue cores represented on the TMA, we detected *MYC* ecDNA amplifications in 2 samples and *MYC* chromosomal amplification in 1 sample, consistent with the overall appearance of FISH signal in these tumors (Fig. 4d, e; “Methods”). These experiments demonstrate the feasibility of *scAmp* as a tool for ecDNA detection from DNA FISH, though further study will be needed to establish its generalizability and robustness.

## Discussion

Oncogene amplification on ecDNA has emerged as a hallmark of the most aggressive cancers^4,5,7^, where ecDNA’s random mitotic inheritance and unique regulatory state promote tumor progression. Here, by leveraging the fact that ecDNA is inherited randomly during mitosis, we have developed *scAmp*, a platform for unbiased detection and analysis of ecDNA from single-cell data. Combining synthetic datasets with single-cell experimental datasets of well-characterized cell lines and patient tumor samples, we demonstrate *scAmp*’s accuracy and utility in retrospective studies.

Our analyses with *scAmp* revealed several insights into ecDNA dynamics. First, in our analysis of cell lines, we found that there exist a class of chromosomal amplifications that are misclassified by bulk assays but are correctly predicted with *scAmp* (e.g., *ERBB2* in BT474 and *MYC* in Sw620; Supplementary Fig. 7). These data suggest that some chromosomal amplifications may have historically been ecDNA (perhaps related to the plasticity of amplification modes in the course of drug treatment^14,21,27^) and underscore that single-cell data will be critical for resolving these cases. Second, in our retrospective analysis of patient tumors profiled with scATAC-seq, we found that ecDNA+ cancer cell fractions were highly variable (ranging between ~10% to 100%) and that some tumors harbored evidence of ongoing subclonal evolution of new ecDNA (Fig. 3c, d). This finding is reminiscent of the ongoing evolution of ecDNA amplifications observed medulloblastoma^28^ and glioblastoma^29^ and, along with other recent large-scale analyses of scWGS data^18^, points to the utility of single-cell assays to study the relative timing and influence of amplification evolution in cancer. Third, we leveraged the molecular insights gained from scATAC-seq to identify putative gene expression programs associated with ecDNA, including increased glycolysis and hypoxia signaling (Supplementary Fig. 9). Finally, we introduce a proof-of-concept analysis demonstrating that *scAmp*’s principles may also enable patient stratification using measurements gained from clinically relevant modalities like DNA FISH on FFPE samples.

Beyond the potential of *scAmp,* there are important limitations and future directions to acknowledge. First, *scAmp’*s workflow relies on predicting the ecDNA status of individual genes and then heuristically labeling cells based on copy-number. This may conflate cells that have chromosomal amplifications with those that contain ecDNA, and thus, new algorithms that deconvolve these two populations will be useful. Second, while scATAC-seq has been used widely for copy-number estimation^31–33^, it is sparse and does not provide as robust copy-number quantification as scWGS approaches that directly measure genetic content^16,34^. Third, the benchmarking of *scAmp* in tumors from the TCGA dataset is limited by the lack of gold-standard validation of ecDNA status (i.e., DNA FISH on metaphase spreads). Lastly, while our analysis of DNA FISH in FFPE tissue is promising, it remains a proof-of-concept and generalizability to clinical samples will require more thorough benchmarking.

Our understanding of ecDNA was accelerated by the development of algorithms for the analysis of sequencing data and large pan-cancer sequencing studies. Likewise, we believe that *scAmp* offers a technical advance that will enable investigators to use high-resolution technologies to gain fundamentally new insights into ecDNA biology.

## Methods

### Ethics statement

This study complies with all relevant ethical regulations. Human tumors profiled with DNA FISH were identified from the Stanford Pathology archive in accordance with IRB protocol #69198, which was approved by the Stanford University Institutional Review Board. Xenograft mouse experiments received ethical approval from Stanford APLAC/IACUC Protocol #: 34041.

### Generating simulated training data

To generate data for training models, we simulated trajectories of genes that were amplified on either ecDNA or chromosomes (“HSRs”) using a modified Cassiopeia simulation framework^35^ (see section “**Simulation of ecDNA and HSR copy-number distributions”**). We simulated 1000 trajectories for both 1 M and 500 k cells (a total of 2000 trajectories): for each trajectory, we obtained copy-number for an ecDNA amplicon and an HSR amplicon for each cell (copy-numbers are denoted as ${c}_{i}^{a}$ for cell *i* and amplicon *a*). Then, we created 10,000 training datasets by sampling a random trajectory of ecDNA and HSR amplicons and applying a noise model to the observed copy-numbers per cell to account for noise in the sampling and copy-number estimation process. The noise model consisted of simulating an under-dispersed normal distribution parameterized by the observed counts: specifically, given an observed copy-number ${c}_{i}^{a}$ for cell *i* and amplicon *a*, we drew modified counts from the following distribution:

1 $${\widetilde{c}}_{i}^{a}\sim N(x={c}_{i}^{a},{scale}={sqrt}\left({c}_{i}^{a}*\left(1-p\right)\right))$$

where $p\sim {Unif}({{\mathrm{0.1,1}}})$. Importantly, we elected to simulate under-dispersed copy-numbers by inspecting the distributions of HSR-amplified genes in real single-cell data.

We additionally simulated 500 cell populations without any amplification by drawing copy-numbers from a mixture model as follows:

2 $${{c}_{i}}^{n}\sim {Poisson}(\lambda=2)+\max (0,N({{\mathrm{0,1}}}))$$

Finally, we simulated two mixtures of ecDNA/HSR and amplified/non-amplified cell populations: first, we simulated 10,000 mixtures of cells that amplified both ecDNA and HSR genes by merging copy-number distributions for both ecDNA and HSR and a random proportion. Second, we simulated 1000 mixtures of non-amplified cells and cells that either amplified ecDNA or HSR genes. The same noise model was applied to copy-numbers for these mixtures as above. Altogether, this resulted in 32,500 training examples: 10,000 ecDNA distributions; 10,000 HSR distributions; 10,000 ecDNA/HSR distributions; 1000 normal/ecDNA distributions; 1000 normal/HSR distributions; and 500 normal distributions.

### Simulation of ecDNA and HSR copy-number distributions

We used a modified evolutionary model of ecDNA copy-number dynamics^11,14^ to simulate trajectories of both ecDNA and HSR amplifications. Simulations began with a single cell with ${k}^{e}$ ecDNA and ${k}^{c}$ chromosomal copies of a given gene, as determined below:

3 $${k}^{e}\sim {Poisson}(\lambda=3)$$

4 $${k}^{c}\sim {NegativeBinomial}(n=3,p=0.4)$$

Simulations were additionally parameterized with selection coefficients acting on both ecDNA+ and HSR+ cells (${s}_{e},{s}_{c}$, respectively); a base birth rate (${\lambda }_{{base}}=0.5$); and a death rate (μ=3).

Evolutionary simulations then proceeded as previously described^11,14^: starting with the parental cell, a birth rate is defined based on the selection coefficient acting on the cell (a combination of ${s}_{e}$ and ${s}_{c}$, depending on whether the cell has ecDNA and/or chromosomal copy amplifications) as ${\lambda }_{1}={\lambda }_{{base}}\times (1+{s}^{{\prime} })$ where ${s}^{{\prime} }$ is the combination of selection coefficients from both ecDNA and HSR. Then, a waiting time until the next cell division is drawn from an exponential distribution, parametrized by the updated birth rate:${t}_{b}\sim \exp (-{1/\lambda }_{1})$. Time to cell death is also drawn from an exponential distribution:${t}_{d}\sim \exp (-\mu )$. When ${t}_{b} < {t}_{d}$, a cell division event is simulated and a new edge is added to the growing phylogeny with edge length ${t}_{b}$; otherwise, the cell dies, and the lineage is stopped. This process will continue until a user-defined stopping condition is specified: in this case, the number of extant (living) cells is either 1 M or 500 k.

During a cell division, ecDNAs are split among daughter cells randomly, as previously described and validated^11^, generating new daughter cell ecDNA counts of ${k}_{{d}_{1}}^{e}$ and ${k}_{{d}_{2}}^{e}$ for ${d}_{1}$ and ${d}_{2}$. Specifically,

5 $${k}_{i}^{e}\sim {binomial}(2{N}^{e},0.5)$$

Where *N*^*e*^ is the ecDNA copy-number of the parent. The other daughter cell's copy-number is the remaining ecDNA copies from the parent. Conversely, the chromosomal copy number of the daughter cells is kept constant, assuming faithful chromosome inheritance^15^.

### Creating ground-truth cell line annotations

We created validation datasets by first curating data from previously characterized cell lines. We elected to focus on 11 cell lines that were known to contain either ecDNA or chromosomal amplifications, per previous characterization by DNA FISH on metaphase spreads (as detailed in Supplementary Fig. 1b, c): COLO320DM, COLO320HSR, GBM39ec, GBM39HSR, GBM39KT, SNU16 (and SNU16m1), H2170, Sw620, TR14, and BT474. While several genes are amplified on a given ecDNA or HSR, we elected to evaluate a model’s performance primarily on its ability to successfully predict the status of a well-characterized gene, including *MYC, EGFR, FGFR2, ERBB2, NKX2.1, MYCN*, and *MDM2*. We report the dispersion of copy-numbers for these genes in their relevant cell lines (based on amplification status as determined by DNA FISH on metaphase spreads in Supplementary Fig. 1**)** to the simulated copy-number trajectories for ecDNA+ and ecDNA- genes in Supplementary Fig. 6a.

### Compiling TCGA annotations for concordance analysis

We compiled annotations of gene amplification status from WGS data sequenced through The Cancer Genome Atlas (TCGA). Specifically, we utilized results from the AmpliconSuite^6,10^ pipeline, which detects genomic amplifications and classifies them as ecDNA or other forms of amplifications (e.g., complex non-cyclic or linear); these results were previously published^3,6,14^. Briefly, this approach for detecting ecDNA uses three steps wrapped in the AmpliconSuite pipeline (https://github.com/AmpliconSuite/AmpliconSuite-pipeline, v1.1.1). First, given a BAM file, the analysis pipeline detects “seed” intervals where copy-number amplifications exist (CN > 4.5, with a size between 10 kb–10 Mb). Copy-numbers are pre-computed from the BAM file using a workflow such as cnvkit^36^. Second, AmpliconSuite invokes AmpliconArchitect^10^ (AA) to perform joint copy-number and breakpoint detection analysis in the focally-amplified regions, forming a copy-number-weighted breakpoint graph. AA then extracts paths from this breakpoint graph, each of which corresponds to a particular amplification, that collectively explain the copy-number and breakpoint patterns detected. Finally, a rule-based classification is performed using AmpliconClassifier^8^ based on the paths extracted by AA to predict the mode of focal amplification (ecDNA or other). This includes assessing structural variant types, copy-numbers and the structure of the path (e.g., if the path is circular or not). The complete classification criteria and description of the AmpliconClassifier tool are available in the supplementary information of ref. ^8^.

From the AmpliconSuite analysis, we created two annotations that we used for model evaluation: first, we annotated tumors by whether or not they had any ecDNA amplification (sample-level); and second, within each tumor we annotated which genes were predicted to be amplified on ecDNA based on AmpliconSuite analysis (gene-level). We evaluated model concordance on both criteria, as reported in Supplementary Fig. 5.

### Training and benchmarking *scAmp*

We built models that predict gene amplifications as either ecDNA+ or non-ecDNA based on a featurization of single-cell copy-number distributions. While in principle the copy-number distribution could be featurized in many ways, in this proof-of-concept work, we elected to reduce this distribution to simple statistics. Specifically, given a distribution of copy-numbers of cells, *C* = {*c*_1_, …, *c*_*n*_}, we extracted 14 features (*X*): the mean, variance, coefficient of variation (variance / mean), the deciles of the copy-number distribution, and the inter-quartile range. While computing these statistics, we only considered “amplified” cells in which the copy-number exceeded 2.

Using these features, we trained fully-connected neural networks (Multilayer Perceptrons [MLPs]) using pytorch^37^ (v2.6.0.dev20241219). We performed hyperparameter searches across the following parameters (Supplementary Fig. 2):

- Number of layers: [0, 1, 2, 3, 4] (0 corresponding to a basic logistic regression)
- Size of hidden layers: [10, 20, 30]
- Learning rates: [1e-3, 1e-4]
- Activation functions: [“relu”, “tanh”, “sigmoid”]
- Batch size: [64, 256, 4096]

We trained models across 400 epochs using the simulated copy-number data (see above “**Generating simulated training data**”) and assessed performance on held-out single-cell data from cell lines and patient tumors (see above “**Creating ground-truth cell line annotations”** and “**Creating ground-truth TCGA annotations”**, respectively). For the purposes of this supervised learning task, we classified any mixed HSR/ecDNA sample that had at least 20% ecDNA+ cells as ecDNA+, and otherwise non-ecDNA. We trained 5 models, each with different seeds, to assess the robustness of the model. We prioritized models based on both the per-sample AUROC and accuracy of predictions on patient tumor data (i.e., predicting whether or not a patient had *any* gene amplified on ecDNA). The final *scAmp* model (v1.0.0) consisted of 3 hidden layers with 20 neurons each and a “sigmoid” activation function (nn.Sigmoid). While training, we used a learning rate of 1e-4, a batch size of 64, and a dropout rate of 0.1 to reduce overfitting.

We also benchmarked *scAmp* against a RandomForest classifier (trained with sklearn version 1.6.0 using default parameters) and two “null” linear models trained on either the mean copy-number (“mean null”) or the mean and standard deviation of the copy-number distribution (“mean+std null”). We report the performance of null models on held-out cell line data in Supplementary Fig. 4 and compare the concordance of a RandomForest classifier to *scAmp* on held-out tumor data in Supplementary Fig. 5.

### Stress-testing *scAmp* model

In addition to the experiments described above, we also stress-tested *scAmp* on two additional experiments. First, we tested *scAmp* on in silico mixtures of ecDNA+ and chromosomally-amplified (but ecDNA−) cells. To do this, we utilized amplified genes in isogenic ecDNA+ and HSR+ cell line pairs: *MYC* in the COLO320DM (ecDNA+) and COLO320HSR (ecDNA−) pair and *EGFR* copy-numbers in the GBM39ec (ecDNA+) and GBM39HSR (ecDNA−) pair. We simulated cell populations of 1000 cells consisting of 0%, 5%, 10%, 15%, 20%, 30%, 40%, 50%, 75%, or 100% ecDNA+ cells; ecDNA+ and ecDNA− cells were sampled at random, without replacement, from the respective cell line. We simulated 5000 replicates. From this dataset, we used *scAmp*’s default parameters to predict if a simulated population had ecDNA. We report the accuracy and the probability of each replicate in Supplementary Fig. 6b.

In the second experiment, we assessed model performance in increasingly noisy input data. To perform this experiment, we took the featurized matrix of simulated copy-number data that is used for input to *scAmp* and added Gaussian noise to the matrix (defined as *ϵ* ~ *N*(0, *σ*) for *σ *∈ {0, 1, 2, 5, 10, 20, 30, 50, 100}). We assessed AUROC on predicting the ecDNA status of a simulated population and report performance for the final *scAmp* model, a RandomForest classifier, and the mean null model (Supplementary Fig. 6e).

### Cell culture

The BT474 cell line was obtained from American Type Culture Collection (ATCC) [Cat# CRL-3247] and was cultured in standard culture media: RPMI 1640 (Gibco, A1049101), 10% fetal bovine serum (FBS; Gibco A5670401), 1% penicillin/streptomycin (Thermo Fisher Scientific, 15140-122), 1% HEPES (Thermo Fisher Scientific, 15630080), 1% Glutamax (Thermo Fisher Scientific, 35050061) at 37 °C in 5% CO_2_. The H2170 cell-line was obtained from American Type Culture Collection [Cat# CRL-5928] and was cultured in Dulbecco’s modified Eagle’s medium (DMEM; Thermo Fisher Scientific, 11995073), 10% FBS and 1% penicillin–streptomycin at 37 °C in 5% CO_2._ TR14 cells were a kind gift from J.H. Shulte and were cultured in RPMI 1640 + 10% FBS and 1% PSQ.

### Metaphase DNA FISH

Metaphase spreads for the following cell lines were performed as part of previous studies: COLO320DM/HSR^38^, GBM39KT and SNU16m1^14^, GBM39ec/HSR^11^, *ERBB2* and *MYC* FISH in H2170 cells^15^. BT474, H2170, and TR14 cells were arrested in metaphase with 0.1 µg/mL colcemid for 2–4 h, collected by centrifugation, and washed once with phosphate-buffered saline (PBS). The cell pellet was then incubated in 0.075 M KCl for 20 min at 37 °C to induce hypotonic swelling. Cells were subsequently fixed and washed three times with freshly prepared fixative consisting of methanol and glacial acetic acid (3:1, v/v). Fixed cells were resuspended in an appropriate volume of fixative and dropped from a height of several inches onto pre-humidified glass slides to achieve optimal chromosome spreading.

Slides were rinsed with PBS, washed in 2 × saline-sodium citrate (SSC), and sequentially dehydrated in 70%, 85%, and 100% ethanol. Hybridization probe solution was applied, and the slides were denatured at 75 °C for 3 min followed by hybridization overnight at 37 °C. Post-hybridization washes were performed in 2 × SSC containing 0.1% Tween-20 and PBS. Slides were then mounted with ProLong™ Gold Antifade Mountant containing DAPI. Images were acquired on a Leica Thunder widefield microscope using a 63 × oil objective and LAS X software.

### Paired scATAC-seq and scRNA-seq library generation

Single-cell paired RNA-seq and ATAC-seq libraries were generated with the 10X Chromium Single-Cell Multiome ATAC + Gene Expression kit according to the manufacturer’s protocol. Libraries were sequenced on an Illumina NovaSeq 6000 system. Single-cell Multiome data for other cell lines were obtained from the following public databases: COLO320DM & COLO320HSR (GEO accession GSE159986), GBM39-KT (SRA accession SRS21730888), SNU16 (SRA accession SRS21730887), SNU16m1 (SRA accession SRS21730902), Sw620 (Genome Sequence Archive accession PRJCA021248) and TR14 (SRA accession SRS21730907).

### Copy-number analysis of scATAC-seq data

We calculated copy-numbers in 3 Mb windows based on the background ATAC-seq signal as we previously described and validated^14,22,31^. Briefly, we determined read counts in large intervals across the genome using a sliding window of 3 Mb moving in 1 Mb increments across the reference genome (hg19). Genomic regions with known mapping artifacts were filtered out using the ENCODE hg19 blacklist. For each interval, insertions per bp were calculated and compared to 200 of its nearest neighbors with matched GC nucleotide content. The mean log_2_[fold change] was computed for each interval. Based on a diploid genome, copy numbers were calculated using the formula

6 $${CN}=2\times {2}^{{\log}_2[{FC}]}$$

where CN denotes copy number, and FC denotes mean fold change compared to neighboring intervals. To query the copy numbers of a gene, we obtained all genomic intervals that overlapped with the annotated gene sequence and computed the mean copy numbers of those intervals.

### Analysis of scATAC-seq data from cell lines

Single-cell Multiome data was aligned using cellranger-arc count (10X Genomics, v. 1.0.0); for BT474, we aligned directly to the hg19 genome; for H2170, Sw620, GBM39ec, and GBM39HSR, we lifted over fragments aligned to hg38 to hg19 using the UCSC hg38tohg19 chain (hg38ToHg19.over.chain.gz) in order to integrate this data with our existing cell-line atlas. For the purposes of this study, we focused on the scATAC-seq fraction of the data and performed analysis using ArchR^39^ (v. 1.0.2). Cells with fewer than 1000 unique fragments or TSS enrichment less than 4 were filtered out. Doublets were removed using ArchR’s *addDoubletScores* and *filterDoublets* commands with the following parameters: *k* = 10, knnMethod = “UMAP”, LSIMethod = 1. Dimensionality reduction was performed using Iterative Latent Semantic Indexing with the *addIterativeLSI* function. For each cell line, we converted single-cell copy-number distributions of amplified genes (those with average copy-number exceeding 2.5) to *scAmp*-compatible features with the *prepare_copy_numbers* function with the following parameters: min_copy_number = 3, max_percentile = 99.9. Genes with predicted likelihood exceeding 0.6 were marked as ecDNA+.

### Predicting gene amplification status from TCGA scATAC-seq data

Single-cell ATAC-seq data for patients from TCGA were obtained from NCI Genomic Data Commons (https://gdc.cancer.gov/about-data/publications/TCGA-ATAC-Seq-2024), and copy-numbers were computed as described above (see section entitled “**Copy-number analysis of scATAC-seq data”)**. For each tumor sample, we compared the average copy-numbers of genes derived from scATAC-seq data to the copy-numbers inferred from AmpliconArchitect analysis on bulk WGS data and found correlations above 0.5 (Supplementary Fig. 3c). For each tumor, we converted single-cell copy-number distributions of amplified genes (those with average copy-number exceeding 2.5) to *scAmp*-compatible features with the *prepare_copy_numbers* function with the following parameters: min_copy_number = 3, max_percentile = 99.9. Genes with predicted likelihood exceeding 0.6 were marked as ecDNA+.

To quantify the frequency of ecDNA+ cells in Fig. 3c, we computed the fraction of cells that amplified a gene predicted to be on ecDNA with more than 4 copies. We report the stability of these quantifications in Supplementary Fig. 9d.

### Analysing correlates of ecDNA status in TCGA cohort

To analyse the shifts in immune and stromal composition based on the ecDNA status of a given tumor, we first separated immune and stromal populations from epithelial populations using annotations provided by the authors of the original study^26^. (Supplementary Fig. 9a, b). We then evaluated the frequency of each immune or stromal cell subset stratified by tumor type and whether the tumor carried any genes predicted to be amplified on ecDNA by *scAmp*.

To assess the transcriptional changes in ecDNA+ cancer cells, we focused specifically on the cancer cell fraction and tumors with genes predicted to be amplified on ecDNA. We computed module scores for each signature in the MSigDB^40^ Hallmark set (available at https://www.gsea-msigdb.org/gsea/msigdb/human/genesets.jsp?collection=H) with ArchR (version v. 1.0.2) using the *addModuleScore* function with useMatrix = ’GeneScoreMatrix’. We then performed in silico separation of cancer cells based on whether they amplified any gene predicted to be on ecDNA at more than 4 copies. To find generalizable patterns, we computed the average signature score across tumors, performed Z-normalization to compare across signatures, and assessed differential signature scores based on the in silico separation of ecDNA status.

### Analysis of GBMx sample TCGA−06−A7TK−01A

To analyse the subclonal evolution of the GBMx sample TCGA−06−A7TK−01A, we first inspected the shared patterns of copy-number amplifications in cancer cells computed across 3 Mb windows (see above “**Copy-number analysis of scATAC-seq data**”). To identify regions that were likely ecDNA+, we focused on 3 Mb regions that were amplified at greater than 7 copies in at least 5% of cells. We then performed hierarchical clustering of cells based on these regions using Euclidean distance and “ward” linkage, and extracted 4 clusters based on tree depth.

To create the single-cell UMAP projection of this patient’s data, we performed analysis with ArchR (version 1.0.2). We first performed dimensionality reduction using Iterative Latent Semantic Indexing with the *addIterativeLSI* function with the following parameters: useMatrix = ‘TileMatrix’, iterations = 2, clusterParams = list(resolution = c(0.2, 0.4), n.start=10), and varFeatures = 30000. We computed a UMAP projection from the LSI dimensions using nNeighbors = 15, minDist = 0.2, and metric = ‘cosine’. Genome tracks were visualized using the *plotBrowserTrack* function using tileSize = 100, upstream = 1000000, and downstream = 1500000.

### Xenografts

Female Foxn1nu Athymic Nude mice (Charles River Laboratories) were housed under standard conditions. 10 mice were used, with 5 mice housed in each cage (COLO320DM, COLO320HSR). Mice were 4 to 6 weeks old at the start of experiments (DOB: March 21, 2022; Start date: April 28, 2022). Mice were anesthetized using isoflurane in an induction chamber immediately before subcutaneous cancer cell injection. 500,000 cells suspended in 100 µL of a 1:1 PBS:Matrigel mixture were injected subcutaneously into each flank bilaterally using a 25-gauge needle and a 1 mL syringe. Mice were monitored for signs of distress or complications after injection. Tumor length and width were measured several times per week until Day 21, at which time mice were sacrificed using a sealed CO₂ chamber, with cervical dislocation performed as a secondary confirmatory method. Maximal tumor size permitted by Stanford APLAC/IACUC was 2000 mm^3^, which was not exceeded in any instance. Tumors were sharply dissected from the subcutaneous space, immediately flash-frozen in liquid nitrogen, and stored at −80 °C. Excised tumor tissues were sectioned onto glass slides by the Stanford Human Pathology/Histology Service Center. The study received ethical approval from Stanford APLAC/IACUC Protocol #: 34041.

### DNA FISH on FFPE

Tumors with amplified oncogenes were identified from the Stanford Pathology archive in accordance with IRB protocol #69198, which was approved with the Stanford University Institutional Review Board. DNA FISH was performed using the Agilent Technologies histology FISH accessory kit (K579911-5) according to the manufacturer’s instructions. Briefly, slides were incubated at 60 °C for 1 h prior to deparafinization in histoclear (2 × 5 min) and rehydration (2 x 2 min in 100% ethanol, 2 x 2 min in 70% ethanol). Samples were incubated in pre-treatment solution in a vegetable steamer for 20 min and then cooled at room temperature for 15 min. Samples were washed twice in wash buffer for 3 min. Samples were then treated with ready-to-use pepsin drops for 20 min at room temperature, followed by 2 x 3 min wash buffer incubations. Samples were dehydrated with a graded series of ethanol: 2 min each 70%, 85%, 100% ethanol. FISH probes targeting *MYC* (RP11-440N18) and the chromosome 8 centromere (CHR08-20-GR) with hybridization buffer were purchased from Empire Genomics. After application of FISH probes diluted in hybridization buffer, slides were incubated at 82 °C for 5 min and then overnight at 37 °C in a humid chamber. Samples were then incubated in stringent wash buffer at 65 °C for 10 min, and then in wash buffer for 2 x 3 min at room temperature. Slides were dehydrated in a series of ethanol washes: 2 min each in 70%, 85%, and 100% ethanol. After air-drying, samples were mounded with the provided fluorescence mounting medium. Images were acquired on a Leica Thunder widefield microscope using a 63× oil objective using LAS X software. Z-stack images were post-processed using Small Volume Computational Clearing on the LAS X Thunder Imager prior to generating maximum intensity projections. Image analysis was performed using the Aivia 15 (patient tumors) and Aivia 13 (xenograft) software. Green and red pixel classifiers were used to distinguish centromere 8 and *MYC* signals, respectively. Fields of view from the same sample were merged to facilitate processing. For sample 110, one field of view was excluded from FISH quantification and downstream analysis due to globally poor segmentation. Segmentation was performed using the Cellpose module in Aivia 15 (patient tumors) and Aivia 13 (xenograft). Cells with circularity <0.4 or nuclear area >150 µm were presumed to be poorly segmented and were filtered out prior to copy number analysis. Normalized *MYC* signal was computed by dividing the number of *MYC* foci by the number of chromosome 8 foci and passed into *scAmp* for ecDNA classification.

### Statistics and reproducibility

All statistical tests comparing the distribution of continuous values and accompanying p-values are indicated in the appropriate figure legend. Wilcoxon rank-sum tests were performed with R’s *wilcox.test* function. All boxplots present the quartiles of the distribution (with centers marking the median and lower and upper boundaries marking the 25^th^ and 75^th^ percentiles, respectively), and whiskers extend to 1.5× the interquartile distance. No statistical method was used to predetermine sample size. No samples or data points were excluded. The experiments were not randomized. The investigators were not blinded to the conditions of the experiments during data analysis.

### Reporting summary

Further information on research design is available in the Nature Portfolio Reporting Summary linked to this article.

## Supplementary information

Supplementary Information

Reporting Summary

Transparent Peer Review file

## Source data

Source Data

## Data Availability

The raw single-cell multiome sequencing data generated in this study have been deposited in the NCBI SRA database under BioProject accession code PRJNA1472115. The source imaging data have been deposited in the Stanford Digital Repository https://doi.org/10.25740/bx954rw4074. Other source data are provided with this paper. AmpliconClassifier outputs containing ecDNA coordinates in TCGA samples were previously published and are publicly available at figshare https://doi.org/10.6084/m9.figshare.24768555.v1. Previously published scATAC-seq data of cell lines were obtained from public repositories: GEO Series GSE159986 (COLO320DM and COLO320HSR), BioProject accession code PRJNA1127616 (GBM39KT, SNU16, SNU16m1, and TR14), and Genome Sequence Archive PRJCA021248 (Sw620). Previously published TCGA scATAC-seq data are publicly available via NCI Genomic Data Commons [https://gdc.cancer.gov/about-data/publications/TCGA-ATAC-Seq-2024]. Previously published metaphase FISH images for COLO320DM, COLO320HSR, SNU16, GBM39KT, and TR14 are available through the Stanford Digital Repository https://doi.org/10.25740/cp783mb3217 and https://doi.org/10.25740/ff315yn8920. Previously published metaphase FISH images for Sw620 are available through figshare [https://figshare.com/s/ab6a214738aa43833391]. Source data are provided with this paper.

## Code availability

The *scAmp* software used to develop the model and generate these results is publicly available and has been deposited on GitHub [https://github.com/JonesCompBioLab/scamp] under the MIT License. The specific version of the code associated with this publication is archived in Zenodo^41^ https://doi.org/10.5281/zenodo.20434847.
