# Supplementary material for: scAmp enables focal gene amplification analysis from single-cell data
Source: Nat Commun. 2026 Aug 8;17:9563. doi: 10.1038/s41467-026-76417-3 (PMC13550473; doi:10.1038/s41467-026-76417-3)
Supplement: Supplementary file 1 [file 41467_2026_76417_MOESM1_ESM.pdf]

SUPPLEMENTARY INFORMATION

SUPPLEMENTARY FIGURES

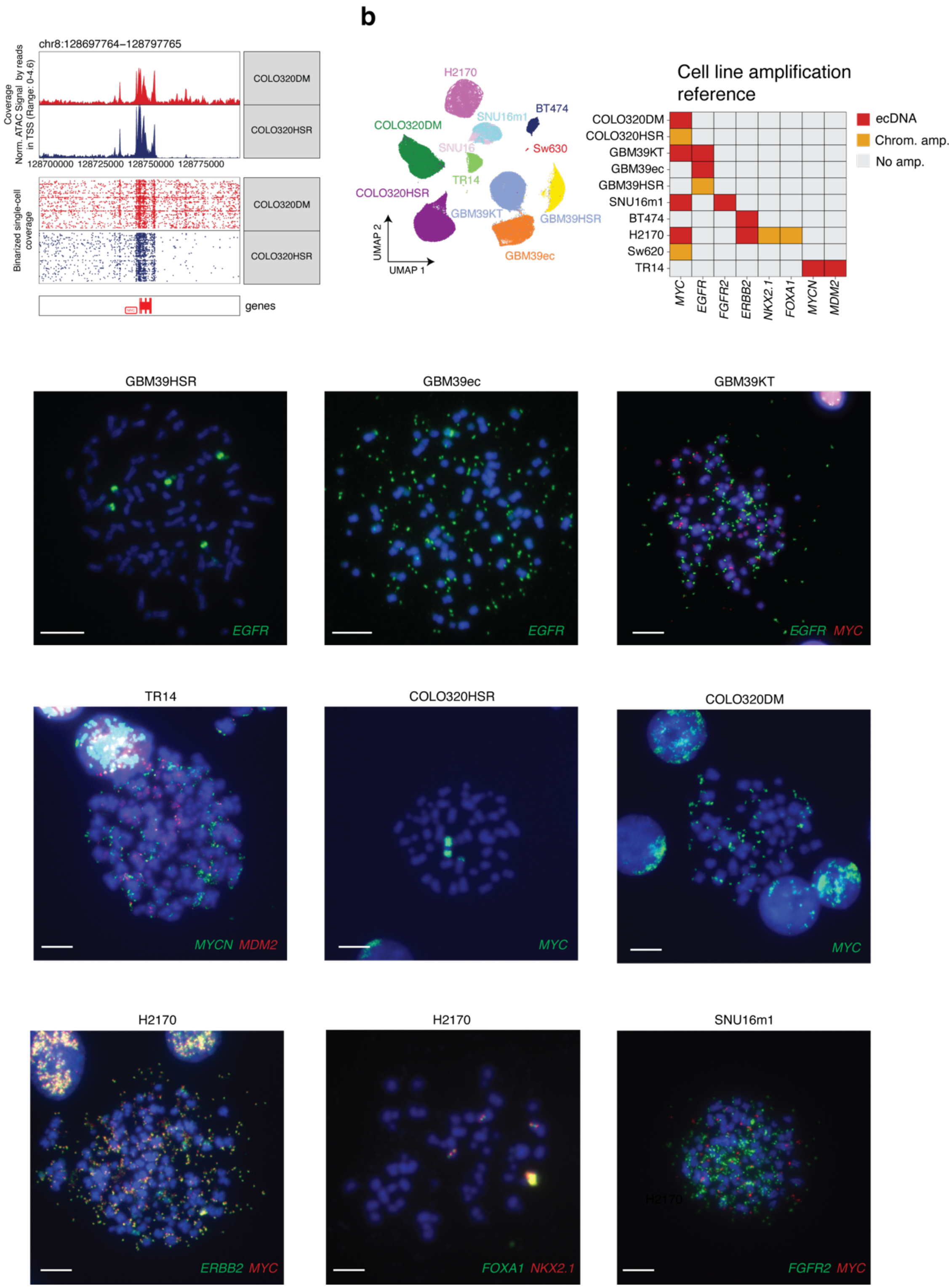

Supplementary Figure 1. Overview of cell line benchmarking datasets. (a) Single-cell assay for transposase-accessible chromatin by sequencing (scATAC-seq) coverage

for COLO320DM and COLO320HSR cell lines in the *MYC* locus. Pseudobulk coverage (top) and binarized coverage of individual cells (bottom) demonstrate that while the average signal is similar, the COLO320DM ATAC signal appears to have greater variability. **(b)** A UMAP projection of single-cell ATAC-seq data for cell lines used in this study (left) and a summary of the characterized amplification mode of specific genes (right). Characterization of amplifications is derived from prior reports. **(c)** Representative images of DNA fluorescence *in situ* hybridization (FISH) on metaphase spreads from previous studies demonstrating the amplification mode of key oncogenes used for testing model predictions. Scale bars indicate 10μm. ecDNA = extrachromosomal DNA.

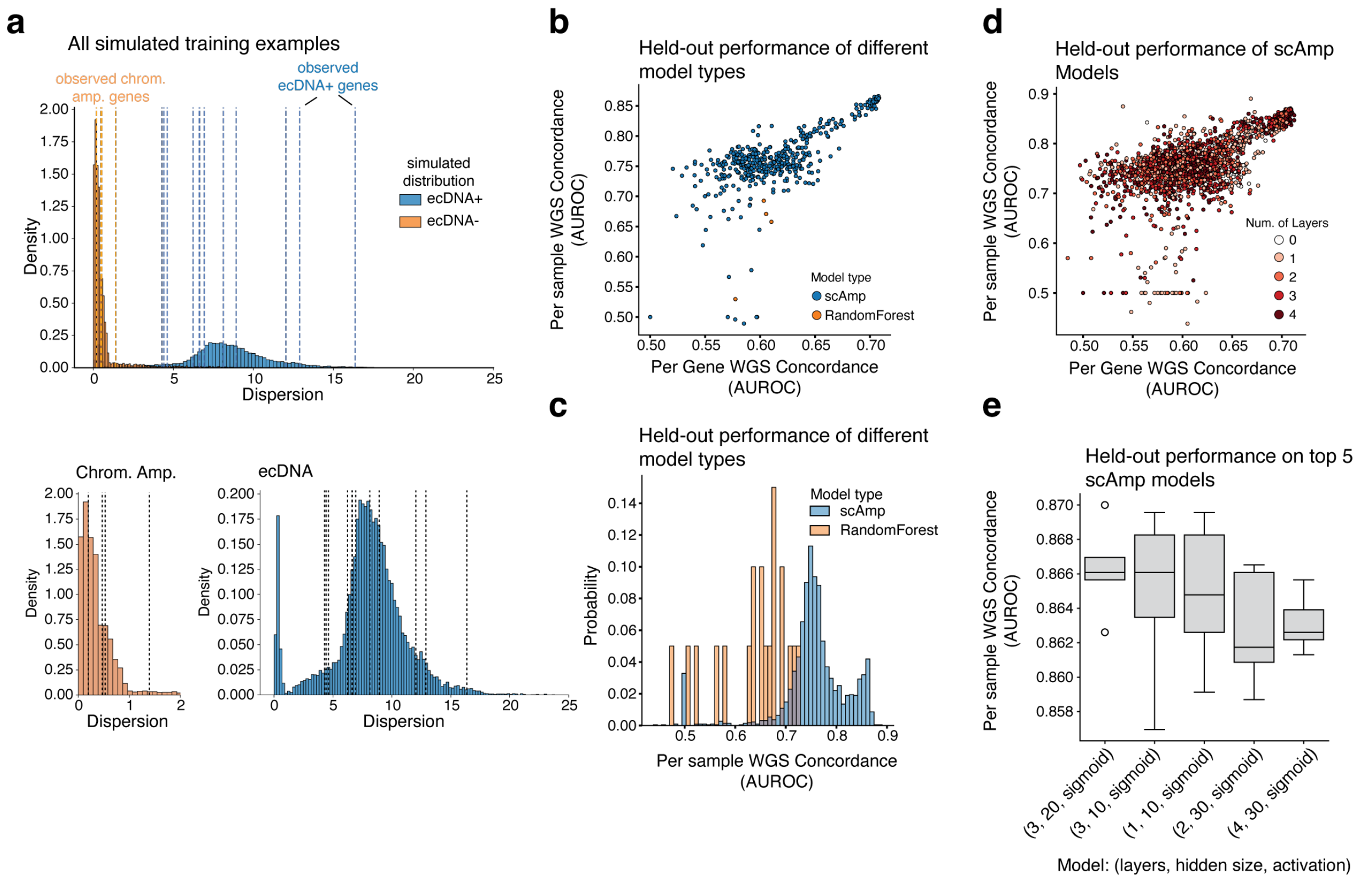

Supplementary Figure 2. Hyperparameter tuning of *scAmp* models. (a) Comparison of dispersion values between simulated and observed cell-line copy-number distributions, both the full distribution (top) and for chromosomal or extrachromosomal DNA (ecNDA) amplifications (bottom). In the full distribution, dispersions of observed ecDNA copy-number distributions are indicated in blue; dispersions of observed chromosomal amplification copy-number distributions are indicated in orange. **(b)** Comparison of performance for all trained *scAmp* and RandomForest classifier models, as quantified by area under the receiver operator characteristic (AUROC) of per-sample or per-gene ecDNA concordance with held-out patient tumour single-cell assay for transposase-accessible chromatin by sequencing (scATAC-seq) data from TCGA. Per-sample ecDNA concordance quantifies the agreement between a model and bulk WGS in predicting whether a patient has at least one ecDNA amplicon; per-gene ecDNA concordance quantifies the agreement between a model and bulk whole genome sequencing (WGS) of predicting whether a gene is amplified on ecDNA or not (**Methods**). **(c)** Histogram of

AUROC of per-sample ecDNA concordance on held-out patient tumour scATAC-seq data for both RandomForest classifiers and *scAmp* models. **(d)** Scatterplot of *scAmp* model performances colored by number of hidden layers in *scAmp* model. **(e)** Boxplot of the top 5 *scAmp* models, as measured by the AUROC on the per-sample ecDNA concordance task for held-out patient tumour samples. The number of layers, size of hidden layer, and activation function are indicated for each top model. Individual points indicate the performance of a training replicate (i.e., training a model with a new random seed). Boxplots report the quartiles of the distributions (with centers marking the median and lower and upper boundaries marking the 25th and 75th percentiles, respectively), and whiskers extend to 1.5x the interquartile distance. Source data are provided as a Source Data file.

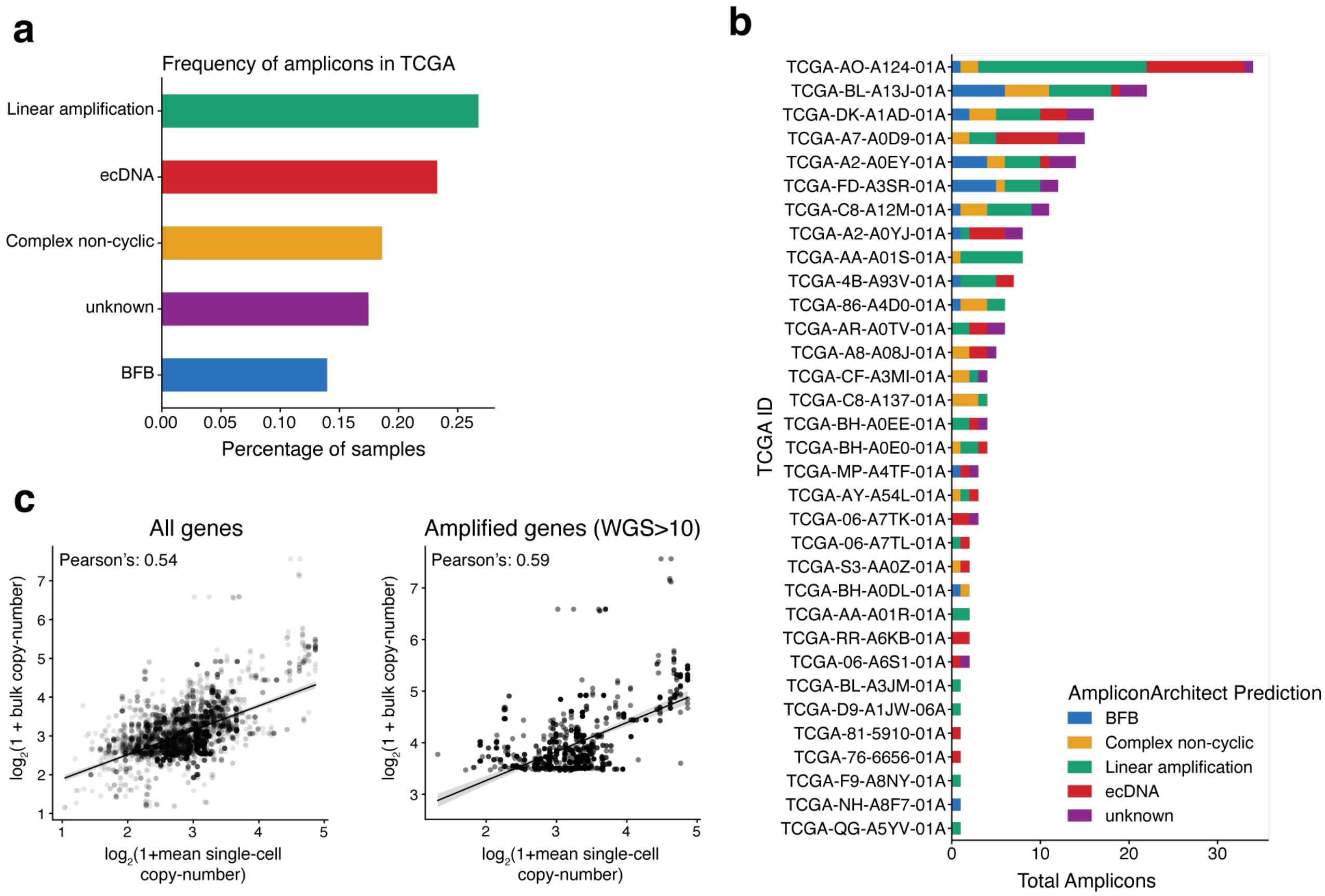

Supplementary Figure 3. Overview of single-cell tumour datasets from The Cancer Genome Atlas. (a) Summary of gene amplification annotations in patient tumours profiled with single-cell assay for transposase accessible chromatin by sequencing (scATAC-seq) from The Cancer Genome Atlas (TCGA), as obtained from whole genome sequencing (WGS) analysis with AmpliconSuite. **(b)** The composition of amplicons per patient sample profiled with scATAC-seq from TCGA, where amplicon type is inferred from AmpliconSuite analysis of WGS data. **(c)** Scatter-plot reporting the agreement between gene-level copy-numbers inferred from scATAC-seq and bulk WGS data. Plots and Pearson correlations (ρ) are reported for all genes (left; ρ = *0.54*) and just amplified genes (right; ρ = *0.59*). Source data are provided as a Source Data file.

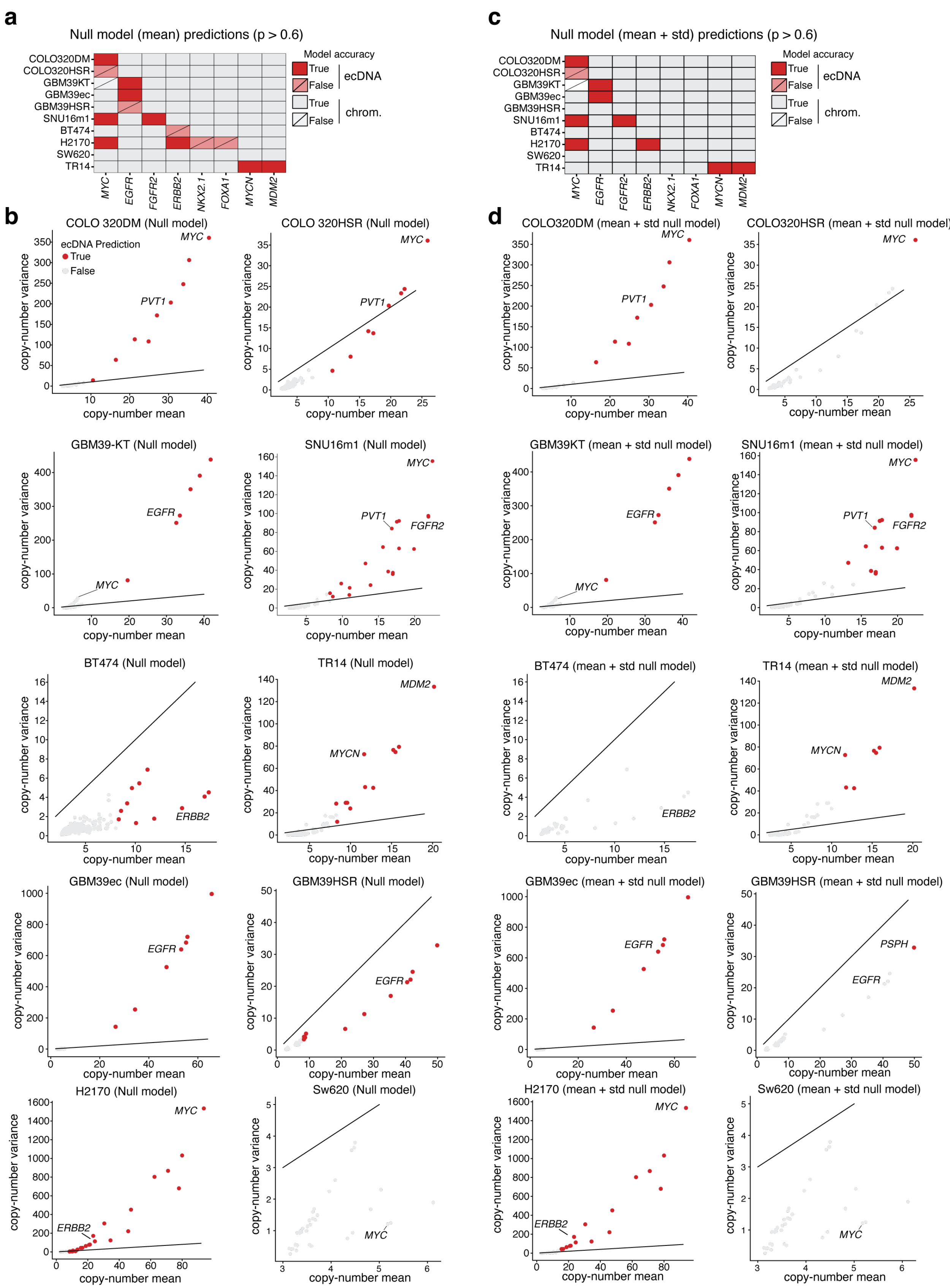
a
Null model (mean) predictions (p > 0.6)
COLO320DM
COLO320HSR
GBM39KT
GBM39ec
GBM39HSR
SNU16m1
BT474
H2170
SW620
TR14
MYC
EGFR
FGFR2
ERBB2
NKX2.1
FOXA1
MYCN
MDM2
Model accuracy
True
False
ecDNA
True
False
chrom.
c
Null model (mean + std) predictions (p > 0.6)
b
COLO 320DM (Null model)
ecDNA Prediction
True
False
COLO 320HSR (Null model)
GBM39-KT (Null model)
SNU16m1 (Null model)
BT474 (Null model)
TR14 (Null model)
GBM39ec (Null model)
GBM39HSR (Null model)
H2170 (Null model)
Sw620 (Null model)
d
COLO320DM (mean + std null model)
COLO320HSR (mean + std null model)
GBM39KT (mean + std null model)
SNU16m1 (mean + std null model)
BT474 (mean + std null model)
TR14 (mean + std null model)
GBM39ec (mean + std null model)
GBM39HSR (mean + std null model)
H2170 (mean + std null model)
Sw620 (mean + std null model)
PVT1
PSPH
copy-number variance
copy-number mean

Supplementary Figure 4. Null model performance on held-out cancer cell line data. (a-b) Performance of mean-only null linear model (predicting extrachromosomal DNA [ecDNA] status from mean copy-number) on held-out cell line data. **(a)** Summary table of null model predictions of focal genes across cell lines used in this study. Red boxes indicate an ecDNA prediction; grey boxes indicate a chromosomal amplification prediction. Hash marks indicate an incorrect prediction. **(b)** Scatterplots summarizing the mean and variance of gene copy-number distributions and null model predictions in cell lines used in this study. Each point is a single gene, colours indicate model prediction, and focal genes are annotated. **(c-d)** Performance of mean and standard deviation ("mean+std") null linear model (predicting ecDNA status from mean and standard deviation of copy-number distribution) on held-out cell line data. **(c)** Summary table of mean+std null model predictions of focal genes across cell lines used in this study. Colours and annotations are the same as in (a). **(d)** Scatterplots summarizing the mean and variance of gene copy-number distributions and mean and standard deviation ("mean+std") null model predictions in cell lines used in this study. Colours and annotations are the same as in (b). Source data are provided as a Source Data file.

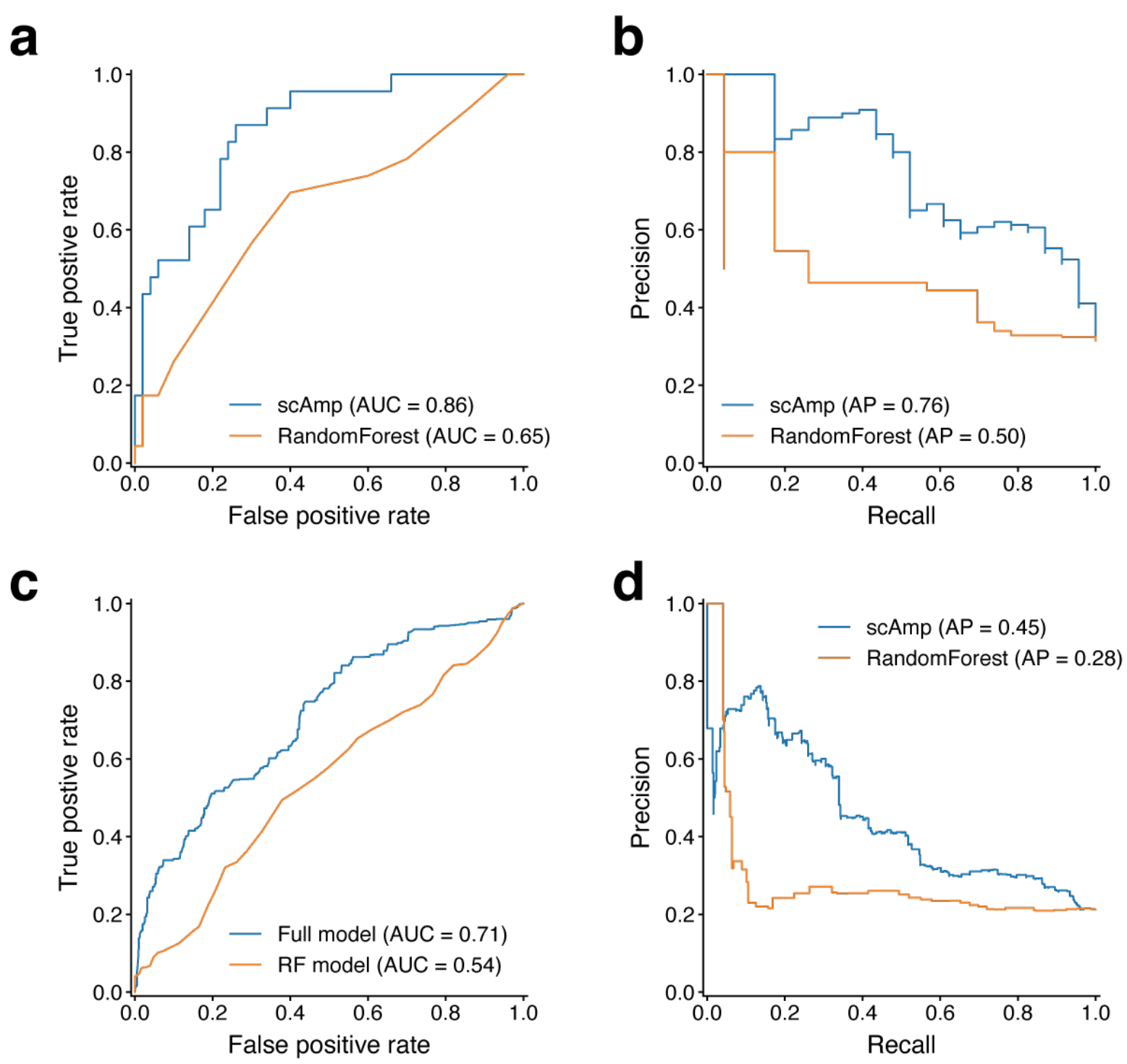

Supplementary Figure 5. Concordance of scAmp 1.0.0 model and RandomForest classifier with bulk WGS classification in patient tumour data. Receiver-operator characteristic (ROC) and precision-recall curves (PRC) measuring concordance in held-out patient data from The Cancer Genome Atlas (TCGA) between bulk whole genome sequencing (WGS)-based calls and single-cell-based calls (*scAmp* and RandomForest). **(a-b)** ROC (a) and PRC (b) of models on the per-sample prediction task (predicting whether a given sample has at least one ecDNA+ gene amplification, measured against bulk WGS predictions). (**c-d)** ROC (c) and PRC (d) of models on the per-gene prediction task (predicting whether a given gene is amplified on ecDNA in a given tumour sample, measured against bulk WGS predictions). AUROC and AUPRCs are reported for each model. All labels for assessing performance are derived from bulk WGS analysis, and do not represent ground truth. AUROC = Area Under ROC; AUPRC = Area Under PRC

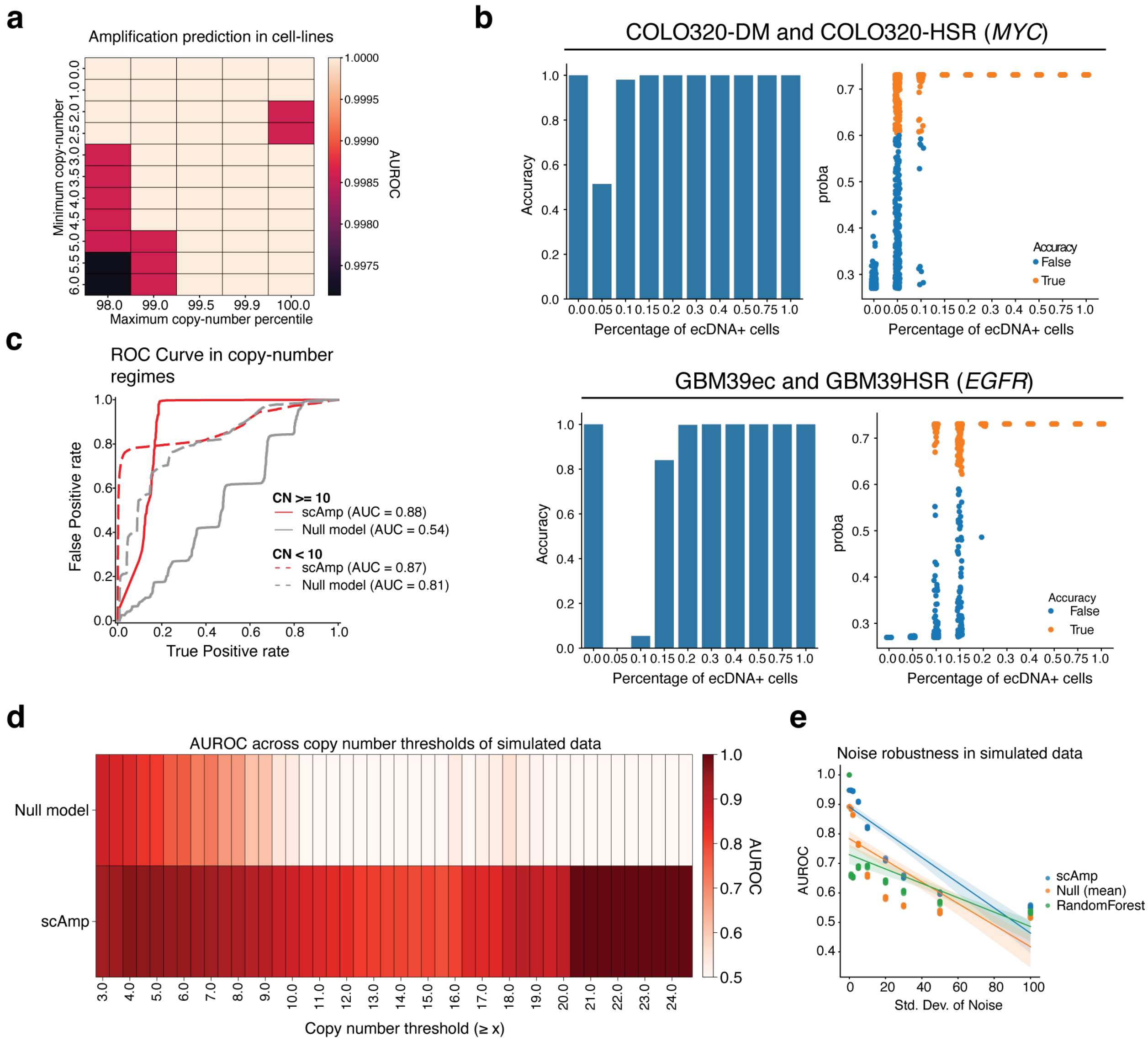

Supplementary Figure 6. Additional benchmarking of model predictions on realistic tumour settings. (a) *scAmp* model performance, measured by Area Under Receiver Operator Characteristic (AUROC), on predicting oncogene amplification status in cell lines using various data preprocessing strategies. **(b)** Performance of *scAmp* model on synthetic mixtures of ecDNA+ and HSR+ cell lines (*MYC* in COLO320-DM/HSR and *EGFR* GBM39ec/HSR, top and bottom respectively). Model accuracy (left) and probability of amplification (right) are shown for varying amounts of ecDNA+ fraction. **(c)** ROC curve of *scAmp* and mean-only null model on simulated amplicons with average copy-number greater than, or less than, 10. AUROCs are reported in the legend. **(d)** Summary AUROCs of *scAmp* and null model on simulated amplicons subset by a minimum copy-number

threshold. **(e)** Performances (AUROC) for *scAmp*, mean-only null model, and Random Forest model on simulated data with increasing amounts of noise added to input feature matrix (noise modelled by a mean-centred Gaussian distribution with increasing standard deviation). ecDNA = extrachromosomal DNA, HSR = homogeneously-staining regions. Source data are provided as a Source Data file.

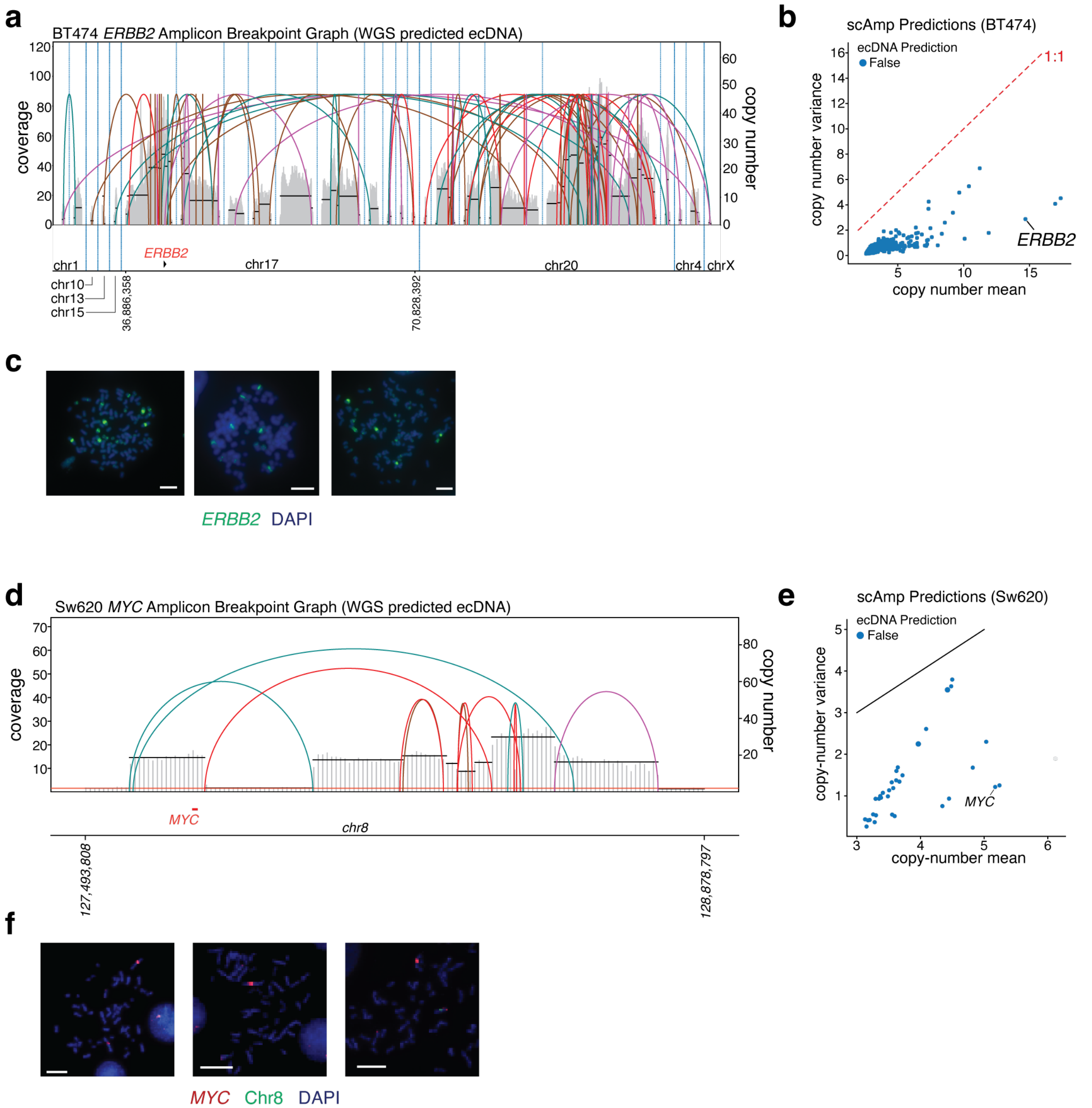

Supplementary Figure 7. Analysis of amplification status in the BT474 and Sw620 cell lines. **(a)** A breakpoint graph of the *ERBB2* amplicon in BT474 predicted by AmpliconArchitect[10] analysis of whole genome sequencing (WGS) data as extrachromosomal DNA+ (ecDNA+) (obtained from AmpliconRepository). The coverage over the amplified intervals is shown as a grey distribution, and each arc represents a breakpoint inferred from AmpliconArchitect. Only the *ERBB2* gene locus is shown underneath for clarity. **(b)** Scatterplots summarizing the mean and variance of gene copy-number distributions for each gene in the BT474 cell line, along with predictions from

scAmp. *ERBB2* is predicted to be chromosomally amplified. **(c)** Metaphase spread images of cells with DNA FISH for *ERBB2*; here, *ERBB2* co-localizes with DAPI+ chromosomal signal and is inferred to be chromosomally integrated in BT474. Scale bars indicate 10μm. **(d)** A breakpoint graph of the *MYC* amplicon in Sw620 predicted by AmpliconArchitect[32] analysis of WGS data as ecDNA+ (obtained from AmpliconRepository). The coverage over the amplified intervals is shown as a grey distribution, and each arc represents a breakpoint inferred from AmpliconArchitect. Only the *MYC* gene locus is shown underneath for clarity. **(e)** Scatterplots summarizing the mean and variance of gene copy-number distributions for each gene in the Sw620 cell line, along with predictions from *scAmp*. *MYC* is predicted to be chromosomally amplified. **(f)** Metaphase spread images of cells with DNA FISH for *MYC* and centromere 8 probe. *MYC* co-localizes with DAPI+ chromosomal signal and is inferred to be chromosomally integrated in Sw620. Scale bars indicate 10μm.

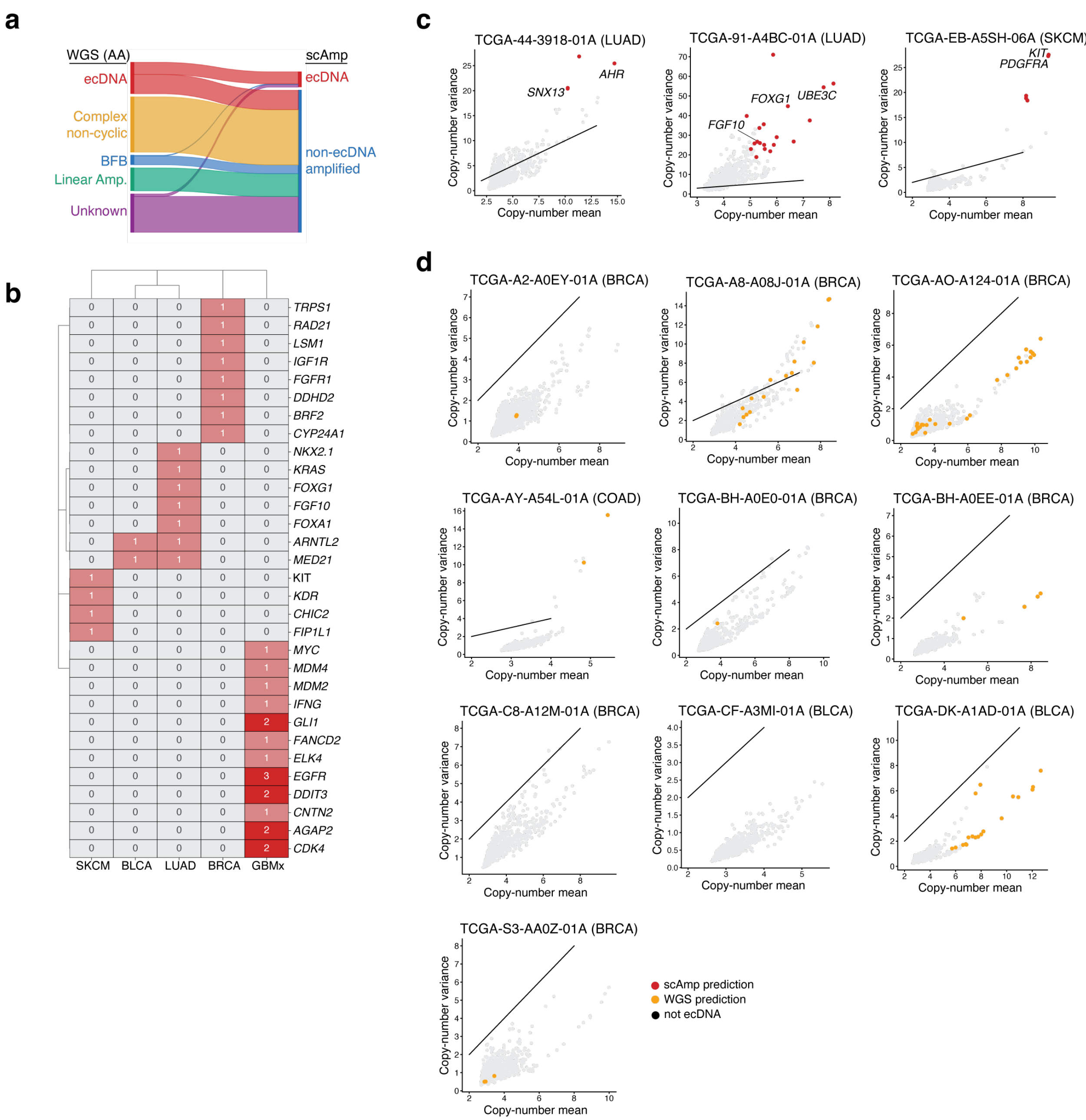

Supplementary Figure 8. *scAmp* predictions on TCGA scATAC-seq data. (a) Comparisons of whole genome sequencing (WGS) amplicon predictions versus *scAmp* predictions in The Cancer Genome Atlas (TCGA) cohort profiled with single-cell assay for transposase-accessible chromatin by sequencing (scATAC-seq). *scAmp* only distinguishes between extrachromosomal DNA (ecDNA) and chromosomal amplifications. Most discrepancies are with WGS-predicted ecDNA amplicons being predicted to be chromosomally amplified by *scAmp*, consistent with analysis of the BT474 cell line. **(b)** Summary of genes most frequently predicted to be amplified on ecDNA by

scAmp by tumour type. Rows and columns are clustered. **(c-d)** Summary of per-sample discrepancies between *scAmp* and WGS analysis. Scatterplots summarize the mean and variance of gene copy-number distributions for each gene in each patient tumour. Red colours in (c) indicate genes that are predicted by *scAmp* to be ecDNA-amplified, though predicted to be chromosomally amplified by WGS. Orange colours in (d) indicate genes that are predicted by WGS to be ecDNA amplified, though predicted to be chromosomally amplified by *scAmp*. Diagonal lines indicate the 1:1 line. Focal genes of potential interest are called out in (c). Source data are provided as a Source Data file.

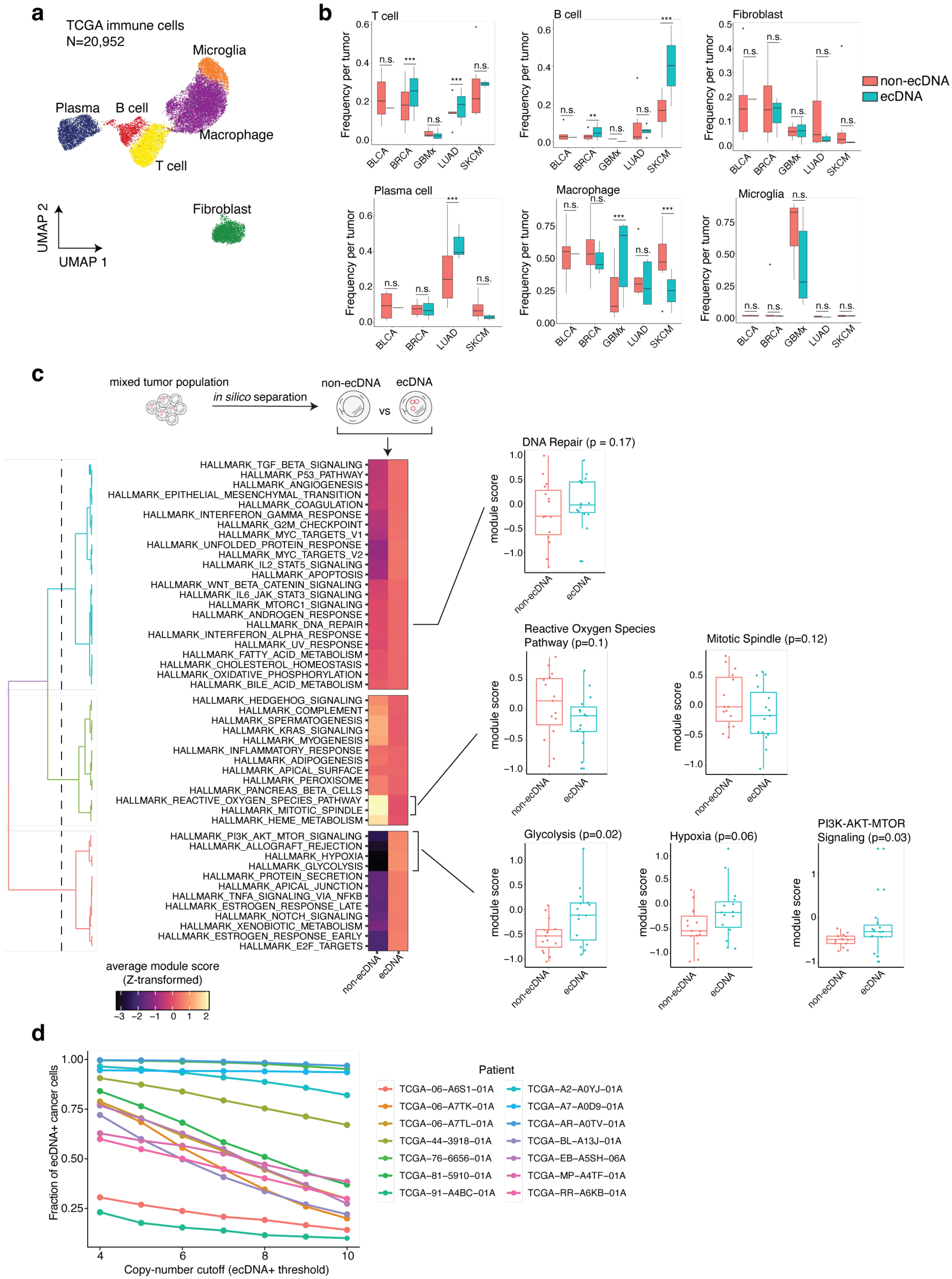
a
TCGA immune cells
N=20,952
Microglia
Plasma
B cell
Macrophage
T cell
Fibroblast
UMAP 2
UMAP 1
b
T cell
B cell
Fibroblast
Plasma cell
Macrophage
Microglia
Frequency per tumor
BLCA
BRCA
GBMx
LUAD
SKCM
n.s.
non-ecDNA
ecDNA
c
mixed tumor population
in silico separation
non-ecDNA
vs
ecDNA
HALLMARK_TGF_BETA_SIGNALING
HALLMARK_P53_PATHWAY
HALLMARK_ANGIOGENESIS
HALLMARK_EPITHELIAL_MESENCHYMAL_TRANSITION
HALLMARK_COAGULATION
HALLMARK_INTERFERON_GAMMA_RESPONSE
HALLMARK_G2M_CHECKPOINT
HALLMARK_MYC_TARGETS_V1
HALLMARK_UNFOLDED_PROTEIN_RESPONSE
HALLMARK_MYC_TARGETS_V2
HALLMARK_IL2_STAT5_SIGNALING
HALLMARK_APOPTOSIS
HALLMARK_WNT_BETA_CATENIN_SIGNALING
HALLMARK_IL6_JAK_STAT3_SIGNALING
HALLMARK_MTORC1_SIGNALING
HALLMARK_ANDROGEN_RESPONSE
HALLMARK_DNA_REPAIR
HALLMARK_INTERFERON_ALPHA_RESPONSE
HALLMARK_UV_RESPONSE
HALLMARK_FATTY_ACID_METABOLISM
HALLMARK_CHOLESTEROL_HOMEOSTASIS
HALLMARK_OXIDATIVE_PHOSPHORYLATION
HALLMARK_BILE_ACID_METABOLISM
HALLMARK_HEDGEHOG_SIGNALING
HALLMARK_COMPLEMENT
HALLMARK_SPERMATOGENESIS
HALLMARK_KRAS_SIGNALING
HALLMARK_MYOGENESIS
HALLMARK_INFLAMMATORY_RESPONSE
HALLMARK_ADIPOGENESIS
HALLMARK_APICAL_SURFACE
HALLMARK_PEROXISOME
HALLMARK_PANCREAS_BETA_CELLS
HALLMARK_REACTIVE_OXYGEN_SPECIES_PATHWAY
HALLMARK_MITOTIC_SPINDLE
HALLMARK_HEME_METABOLISM
HALLMARK_PI3K_AKT_MTOR_SIGNALING
HALLMARK_ALLOGRAFT_REJECTION
HALLMARK_HYPOXIA
HALLMARK_GLYCOLYSIS
HALLMARK_PROTEIN_SECRETION
HALLMARK_APICAL_JUNCTION
HALLMARK_TNFA_SIGNALING_VIA_NFKB
HALLMARK_ESTROGEN_RESPONSE_LATE
HALLMARK_NOTCH_SIGNALING
HALLMARK_XENOBIOTIC_METABOLISM
HALLMARK_ESTROGEN_RESPONSE_EARLY
HALLMARK_E2F_TARGETS
average module score
(Z-transformed)
DNA Repair (p = 0.17)
Reactive Oxygen Species Pathway (p=0.1)
Mitotic Spindle (p=0.12)
Glycolysis (p=0.02)
Hypoxia (p=0.06)
PI3K-AKT-MTOR Signaling (p=0.03)
module score
d
Fraction of ecDNA+ cancer cells
Copy-number cutoff (ecDNA+ threshold)
Patient
TCGA−06−A6S1−01A
TCGA−06−A7TK−01A
TCGA−06−A7TL−01A
TCGA−44−3918−01A
TCGA−76−6656−01A
TCGA−81−5910−01A
TCGA−91−A4BC−01A
TCGA−A2−A0YJ−01A
TCGA−A7−A0D9−01A
TCGA−AR−A0TV−01A
TCGA−BL−A13J−01A
TCGA−EB−A5SH−06A
TCGA−MP−A4TF−01A
TCGA−RR−A6KB−01A

Supplementary Figure 9. ecDNA-centric analysis of TCGA scATAC-seq data. **(a-b)** Analysis of immune and stromal compartment of The Cancer Genome Atlas (TCGA) tumors. **(a)** Uniform Manifold Approximation and Projection (UMAP) plot of single-cell chromatin accessibility profiles of immune and stromal cells. Major cell types are annotated. **(b)** Distribution of cell type frequencies in extrachromosomal DNA+ (ecDNA+) and non-ecDNA tumours. Frequencies are normalized to the total number of immune and stromal cells profiled in a given tumour (cancer cells excluded). Significances, corresponding to empirical two-sided Wilcoxon rank-sums-test *p*-values from 100 random shuffles, are indicated above individual boxplots (***: $p \leq 0.01$; **: $p \leq 0.05$). **(c)** Analysis of ecDNA-specific effects on cancer cells. ecDNA+ cancer cells were identified *in silico* based on the copy-number of genes predicted to be on ecDNA (**Methods**). Module scores for Hallmark signature sets were assessed in ecDNA+ and non-ecDNA cells, and average Z-transformed scores are displayed in a clustered heatmap (left). Distributions of individual gene sets are shown in boxplots (right). Each point in the boxplot is the mean module score for the cancer cells in a patient. Boxplots report the quartiles of the distributions (center corresponds to median value, boundaries are the 25th and 75th percentiles), and whiskers extend to 1.5x the interquartile distance. P-values are computed using a one-sided Wilcoxon rank-sums test. **(d)** Fraction of ecDNA+ cancer cells detected in each ecDNA+ tumour with varying copy-number thresholds. Each line indicates a different ecDNA+ tumour. Source data are provided as a Source Data file.
