# Supplementary material for: scAmp enables focal gene amplification analysis from single-cell data
Source: Nat Commun. 2026 Aug 8;17:9563. doi: 10.1038/s41467-026-76417-3 (PMC13550473; doi:10.1038/s41467-026-76417-3)
Supplement: Supplementary file 2 [file 41467_2026_76417_MOESM2_ESM.pdf]

nature portfolio

Corresponding author(s): Chang, Mischel

Last updated by author(s): 6/02/2026

Reporting Summary

Nature Portfolio wishes to improve the reproducibility of the work that we publish. This form provides structure for consistency and transparency in reporting. For further information on Nature Portfolio policies, see our Editorial Policies and the Editorial Policy Checklist.

Statistics

For all statistical analyses, confirm that the following items are present in the figure legend, table legend, main text, or Methods section.

n/a	Confirmed	
☐	☒	The exact sample size (*n*) for each experimental group/condition, given as a discrete number and unit of measurement
☒	☐	A statement on whether measurements were taken from distinct samples or whether the same sample was measured repeatedly
☐	☒	The statistical test(s) used AND whether they are one- or two-sided<br>*Only common tests should be described solely by name; describe more complex techniques in the Methods section.*
☒	☐	A description of all covariates tested
☒	☐	A description of any assumptions or corrections, such as tests of normality and adjustment for multiple comparisons
☐	☒	A full description of the statistical parameters including central tendency (e.g. means) or other basic estimates (e.g. regression coefficient) AND variation (e.g. standard deviation) or associated estimates of uncertainty (e.g. confidence intervals)
☐	☒	For null hypothesis testing, the test statistic (e.g. *F*, *t*, *r*) with confidence intervals, effect sizes, degrees of freedom and *P* value noted<br>*Give P values as exact values whenever suitable.*
☒	☐	For Bayesian analysis, information on the choice of priors and Markov chain Monte Carlo settings
☒	☐	For hierarchical and complex designs, identification of the appropriate level for tests and full reporting of outcomes
☐	☒	Estimates of effect sizes (e.g. Cohen's *d*, Pearson's *r*), indicating how they were calculated

Our web collection on statistics for biologists contains articles on many of the points above.

Software and code

Policy information about availability of computer code

Data collection

Paired scATAC-seq and scRNA-seq library generation
Single-cell paired RNA-seq and ATAC-seq libraries were generated with the 10X Chromium Single-Cell Multiome ATAC + Gene Expression kit according to the manufacturer's protocol. Libraries were sequenced on an Illumina NovaSeq 6000 system.

Data analysis

CODE AVAILABILITY
The scAmp software used to develop the model and generate these results is publicly available and has been deposited on Gtihub at https://github.com/JonesCompBioLab/scamp under the MIT License. The specific version of the code associated with this publication si archived in Zenodo and accessible via 10.5281/zenodo.2043484842.

Copy-number analysis of scATAC-seq data
We calculated copy-numbers in 3Mb windows based on the background ATAC-seq signal as we previously described and validated14,20,29. Briefly, we determined read counts in large intervals across the genome using a sliding window of 3Mb moving in 1Mb increments across the reference genome (hg19). Genomic regions with known mapping artifacts were filtered out using the ENCODE hg19 blacklist. For each interval, insertions per bp were calculated and compared to 200 of its nearest neighbours with matched GC nucleotide content. The mean log2[fold change] was computed for each interval. Based on a diploid genome, copy numbers were calculated using the formula CN=2 ×2^log_2[FC] where CN denotes copy number and FC denotes mean fold change compared to neighbouring intervals. To query the copy numbers of a gene, we obtained all genomic intervals that overlapped with the annotated gene sequence and computed the mean copy numbers of those intervals.

Analysis of scATAC-seq data from cell lines

Single-cell Multiome data was aligned using cellranger-arc count (10X Genomics, v. 1.0.0); for BT474, we aligned directly to the hg19 genome; for H2170, Sw620, GBM39ec, and GBM39HSR, we lifted over fragments aligned to hg38 to hg19 using the UCSC hg38tohg19 chain (hg38ToHg19.over.chain.gz) in order to integrate this data with our existing cell-line atlas. For the purposes of this study, we focused on the scATAC-seq fraction of the data and performed analysis using ArchR35 (v. 1.0.2). Cells with fewer than 1000 unique fragments or TSS enrichment less than 4 were filtered out. Doublets were removed using ArchR's addDoubletScores and filterDoublets commands with the following parameters: k=10, knnMethod="UMAP", LSIMethod=1. Dimensionality reduction was performed using Iterative Latent Semantic Indexing with the addIterativeLSI function. For each cell line, we converted single-cell copy-number distributions of amplified genes (those with average copy-number exceeding 2.5) to scAmp-compatible features with the prepare_copy_numbers function with the following parameters: min_copy_number=3, max_percentile=99.9. Genes with predicted likelihood exceeding 0.6 were marked as ecDNA+.

Predicting gene amplification status from TCGA scATAC-seq data
Copy-numbers were computed as described above (see section entitled "Copy-number analysis of scATAC-seq data").For each tumor sample, we compared the average copy-numbers of genes derived from scATAC-seq data to the copy-numbers inferred from AmpliconArchitect analysis on bulk WGS data and find correlations above 0.5 (Extended Data Figure 3c). For each tumor, we converted single-cell copy-number distributions of amplified genes (those with average copy-number exceeding 2.5) to scAmp-compatible features with the prepare_copy_numbers function with the following parameters: min_copy_number=3, max_percentile=99.9. Genes with predicted likelihood exceeding 0.6 were marked as ecDNA+.
To quantify the frequency of ecDNA+ cells in Figure 3c, we computed the fraction of cells that amplified a gene predicted to be on ecDNA at 4 copies or more. We report the stability of these quantifications in Extended Data Figure 9d. To assess the phenotypic consequences of ecDNA on cancer cells, we first computed module scores for each signature in the MSigDB36 Hallmark set (available at https://www.gsea-msigdb.org/gsea/msigdb/human/genesets.jsp?collection=H) with ArchR (version v. 1.0.2) using the addModuleScore function with useMatrix='GeneScoreMatrix'. We then performed in silico separation of cancer cells based on whether they amplified any gene predicted to be on ecDNA at 4 copies or more and assessed differential signature scores based on this in silico separation.
We also analysed the shifts in immune and stromal composition based on the ecDNA status of a given tumour (Extended Data Figure 9a-b). To do this, we separated immune and stromal populations from epithelial populations and annotated cells as previously described24.

Analysis of GBMx sample TCGA–06–A7TK–01A
To analyse the subclonal evolution of the GBMx sample TCGA–06–A7TK–01A, we first inspected the shared patterns of copy-number amplifications in cancer cells computed across 3Mb windows (see above "Copy-number analysis of scATAC-seq data"). To identify regions that were likely ecDNA+, we focused on 3Mb regions that were amplified at greater than 7 copies in at least 5% of cells. We then performed hierarchical clustering of cells based on these regions using Euclidean distance and "ward" linkage and extracted 4 clusters based on tree depth.
To create the single-cell UMAP projection of this patient's data, we performed analysis with ArchR (version 1.0.2). We first performed dimensionality reduction using Iterative Latent Semantic Indexing with the addIterativeLSI function with the following parameters: useMatrix='TileMatrix', iterations=2, clusterParams=list(resolution=c(0.2, 0.4), n.start=10), and varFeatures=30000). We computed a UMAP projection from the LSI dimensions using nNeighbors=15, minDist=0.2, and metric='cosine'. Genome tracks were visualized using the plotBrowserTrack function using tileSize=100, upstream=1000000, and downstream = 1500000.

For manuscripts utilizing custom algorithms or software that are central to the research but not yet described in published literature, software must be made available to editors and reviewers. We strongly encourage code deposition in a community repository (e.g. GitHub). See the Nature Portfolio guidelines for submitting code & software for further information.

Data

Policy information about availability of data

All manuscripts must include a data availability statement. This statement should provide the following information, where applicable:

- Accession codes, unique identifiers, or web links for publicly available datasets
- A description of any restrictions on data availability
- For clinical datasets or third party data, please ensure that the statement adheres to our policy

Paired scATAC-seq and scRNA-seq library generation
Single-cell paired RNA-seq and ATAC-seq libraries were generated with the 10X Chromium Single-Cell Multiome ATAC + Gene Expression kit according to the manufacturer's protocol. Libraries were sequenced on an Illumina NovaSeq 6000 system. Single cell Multiome data for other cell lines were obtained from the following public databases: COLO320-DM & COLO320-HSR (GEO accession GSE159986), GBM39-KT (SRA accension SRS21730888), SNU16 (SRA accension SRS21730887), SNU16m1 (SRA accension SRS21730902), Sw620 (Genome Sequence Archive accession PRJCA021248) and TR14 (SRA accension SRS21730907).
Single-cell ATAC-seq data for patients from TCGA was obtained from NCI Genomic Data Commons (https://gdc.cancer.gov/about-data/publications/TCGA-ATAC-Seq-2024).

Cell culture
The BT-474 cell line was obtained from American Type Culture Collection (ATCC) [Cat# CRL-3247] and was cultured in standard culture media: RPMI 1640 (Gibco, A1049101), 10% fetal bovine serum (FBS; Gibco A5670401), 1% penicillin/streptomycin (Thermo Fisher Scientific, 15140-122), 1% HEPES (Thermo Fisher Scientific, 15630080), 1% Glutamax (Thermo Fisher Scientific, 35050061) at 37°C in 5% CO2. The H2170 cell-line was obtained from American Type Culture Collection [Cat# CRL-5928] and was cultured in Dulbecco's modified Eagle's medium (DMEM; Thermo Fisher Scientific, 11995073), 10% FBS and 1% penicillin–streptomycin at 37°C in 5% CO2. TR14 cells were a kind gift from J.H. Shulte and were cultured in RPMI 1640 + 10% FBS and 1% PSQ.

DATA AVAILABILITY
The raw single-cell multiome sequencing data generated in this study have been deposited in the NCBI SRA database under BioProject accession code PRJNA1472115 (https://www.ncbi.nlm.nih.gov/bioproject/PRJNA1472115). The source imaging data has been deposited in the Stanford Digital Repository40 (https://doi.org/10.25740/bx954rw4074). Other source data are provided with this paper. AmpliconClassifier outputs containing ecDNA coordinates in TCGA samples were previously published and are publicly available at figshare (https://doi.org/10.6084/m9.figshare.24768555.v1). Previously published scATAC-seq data of cell lines were obtained from public repositories: GEO Series GSE159986 (COLO320DM and COLO320HSR), SRA accension SRS21730888 (GBM39KT), SRA accensions SRS21730887 and SRS21730902 (SNU16 and SNU16m1, respectively), SRA accension SRS21730907 (TR14), and Genome Sequence Archive PRJCA021248 (Sw620). Previously published TCGA scATAC-seq data are publicly available via NCI Genomic Data Commons (https://gdc.cancer.gov/about-data/publications/TCGA-

ATAC-Seq-2024). Previously published metaphase FISH images are available at https://doi.org/10.25740/cp783mb3217 (COLO320DM and COLO320 HSR), https://doi.org/10.25740/ff315yn8920 (SNU16, GBM39KT, TR14) and https://figshare.com/s/ab6a214738aa43833391 (Sw620).

Research involving human participants, their data, or biological material

Policy information about studies with human participants or human data. See also policy information about sex, gender (identity/presentation), and sexual orientation and race, ethnicity and racism.

Reporting on sex and gender	N/A
Reporting on race, ethnicity, or other socially relevant groupings	N/A
Population characteristics	N/A
Recruitment	N/A
Ethics oversight	Tumors with amplified oncogenes were identified from the Stanford Pathology archive in accordance with IRB protocol #69198 which was approved with the Stanford University Institutional Review Board.

Note that full information on the approval of the study protocol must also be provided in the manuscript.

Field-specific reporting

Please select the one below that is the best fit for your research. If you are not sure, read the appropriate sections before making your selection.

☒ Life sciences ☐ Behavioural & social sciences ☐ Ecological, evolutionary & environmental sciences

For a reference copy of the document with all sections, see nature.com/documents/nr-reporting-summary-flat.pdf

Life sciences study design

All studies must disclose on these points even when the disclosure is negative.

Sample size	No sample size calculation was performed. In the case of single-cell sequencing, we aimed to sequence thousands of cells per cell line, as is supported by a single reaction. For sequencing studies, we sequenced DNA from at least 1,000,000 cells which captures much of the genetic heterogeneity in a cancer population.
Data exclusions	Single-cell ATAC analysis: Cells with fewer than 1000 unique fragments or TSS enrichment less than 4 were filtered out. Doublets were removed using ArchR's addDoubletScores and filterDoublets commands with the following parameters: k=10, knnMethod="UMAP", LSIMethod=1.
Replication	Computational experiments were repeated hundreds of times to assess robustness. Thousands of cells were sequenced in single-cell experiments. In imaging experiments, quantification was performed across hundreds of cells and across at least 2 fields of view.
Randomization	No randomization was used for cell lines or mouse experiments as we were not testing the effects of perturbations. As Investigators were not blinded to allocation during experiments and outcome assessment, randomization was not relevant to this study.
Blinding	All data were collected using instruments without bias. Because these data were generated using objective quantifications, researchers assessing results were not blinded for the experimental design. Blinding is not relevant to this study.

Reporting for specific materials, systems and methods

We require information from authors about some types of materials, experimental systems and methods used in many studies. Here, indicate whether each material, system or method listed is relevant to your study. If you are not sure if a list item applies to your research, read the appropriate section before selecting a response.

Materials & experimental systems

n/a	Involved in the study	
☒	☐	Antibodies
☐	☒	Eukaryotic cell lines
☒	☐	Palaeontology and archaeology
☐	☒	Animals and other organisms
☒	☐	Clinical data
☒	☐	Dual use research of concern
☒	☐	Plants

Methods

n/a	Involved in the study	
☒	☐	ChIP-seq
☒	☐	Flow cytometry
☒	☐	MRI-based neuroimaging

Eukaryotic cell lines

Policy information about cell lines and Sex and Gender in Research

Cell line source(s)	Parental SNU16, COLO320-DM, COLO320-HSR, BT474, and H2170 were obtained from ATCC. The TR14 neuroblastoma cell line was a gift from J. J. Molenaar (Princess Máxima Center for Pediatric Oncology, Utrecht, Netherlands). The GBM39 cell line was derived from a patient with glioblastoma undergoing surgery at Mayo Clinic, Rochester, Minnesota as described previously (PMID: 16609043). The monoclonal SNU16m1 was a subline of the parental SNU16 cells generated from a single cell after lentiviral transduction and stable expression of dCas9-KRAB as we previously described (PMID: 34819668).
Authentication	Cell lines obtained from ATCC were not authenticated. TR-14 cell line identity for the master stock was verified by STR genotyping (IDEXX BioResearch, Westbrook, ME).
Mycoplasma contamination	Cells tested negative for mycoplasma.
Commonly misidentified lines (See ICLAC register)	None of the cell lines used are registered by ICLAC as commonly misidentified.

Animals and other research organisms

Policy information about studies involving animals; ARRIVE guidelines recommended for reporting animal research, and Sex and Gender in Research

Laboratory animals	Female Foxn1nu Athymic Nude mice (Charles River Laboratories) were housed under standard conditions.
Wild animals	N/A
Reporting on sex	Female mice were used.
Field-collected samples	N/A
Ethics oversight	Mice were maintained at Stanford under standard regulatory conditions. The study received ethical approval from Stanford APLAC/ IACUC Protocol #: 34041.

Note that full information on the approval of the study protocol must also be provided in the manuscript.

Plants

Seed stocks	*Report on the source of all seed stocks or other plant material used. If applicable, state the seed stock centre and catalogue number. If plant specimens were collected from the field, describe the collection location, date and sampling procedures.*
Novel plant genotypes	*Describe the methods by which all novel plant genotypes were produced. This includes those generated by transgenic approaches, gene editing, chemical/radiation-based mutagenesis and hybridization. For transgenic lines, describe the transformation method, the number of independent lines analyzed and the generation upon which experiments were performed. For gene-edited lines, describe the editor used, the endogenous sequence targeted for editing, the targeting guide RNA sequence (if applicable) and how the editor was applied.*
Authentication	*Describe any authentication procedures for each seed stock used or novel genotype generated. Describe any experiments used to assess the effect of a mutation and, where applicable, how potential secondary effects (e.g. second site T-DNA insertions, mosiacism, off-target gene editing) were examined.*
