# Supplementary material for: scAmp enables focal gene amplification analysis from single-cell data
Source: Nat Commun. 2026 Aug 8;17:9563. doi: 10.1038/s41467-026-76417-3 (PMC13550473; doi:10.1038/s41467-026-76417-3)
Supplement: Supplementary file 3 [file 41467_2026_76417_MOESM3_ESM.pdf]

nature portfolio

Peer Review File

scAmp enables focal gene amplification analysis from single-cell data

Corresponding Author: Professor Howard Chang

Parts of this Peer Review File have been redacted as indicated to remove third-party material.

This file contains all reviewer reports in order by version, followed by all author rebuttals in order by version.

Version 0:

Reviewer comments:

Reviewer #1

(Remarks to the Author)
In the manuscript:"scAmp analyzes focal gene amplifications at single cell resolution", under consideration in Nature Coms, Jones et al. make use of the high single cell variation of extrachromosomal amplified DNA to devise an algorithm for ecDNA identification using single cell ATTAC-seq data. The method is timely and of importance to the cancer field in general. As such the manuscript will be of high interest. I have a few comments to help improve the presentation of the results the authors may want to consider prior to publication.

The readability of the manuscript would benefit from a better structuring of the results section. Also, the discussion seems extremely short and the authors could extend a little.

The authors implement a neural network architecture to identify tumours with extrachromosomal DNA. This seems to work very well, but feels a bit like shooting with canons on sparrows. Not suggesting changing the analysis here, but out of curiosity, wouldn't grouping by a single number like varianz/mean (high vs. low) suffice to distinguish cases?

The authors say they can estimate the fraction of ecDNA carrying cells in the sample. There are two questions, (i) the authors use a cutoff of at least 4 ecDNA copies per cell. However, if I understood correctly, ecDNA copy number heterogeneity also allows for cells with 1 to 3 copies, thus this cutoff will underestimate the fraction? (ii) Does the tumour microenvironment bias these results? For example, a sample could contain a high fraction of immune infiltrating cells that bias estimates?

The authors say their method can help timing the occurrence of ecDNAs, but somehow the discussion lacks details. I assume you mean to say that a high fraction of ecDNA+ cells suggest early arrival? Again this might be biased by tumour microenvironment and/or effects of spatial sampling. I think in this context it will also be useful to give exposure to the works of Okonechnikov et al.Nature 2025 and Noorani et al Cancer Discovery 2025.

The authors specifically discuss that they found ERBB2 in a breast cancer cell line to be chromosomally amplified and not on ecDNA. This is interesting and potentially important, especially as it has been suggested recently that a high proportion of Her2+ Breast Cancers carry ecDNAs (Houlahan et al. Nature 2025). I appreciate, the example in the manuscript here is n=1 which does not allow to generalize. Is there a possibility to acquire other sample or data. Do the observations here have possible implications for the interpretation of the results in Houlahan et al.?

(Remarks on code availability)

Reviewer #2

(Remarks to the Author)
Matthew Jones and colleagues in their manuscript present scAmp ("single-cell amplicon" analysis), a probabilistic algorithm for detecting and analyzing extrachromosomal DNA (ecDNA) from single-cell datasets.
While genomic alterations are well known and highly studied hallmark of cancer, ecDNA has more recently emerged as a

critical mechanism promoting oncogene amplification, tumor progression, and resistance to therapy.
Despite its importance, the mechanisms by which ecDNA contributes to intratumor heterogeneity and clonal selection are largely unknown. New methodological approaches to analyse ecDNA could therefore provide critical insight and advance our understanding of these fundamental processes.

Leveraging machine learning approaches, using copy-number distributions data and exploiting one of the major features of ecDNA biology – the non-Mendelian inheritance of individual ecDNA molecules during cell division – the authors formulated a model which was able to correctly predict the likelihood of genes being amplified in ecDNA from those deriving from chromosomal amplification, in single-cell assays, such as of scWGS or scATAC-seq. They successfully validate their model in tumor samples from The Cancer Genome Atlas (TCGA) with paired WGS data.
Applying scAmp to single-cell ATAC-seq experiments they were also able to perform phenotypic analyses of cell states within ecDNA+ tumors, which provided the opportunity to infer the subclonal heterogeneity of amplified oncogenes in ecDNA within tumors.

In general, the methodological approach is clearly described and logically structured. The authors introduce the method in a stepwise manner that allows the reader to understand both its conceptual motivation and its practical implementation. Key components of the workflow are well illustrated, and they clarify the underlying logic without unnecessary complexity. The writing is precise and focused, which makes the technically sophisticated method accessible to a broad readership.
The two main figures and the Extended Data Figures are presented with a high level of completeness and precision. They are carefully designed, clearly annotated, and effectively support the main text and provide valuable methodological and technical detail that facilitates interpretation of the results and enhances transparency. Overall, the quality and organization of the visual material significantly strengthen the manuscript. The validation in patient tumors suggests that the method presented it is solid and potentially broadly useful.

That said, given that the primary contribution is methodological, the manuscript would benefit from a more explicit statement of the methodological novelty. In particular, the authors should clearly articulate which specific limitations of existing approaches are addressed by the proposed method, and in what sense these capabilities are not currently available.

I note that a very recent study published during the review process (*) appears to address a closely related objective. While this does not diminish the value of the present work, it would be probably important to acknowledge this study and to clarify the relationship between the two approaches, highlighting similarities and differences in scope, implementation, and applicability.
(*) https://doi.org/10.1186/s13059-026-03933-2

Overall, I am positive about the manuscript, and I believe it will be suitable for publication after the last suggested very minor revisions.

(Remarks on code availability)

Reviewer #3

(Remarks to the Author)
Review for

scAmp analyzes focal gene amplifications at single cell resolution

Summary

This manuscript introduces scAmp, a method designed to infer whether focal gene amplifications are carried on extrachromosomal DNA (ecDNA) versus chromosomal amplification states such as homogeneous staining regions (HSRs) using single cell datasets. The core idea is that ecDNA's non Mendelian inheritance can increase cell to cell copy number variability, and that summary features of gene level copy number distributions across cells can be used to classify amplification mode. The authors train a supervised classifier largely on simulated copy number distributions, apply it to selected cell lines and TCGA scATAC seq tumor datasets, and present downstream analyses of heterogeneity and chromatin accessibility.

The biological question is important and the conceptual approach is interesting. At the same time, several aspects of the current training, benchmarking, validation, and framing limit how confidently the central claims can be interpreted. In my view, the manuscript would benefit from revisions that sharpen what is and is not being inferred at the gene versus cell level, strengthen independent validation, more explicitly address assay dependent limitations of scATAC derived copy number estimates, and include standard sensitivity and uncertainty analyses expected for ML based methods. In particular, robust differentiation of ecDNA versus chromosomal amplification across tumors and across assays is not established with the current training and validation strategy. I recommend major revision. If the validation additions below are not feasible, the manuscript should be reframed as a proof of concept in a limited set of canonical models and the language should be scaled accordingly. I would consider the structure anchored validation panel and the cross modality check to be required for supporting the headline claim of robust ecDNA versus chromosomal amplification discrimination in tumors.

Major concerns
1. Benchmarking and ground truth for tumors are not independent

What the authors show
For tumors, scAmp is evaluated against WGS based calls such as AmpliconSuite or AmpliconClassifier, and the manuscript also discusses cases in which bulk WGS may misclassify ecDNA versus integrated or ancestral ecDNA derived amplification states.

Why this is a concern
If the comparison labels are derived from the same class of bulk WGS approaches whose limitations are discussed, then performance metrics in tumors are best interpreted as concordance with WGS derived labels, rather than definitive accuracy. The BT474 example is useful in highlighting that WGS based labels can be ambiguous, but it also underscores the need for independent truth sets when reporting accuracy like metrics.

Revisions suggested

Adjust wording in the Abstract and Results so tumor benchmarking is described as concordance with WGS based calls rather than accuracy, unless orthogonal truth is provided.

Add a short subsection stating clearly that tumor labels are WGS derived and that these labels are not definitive for ecDNA versus integrated ecDNA like structures, so AUROCs in tumors should be interpreted accordingly.

Provide at least one independent validation setting where ecDNA versus chromosomal amplification is established by a structure resolving approach rather than WGS calling alone.

2. Single cell resolution language is likely to be misunderstood

What the authors show
scAmp featurizes each gene using distribution statistics computed across cells, then classifies genes as ecDNA associated or chromosomally amplified. The manuscript then defines ecDNA positive cells using thresholds applied to genes predicted to be ecDNA associated.

Why this is a concern
The method does not directly infer ecDNA status per cell. It infers amplification mode at the gene level from across cell distributions, then uses a secondary heuristic to label cells. This is not just a wording issue, since downstream conclusions about subclones and states depend on how cell labels are defined. Readers may interpret “single cell ecDNA calling” as per cell inference unless the two stage nature is explicit.

Revisions suggested

Replace language (including in the title) implying per cell ecDNA calling with more precise phrasing such as “infers ecDNA likelihood for amplified genes using distribution features across cells.”

Add a schematic or short box describing the two stages: gene level classification, then cell labeling heuristic.

Provide sensitivity analysis for the cell labeling heuristic, including varying the copy number threshold and alternative definitions such as requiring multiple ecDNA predicted genes, or weighting by predicted probability rather than hard thresholds.

Clarify that “single cell resolution” refers to using single cell data to estimate distributions, rather than per cell amplification mode inference.

3. Simulation based training requires stronger evidence of robustness to biological variation

What the authors show
Training uses simulated distributions where ecDNA undergoes binomial segregation and chromosomal amplifications are stable, with added technical noise and mixtures of ecDNA amplified, HSR amplified, and non amplified populations.

Why this is a concern
Because scAmp is a distribution based classifier using features like variance, IQR, and quantiles, performance will depend on distribution shape, mixture fraction, copy number magnitude, and tail behavior. Real systems span high burden, low burden, and mixed states, and some ecDNA associated loci show relatively modest variance. Without explicit evidence that the simulated distributions reflect the diversity of empirical distributions, it is difficult to know how well decision boundaries will generalize beyond settings that align with the simulation assumptions.

Revisions suggested

Demonstrate that simulated ecDNA and chromosomal distributions resemble empirically observed distributions across a

panel of ecDNA positive cell lines (6+) spanning high burden, low burden, and mixed (ecDNA/HSR) states. The current cell lines are mainly high burden, which is not representative of all tumors/cell lines (I would think these would be the easiest to predict as well). This could be done by comparing distributions using quantile based metrics or Wasserstein distance with uncertainty, alongside visual overlays.

Include a robustness analysis varying key simulation assumptions such as segregation behavior, technical noise regimes, chromosomal copy number instability, and ecDNA mixture fraction, reporting AUROC and AUPRC with confidence intervals and model calibration.

Clearly define expected operating regimes and failure modes, such as sparse ecDNA positive subpopulations or low copy ecDNA, so readers understand where the method should be interpreted cautiously.

4. Validation panel is currently limited in scope for generalizability

What the authors show
Validation focuses on a small number of canonical systems including COLO320 DM versus COLO320 HSR, xenografts derived from these lines, and interphase FISH in FFPE tissue.

Why this is a concern
These experiments demonstrate feasibility and internal consistency, but they do not strongly test generalizability in global settings. COLO320 DM versus HSR is an expected positive control. Xenografts preserve the same underlying genomic architecture. Interphase FISH in FFPE can be suggestive but is not definitive for distinguishing ecDNA from chromosomal or integrated amplification states, especially in mixed or low burden contexts.

Revisions suggested
If the manuscript aims to claim broad discrimination of ecDNA versus chromosomal amplification across tumors, I would encourage a pre-specified validation panel anchored to structure resolving truth. For example:

A blinded set of cell lines (beyond the known and well-studied cases shown here) spanning ecDNA dominant, HSR dominant, and mixed or ambiguous amplification states, including low copy or sparse ecDNA contexts. Structure resolving confirmation by metaphase FISH or an equivalent assay at relevant loci. Application of scAmp to available single cell datasets (or generation of new) for these lines, followed by performance reporting stratified by copy number magnitude and ecDNA fraction.

For FFPE and interphase FISH, frame them as feasibility and explicitly state limitations of predicting ecDNA vs HSR and potential segmentation effects.

5. scATAC derived copy number estimates need explicit limitation and cross modality checks

What the authors show
A major application is to scATAC seq tumor data, using background signal to infer copy number and variance across cells as a discriminative feature.

Why this is a concern
scATAC seq measures chromatin accessibility, not DNA abundance. Accessibility is influenced by chromatin state and transcription. This is particularly relevant for ecDNA since ecDNA is often highly accessible. This raises the possibility that the method captures a composite of copy number and accessibility effects, which could inflate the variance signal in an assay dependent manner and affect classification and downstream biological interpretations.

Revisions suggested

Add an explicit limitation statement that scATAC derived copy number is an assay dependent proxy and may deviate from DNA based estimates.

Where paired modalities exist, compare inferred distributions and scAmp outputs between scATAC and DNA based measurements such as scWGS, ResolveOME, or other suitable assays.

Include at least one control analysis showing that accessibility alone does not trivially drive ecDNA classification, for example by testing highly accessible non amplified regions or applying normalization approaches and assessing stability.

6. Thresholds and terminology would benefit from sensitivity analyses and standardization

Several thresholds are used for feature extraction, amplification calling, ecDNA likelihood, and cell labeling. These should be justified and sensitivity tested. In addition, clarify what is included under “chromosomal amplification,” for example HSRs, BFB derived amplicons, integrated ecDNA like structures, and other focal amplification architectures.

Minor revisions

Replace “improved accuracy over WGS” type statements with more precise language such as concordance with WGS

labels, or improved specificity in selected independently validated cases.

Avoid interpreting disagreement with WGS as evidence that WGS is wrong unless orthogonal validation is presented for those cases.

Ensure figure captions and Results text clearly separate model inputs, model outputs, and post hoc cell labeling heuristics.

Add explicit caveats where scATAC based copy number estimates are discussed, avoiding phrasing that implies direct DNA copy number measurement without qualification.

(Remarks on code availability)

Version 1:

Reviewer comments:

Reviewer #1

(Remarks to the Author)
I thank the authors for their answers and clarifications. I am happy with the revisions and have no further questions. The manuscript introduces a very useful method to identify ecDNA in single cell data.

(Remarks on code availability)

Reviewer #3

(Remarks to the Author)
Review for

scAmp analyzes focal gene amplifications at single cell resolution

The authors have substantially improved the manuscript and addressed the major methodological concerns. The addition of new validation cell lines strengthens the benchmarking framework.

Before publication, I have one remaining concern regarding the provenance and citation of the cell line models used for validation. Because amplification structure (ecDNA versus chromosomal) is central to the claims of the method, each model system should be explicitly anchored to the primary literature that established its structural status (and use as an ecDNA/HSR model). At present, several of the added cell lines are not clearly linked to their originating studies, which limits transparency, reproducibility and recognition of original work.

To facilitate this, I am asking that the authors explicitly cite the following primary references in a way that corresponds to each model system used in benchmarking in the main text.

COLO320DM / COLO320HSR: https://pubmed.ncbi.nlm.nih.gov/498117/ and Turner et al., Nature 2017.

GBM39 (ecDNA and HSR variants): Nathanson et al., Science 2014.

SNU16 and KATOIII: https://aacrjournals.org/cancerres/article/50/9/2773/496583/Characteristics-of-Cell-Lines-Established-from

Sw620: Turner et al Nature 2017 ?

H2170: https://pubmed.ncbi.nlm.nih.gov/39091515/ and https://www.biorxiv.org/content/10.1101/2025.04.26.650733v1

Including these references, along with a brief sentence in the Methods or Results describing how each model was selected and how its amplification status was determined, would significantly improve clarity and transparency. Without this, it is difficult for the reader to assess how ground truth was defined across the validation panel.

(Remarks on code availability)

Open Access This Peer Review File is licensed under a Creative Commons Attribution 4.0 International License, which permits use, sharing, adaptation, distribution and reproduction in any medium or format, as long as you give appropriate credit to the original author(s) and the source, provide a link to the Creative Commons license, and indicate if changes were made.
In cases where reviewers are anonymous, credit should be given to 'Anonymous Referee' and the source.
The images or other third party material in this Peer Review File are included in the article's Creative Commons license, unless indicated otherwise in a credit line to the material. If material is not included in the article's Creative Commons license and your intended use is not permitted by statutory regulation or exceeds the permitted use, you will need to obtain permission directly from the copyright holder.
To view a copy of this license, visit https://creativecommons.org/licenses/by/4.0/

scAmp enables focal gene amplification analysis from single-cell data

Introduction to the Response

We greatly appreciate the time and effort from reviewers and editorial staff for reviewing our manuscript. We are pleased that the reviewers found our study to be "**timely and of importance to the cancer field**", "**of high interest**", and that our method is "**potentially broadly useful**". We are also grateful for the critiques, comments and suggestions. We have addressed each of the points in the revised manuscript, and as a result we believe the paper has now been significantly strengthened. At a high-level, our revision consists of the following:

1. **We restructured the manuscript to improve readability and provided more context on the methods and interpretations of the study**. Several of the reviewer comments centered on expanding our discussion of results, clarifying the interpretation of "ground truth", and contextualizing our method with recent work. We have restructured the manuscript to introduce sections, added a formal Discussion that frames our findings, and provided textual changes that compare our findings and methods to recent work. In this, we underscore the key strengths and areas of future development.

2. **We improved the benchmarking of our method and interpretation of results**. In our revised manuscript, we more explicitly establish when "ground truth" is available, particularly in our characterizations of cell lines using structure-resolving DNA FISH on metaphase spreads. We clarify that our analyses of tumors are by definition limited to assessing concordance and temper our conclusions of applying *scAmp* to FFPE DNA FISH as a proof-of-concept, as suggested by Reviewer 3.

3. **We add new datasets and simulations to bolster our results.** We provide now new single-cell data of ecDNA+ and HSR-dominant cell lines (H2170, Sw620, GBM39ec, and GBM39HSR) that further strengthen our conclusions about *scAmp*'s improved specificity over bulk WGS, and ability to detect integrated amplifications that likely derived from ecDNA. We also perform additional stress-tests, for example assessing *scAmp*'s performance in mixtures of ecDNA+ and ecDNA- cells.

Taken together, these revisions address each of the comments and greatly strengthen our manuscript.

Please find our detailed responses to all reviewer comments in blue below and highlighted within the main PDF file. **Pages of added text and revised figures are bolded and underlined in blue in the response.**

Reviewer #1 (Remarks to the Author):

In the manuscript:"scAmp analyzes focal gene amplifications at single cell resolution", under consideration in Nature Coms, Jones et al. make use of the high single cell variation of extrachromosomal amplified DNA to devise an algorithm for ecDNA identification using single cell ATTAC-seq data. The method is timely and of importance to the cancer field in general. As such the manuscript will be of high interest. I have a few comments to help improve the presentation of the results the authors may want to consider prior to publication.

The readability of the manuscript would benefit from a better structuring of the results section. Also, the discussion seems extremely short and the authors could extend a little.

We are grateful for this reviewer's suggestion and have now restructured the manuscript in the following ways:

- We have added result section headers to orient the reader.
- We have reorganized the figures into a traditional 4 figure format.
- We have added a separate Discussion section that summarizes our insights and incorporates the limitations that we, and the reviewers, have noted.

We believe this restructuring has improved the readability of the manuscript.

The authors implement a neural network architecture to identify tumours with extrachromosomal DNA. This seems to work very well, but feels a bit like shooting with canons on sparrows. Not suggesting changing the analysis here, but out of curiosity, wouldn't grouping by a single number like varianz/mean (high vs. low) suffice to distinguish cases?

We agree with the reviewer's suggestion to better motivate the use of this neural network model. In our initial benchmarking of models, we included a wide variety of classifiers, including a logistic regression model based on the summary statistics extracted from copy-number distributions (including mean, variance, and dispersion). We now include the performance of a simplified linear model based on just mean and variance in our summary of null model performances (revised **Extended Data Fig 5c-d**).

While we see an improvement over the mean-only model, we find that still the variance/mean logistic model cannot accurately disambiguate HSR-like and ecDNA amplifications in our benchmarking cell lines. For example, we find that the variance/mean model incorrectly predicts that *MYC* is ecDNA-amplified in COLO320-HSR and *MYC* in chromosomally-amplified in GBM39-KT. We appreciate this reviewer's suggestion as this analysis further motivates the use of a more complex model that integrates over more statistics than just variance and mean.

c

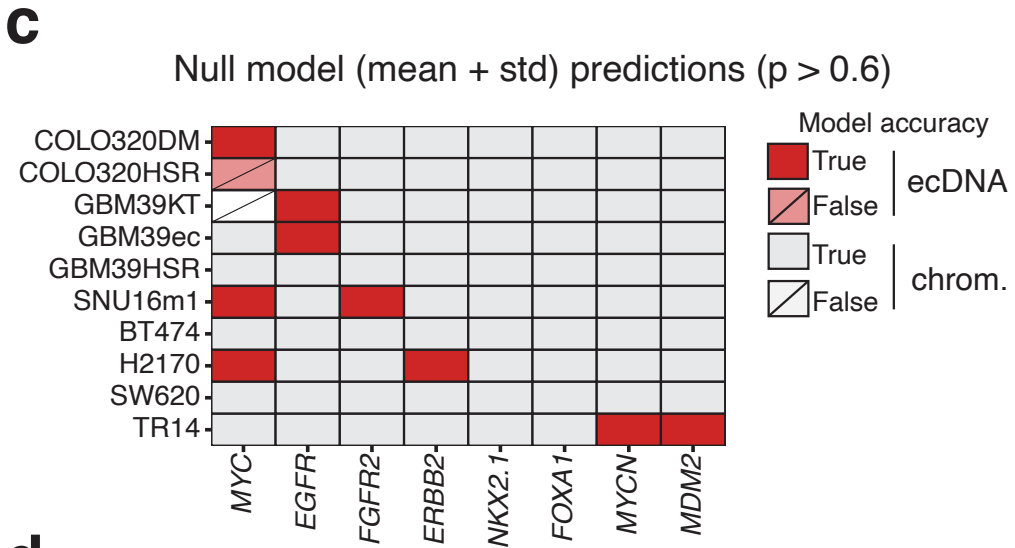
Null model (mean + std) predictions (p > 0.6)
COLO320DM
COLO320HSR
GBM39KT
GBM39ec
GBM39HSR
SNU16m1
BT474
H2170
SW620
TR14
MYC
EGFR
FGFR2
ERBB2
NKX2.1
FOXA1
MYCN
MDM2
Model accuracy
True
False
ecDNA
True
False
chrom.

d

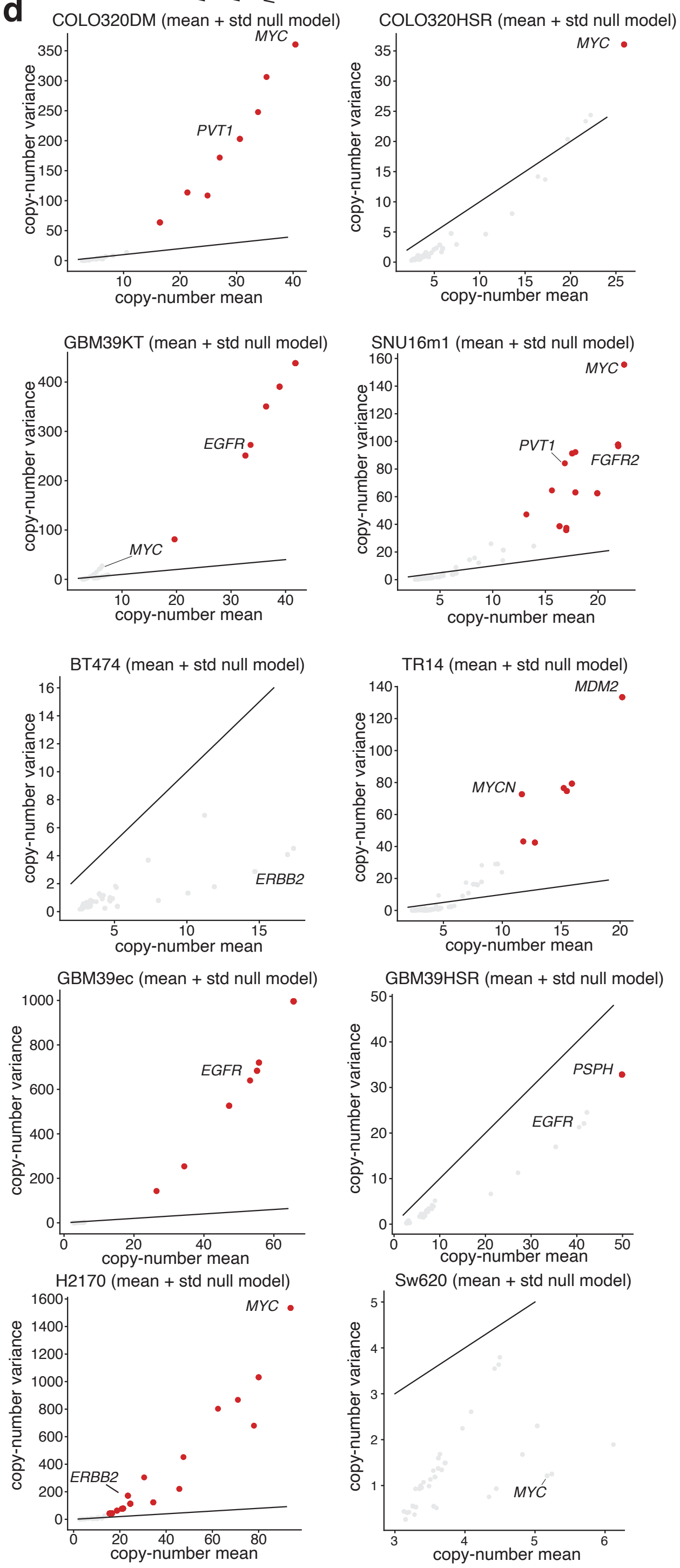
COLO320DM (mean + std null model)
COLO320HSR (mean + std null model)
GBM39KT (mean + std null model)
SNU16m1 (mean + std null model)
BT474 (mean + std null model)
TR14 (mean + std null model)
GBM39ec (mean + std null model)
GBM39HSR (mean + std null model)
H2170 (mean + std null model)
Sw620 (mean + std null model)
copy-number variance
copy-number mean
MYC
PVT1
EGFR
FGFR2
ERBB2
MDM2
MYCN
PSPH

Revised Extended Data Figure 5c-d. *(c-d) Performance of mean+std null linear model (predicting ecDNA status from mean and standard deviation of copy-number distribution) on held-out cell line data. (c) Summary table of mean+std null model predictions of focal genes across cell lines used in this study. Colours and annotations are the same as in (a). (d) Scatterplots summarizing the mean and variance of gene copy-number distributions and mean+std null model predictions in cell lines used in this study. Colours and annotations are the same as in (b).*

The authors say they can estimate the fraction of ecDNA carrying cells in the sample. There are two questions, (i) the authors use a cutoff of at least 4 ecDNA copies per cell. However, if I understood correctly, ecDNA copy number heterogeneity also allows for cells with 1 to 3 copies, thus this cutoff will underestimate the fraction? (ii) Does the tumour microenvironment bias these results? For example, a sample could contain a high fraction of immune infiltrating cells that bias estimates?

We thank the reviewer for asking these clarifying questions as these are important for the interpretation of our analyses. To clarify, the estimation of ecDNA+ cancer cell fraction is only on the non-immune or -stromal cell fraction, thus mitigating concerns about immune infiltrating cells biasing estimates. We use the cell-type labels presented in the original paper (Sundaram et al, *Science* 2024).

Next, in labeling cells as ecDNA+, we must consider that every cell's estimated copy-number includes both normal chromosomal DNA as well as ecDNA copies. Since a normal region would be expected to have a copy-number of 2, we use a copy-number cutoff of 4 (representing a conservative gain of 2 additional copies) to identify cells carrying ecDNA. In effect, this labeling strategy assigns ecDNA status to cells if they have at least 2 copies of ecDNA which is a conservative estimate. In our analyses, we observed that going below this threshold (i.e., 1 additional copy) might result in an overestimation since the copy-number distribution is continuous and most normal regions of the genome have values between 2-3. As discussed above, however, we agree that this parameter is an important one to characterize and now present an analysis of the frequency of ecDNA+ cancer cells with varying copy-number thresholds (revised **Extended Data Figure 9d**).

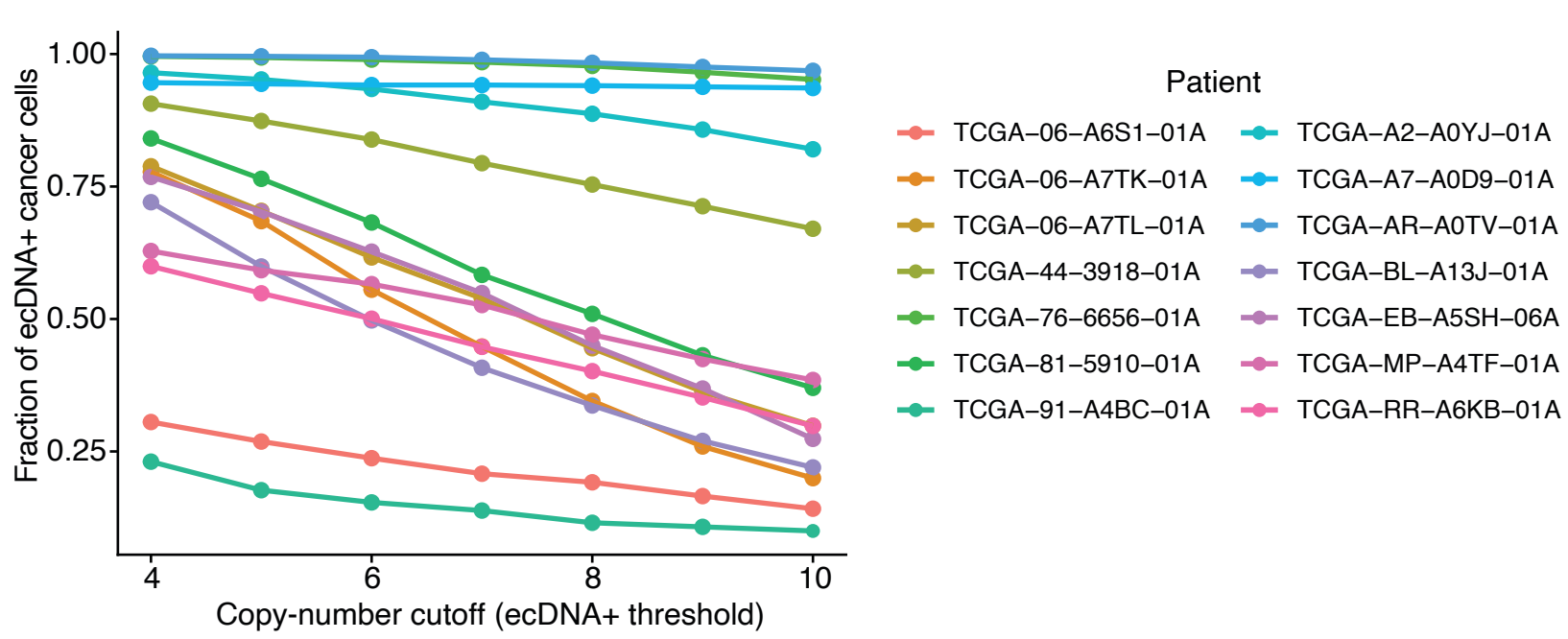

Revised Extended Data Figure 9d*. (d) Fraction of ecDNA+ cancer cells detected in each ecDNA+ tumour with varying copy-number thresholds. Each line indicates a different ecDNA+ tumour.*

The authors say their method can help timing the occurrence of ecDNAs, but somehow the discussion lacks details. I assume you mean to say that a high fraction of ecDNA+ cells suggest early arrival? Again this might be biased by tumour microenvironment and/or effects of spatial sampling. I think in this context it will also be useful to give exposure to the works of Okonechnikov et al.Nature 2025 and Noorani et al Cancer Discovery 2025.

This is an excellent point. First we clarify that, as above in evaluating the ecDNA+ cancer cell fraction, the clustering analysis is done with respect to only cancer cells (i.e., after removing immune and stromal cells). We base our interpretation of the data off of a minimum evolution perspective whereby ecDNA that are shared by more cells are most likely to have arisen first. However, as the reviewer notes, we cannot quantify the absolute timing of ecDNA emergence, nor whether they occurred early or late in tumor progression. Indeed, even a late-occurring ecDNA under very strong selection may sweep the population and appear to have occurred early.

We agree that this is an important point to clarify and in our presentation of our tool, we alter the language in several places to reflect this prior literature:

On **p. 6-7**:

> *Recent literature has highlighted the functional importance of ecDNA in driving distinct stages of tumor progression, and the ability to detect ecDNA on a cell-by-cell basis provides the opportunity to infer the subclonal evolution of ecDNA within tumors.*

And in our Discussion on **p. 8-9**:

> *Second, in our retrospective analysis of patient tumors profiled with scATAC-seq, we found that ecDNA+ cancer cell fractions were highly variable (ranging between 10% to 100%) and that some tumors harbored evidence of ongoing subclonal evolution of new ecDNA (Figure 3c-d). This finding is reminiscent of the ongoing evolution of ecDNA amplifications observed medulloblastoma and glioblastoma, and along with other recent large-scale analyses of scWGS data, point to the utility of single-cell assays to study the relative timing and influence of amplification evolution in cancer.*

The authors specifically discuss that they found ERBB2 in a breast cancer cell line to be chromosomally amplified and not on ecDNA. This is interesting and potentially important, especially as it has been suggested recently that a high proportion of Her2+ Breast Cancers carry ecDNAs (Houlahan et al. Nature 2025). I appreciate, the example in the manuscript here is n=1 which does not allow to generalize. Is there a possibility to acquire other sample or data. Do the observations here have possible implications for the interpretation of the results in Houlahan et al.?

Once again this is an excellent point, and we believe accents the strength of single-cell assays for ecDNA detection. Our findings in the case of *ERRB2* chromosomal integration in the BT474 cell line highlights that bulk sequencing cannot disambiguate between amplifications that currently exist as ecDNA versus those that historically were ecDNA and have since integrated into the chromosomes. In our revised manuscript, we highlight that this can also be observed in the case of COLO320-HSR, the isogenic, chromosomally-amplified variant of the ecDNA+ COLO320-DM line: bulk WGS incorrectly calls the *MYC* amplicon as ecDNA (https://ampliconrepository.org/project/650086713d950b28f5361766/sample/COLO320HSR).

We further bolster these analyses with new single-cell sequencing data of the glioblastoma GBM39ec/HSR isogenic pair, the non-small-cell lung cancer H2170 cell line, and the colorectal cancer cell line Sw620 (revised **Extended Data Figure 1** and **Extended Data Figure 7**). We show that *scAmp* accurately predicts amplicon status in all of these cell lines, including chromosomal amplifications of *EGFR* in GBM39HSR, *NKX2.1/FOXA1* in H2170, and *MYC* in Sw620. On the other hand, bulk WGS approaches incorrectly assign ecDNA status to *EGFR* in GBM39HSR and *MYC* and Sw620 (revised **Extended Data Figure 7d-f**). Reassuringly for WGS-based approaches, we find that both *scAmp* and bulk WGS are able to identify the amplicon containing *NKX2.1* and *FOXA1* in H2170 as chromosomally-amplified (AmpliconRepostory summary for H2170 is available here: https://ampliconrepository.org/project/68d06aa57116a5e41cb99d38/sample/NCIH2170) These analyses demonstrate that bulk WGS analyses are not always wrong at identifying chromosomal amplifications (e.g., the *NKX2.1* amplification in H2170), single-cell analysis and *scAmp* show improved specificity in two new cases.

Thus, while we cannot generalize these results to the ecDNA classification accuracy of HER2+ BRCA tumors in other studies without larger characterization, we note that another recent preprint has also found that ecDNA status in BRCA tumors are commonly misclassified by bulk WGS analysis (Lee et al, *BioRxiv* 2026). As we explain in the manuscript, one possible explanation for this discrepancy is that ancestral ecDNAs that have integrated back into chromosomes retain the breakpoints and copy number profiles of ecDNA amplicons but follow the copy number distributions of chromosomal amplifications. Thus bulk WGS may provide a “historic” view of the ecDNA status of a tumor while single-cell measurements provide insight into the current state of the tumor. Together, these analyses should underscore the importance of single-cell measurements in accurately classifying ecDNA status of tumors. While single-cell genomics assays may not be feasible for most studies, we highlight that the proof-of-concept classification based on single-cell copy numbers derived from DNA FISH may pave the way forward for widely-accessible validation of clinical samples. We now include these important points in our revised and extended Discussion:

> *“Our analyses with scAmp revealed several new insights into ecDNA dynamics. First, in our analysis of cell lines, we found that there exist a class of*

chromosomal amplifications that are misclassified by bulk assays but correctly predicted with scAmp (e.g., ERBB2 in BT474 and MYC in Sw620; Extended Data Figure 7). These data suggest that some chromosomal amplifications may have historically been ecDNA (perhaps related to the plasticity of amplification modes in the course of drug treatment) and underscore that single-cell data will be critical for resolving these cases."

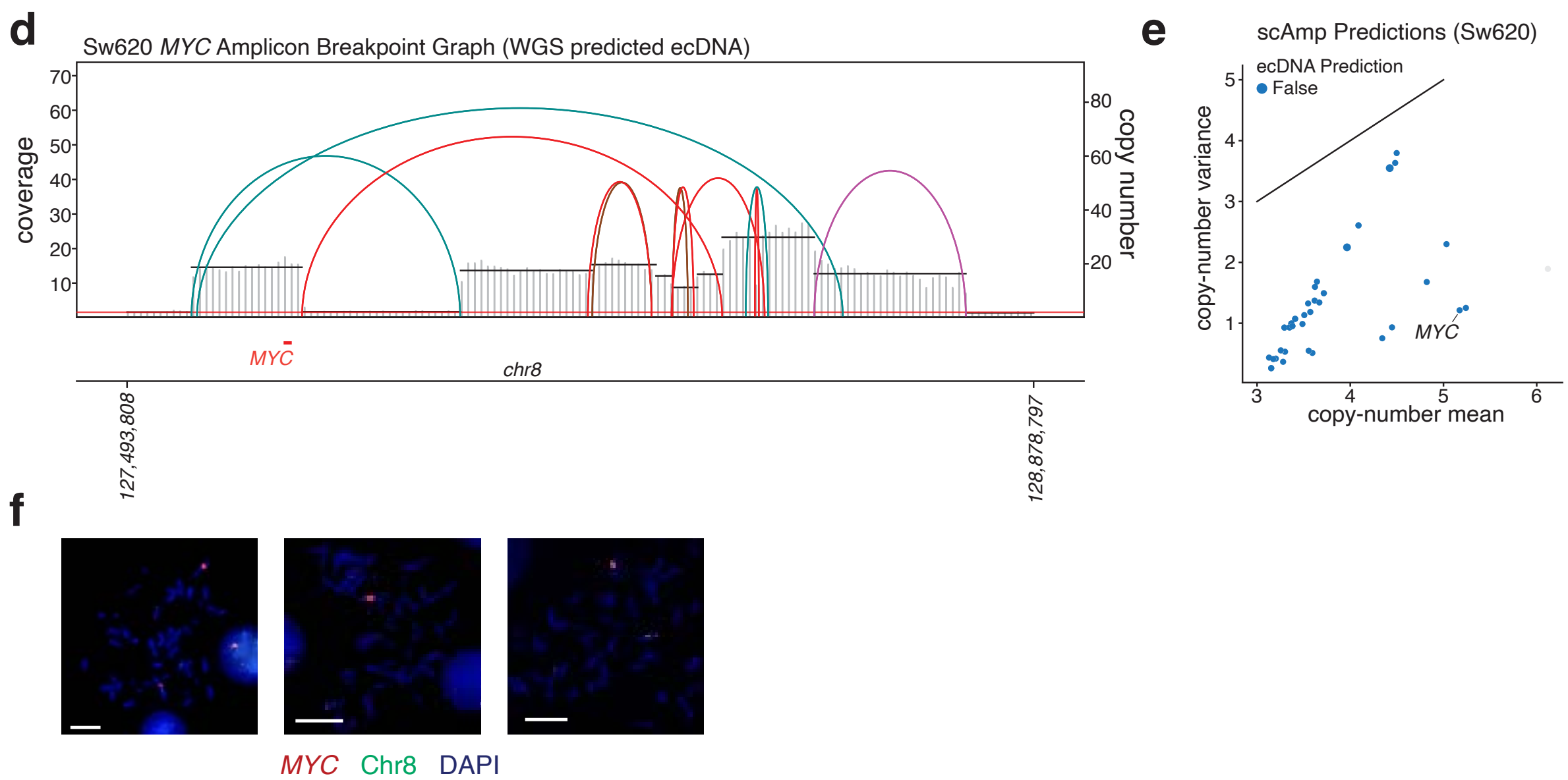

Revised Extended Data Figure 7d-f. *(d) A breakpoint graph of the MYC amplicon in Sw620 predicted by AmpliconArchitect10 analysis of WGS data as ecDNA+ (obtained from AmpliconRepository). The coverage over the amplified intervals is shown as a grey distribution, and each arc represents a breakpoint inferred from AmpliconArchitect. Only the MYC gene locus is shown underneath for clarity. (e) Scatterplots summarizing the mean and variance of gene copy-number distributions for each gene in the Sw620 cell line, along with predictions from scAmp. MYC is predicted to be chromosomally amplified. (f) Metaphase spread images of cells with DNA FISH for MYC and centromere 8 probe. MYC co-localizes with DAPI+ chromosomal signal and is inferred to be chromosomally integrated in Sw620. Scale bars indicate 10μm.*

Reviewer #2 (Remarks to the Author):

Matthew Jones and colleagues in their manuscript present scAmp (“single-cell amplicon” analysis), a probabilistic algorithm for detecting and analyzing extrachromosomal DNA (ecDNA) from single-cell datasets.

While genomic alterations are well known and highly studied hallmark of cancer, ecDNA has more recently emerged as a critical mechanism promoting oncogene amplification, tumor progression, and resistance to therapy.

Despite its importance, the mechanisms by which ecDNA contributes to intratumor heterogeneity and clonal selection are largely unknown. New methodological approaches to analyse ecDNA could therefore provide critical insight and advance our understanding of these fundamental processes.

Leveraging machine learning approaches, using copy-number distributions data and exploiting one of the major features of ecDNA biology – the non-Mendelian inheritance of individual ecDNA molecules during cell division – the authors formulated a model which was able to correctly predict the likelihood of genes being amplified in ecDNA from those deriving from chromosomal amplification, in single-cell assays, such as of scWGS or scATAC-seq. They successfully validate their model in tumor samples from The Cancer Genome Atlas (TCGA) with paired WGS data.
Applying scAmp to single-cell ATAC-seq experiments they were also able to perform phenotypic analyses of cell states within ecDNA+ tumors, which provided the opportunity to infer the subclonal heterogeneity of amplified oncogenes in ecDNA within tumors.

In general, the methodological approach is clearly described and logically structured. The authors introduce the method in a stepwise manner that allows the reader to understand both its conceptual motivation and its practical implementation. Key components of the workflow are well illustrated, and they clarify the underlying logic without unnecessary complexity. The writing is precise and focused, which makes the technically sophisticated method accessible to a broad readership.
The two main figures and the Extended Data Figures are presented with a high level of completeness and precision. They are carefully designed, clearly annotated, and effectively support the main text and provide valuable methodological and technical detail that facilitates interpretation of the results and enhances transparency. Overall, the quality and organization of the visual material significantly strengthen the manuscript. The validation in patient tumors suggests that the method presented it is solid and potentially broadly useful.

That said, given that the primary contribution is methodological, the manuscript would benefit from a more explicit statement of the methodological novelty. In particular, the authors should clearly articulate which specific limitations of existing approaches are addressed by the proposed method, and in what sense these capabilities are not currently available.

We are grateful for the reviewer's positive assessment of our study, and have now updated our manuscript to better reflect the methodological advance and potential limitations.

Briefly, *scAmp* offers two significant advances over traditional methods. First, while previous approaches that have used bulk WGS to study focal amplifications, *scAmp* offers a principled approach for classifying focal amplifications from a variety of single-cell data (including scATAC, scWGS, and as we show in a proof-of-concept analysis, DNA FISH) and studying the subclonal composition of ecDNA+ cancers. The single-cell methodology also aids in the identification of a new class of chromosomal amplifications that appear to have been historically extrachromosomal; we now include a more thorough presentation of these results. Second, as compared to other recent methods designed for specific assays (e.g., the approach linked below designed for scRNA-seq), since *scAmp* is based on copy-number distributions it is platform agnostic, for example copy-number signal obtained by scATAC-seq and DNA FISH in the case of our study. On the other hand, we show in our study that by using scATAC as the platform for estimating copy-numbers, we are also able to link the molecular status of a cell to its focal amplifications. This is in contrast to other recent preprints (e.g., Lee et al, BioRxiv 2026) that use scWGS to study ecDNA: while they can elegantly dissect the genomic evolution of ecDNA, they cannot infer the molecular state of cells. We believe this will be an important aspect of *scAmp* analyses in the future.

Still there are important considerations we have identified for running *scAmp.* First, we have revised our manuscript to reflect that *scAmp's* model predicts the likelihood of a gene amplified on ecDNA, and then uses a heuristic-based copy-number thresholding scheme for labeling cells (see revised **Figure 1a**). Second, we have new insights into the robustness of *scAmp* at varying levels of ecDNA+ purity. In a new experiment for our revised experiment, we tested *scAmp*'s performance on *in silico* mixtures of cell lines that contain either ecDNA-amplified or chromosomal-amplified genes: while we find that *scAmp* performs very well overall, the detection limit appears to be around 20% ecDNA+ fraction (revised **Extended Data Figure 6b**).

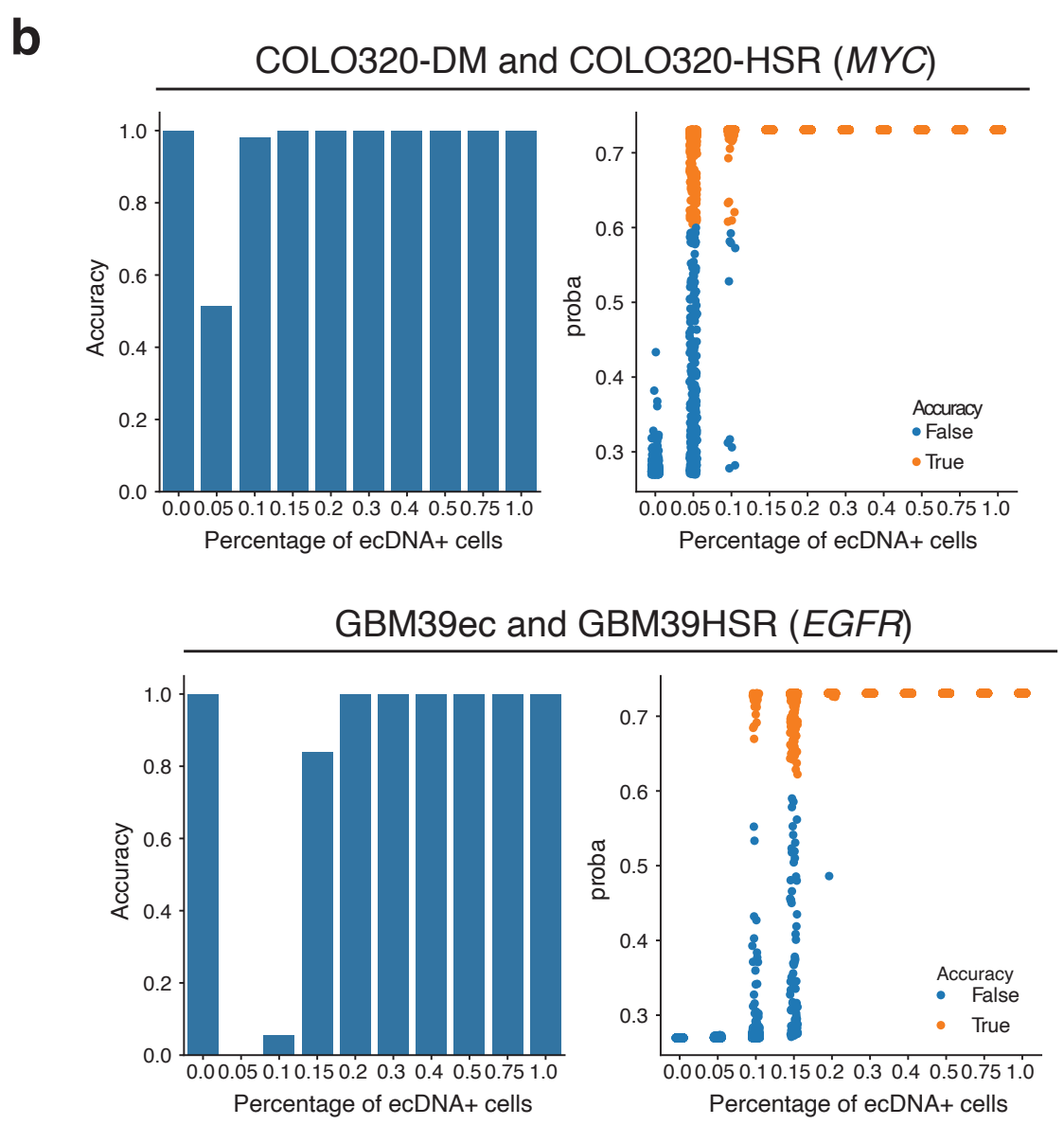

***Revised Extended Data Figure 6b**. Performance of scAmp model on synthetic mixtures of ecDNA+ and HSR+ cell lines (MYC in COLO320-DM/HSR and EGFR GBM39ec/HSR, top and bottom respectively). Model accuracy (left) and probability of amplification (right) are shown for varying amounts of ecDNA+ fraction.*

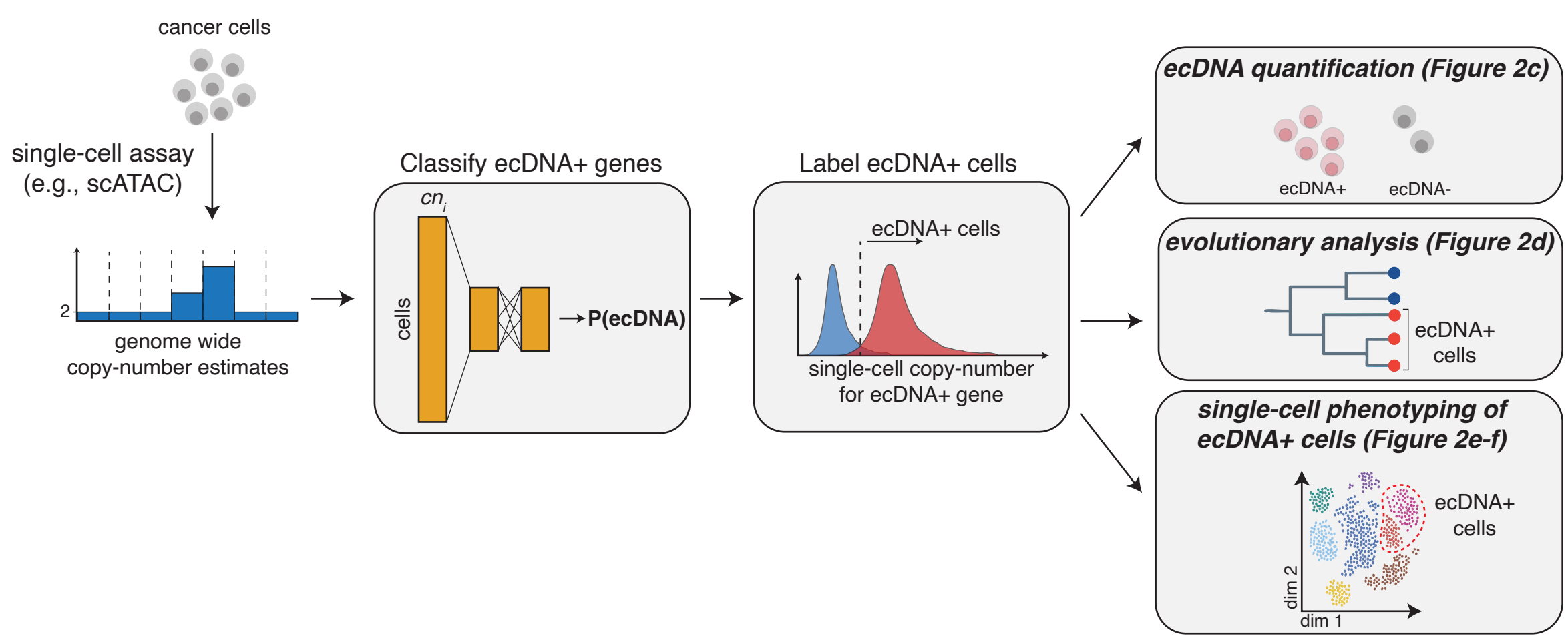

***Revised Figure 1a**. An overview of the scAmp workflow. Copy numbers are inferred from individual cells (e.g., from single-cell ATAC-seq data). For each gene, scAmp then infers the likelihood of that gene being amplified on ecDNA based on the properties of its copy-number distribution across cells. Then, cells are labelled as ecDNA+ or ecDNA- based on a heuristic thresholding scheme. This enables downstream analyses such as quantifying the prevalence of ecDNA in a tumour, assessing an ecDNA's evolutionary history, and investigating the phenotypic effects of ecDNA on cancer cell state.*

Together, we have included these points throughout our revised manuscript:

Regarding the introduction of *scAmp* and contextualizing with other recent single-cell tools (**p. 3):**

> *"scAmp operates on single-cell copy-number data (which can be obtained from assays such as single-cell WGS or single-cell assay for transposase-accessible chromatin by sequencing [scATAC-seq]) and predicts a likelihood for each gene that it is amplified on ecDNA. This principle is similar to algorithms for predicting ecDNA-amplified regions from allelic imbalance in single-cell gene expression data, though the focus on copy-number distributions extends across many platforms. From these predicted ecDNA amplifications, cells can be labeled as ecDNA+ based on their copy-numbers (for example, a threshold based on copy-number heuristics."*

In comparison to bulk WGS-based approaches, and the higher specificity of *scAmp* (**p. 5-6**):

> *"Indeed, the difficulty of WGS-based assays predicting amplification status of integrated ecDNA is also observed in the case of COLO320HSR and GBM39HSR - isogenic variants of ecDNA+ cell lines in which the ecDNA-amplified gene has integrated into the chromosome (Extended Data Figure 1; Figure 2e). These results demonstrate that there exists a class of historical ecDNA amplifications that single-cell characterization with scAmp can resolve with higher specificity than bulk sequencing approaches alone."*

We also have added an in-depth Discussion to our manuscript, that includes a presentation of our main contributions and limitations. The relevant sections are included here (**p. 8-9):**

> *"Our analyses with scAmp revealed several new insights into ecDNA dynamics. First, in our analysis of cell lines, we found that there exist a class of chromosomal amplifications that are misclassified by bulk assays but correctly predicted with scAmp (e.g., ERBB2 in BT474 and MYC in Sw620; Extended Data Figure 7). These data suggest that some chromosomal amplifications may have historically been ecDNA (perhaps related to the plasticity of amplification modes in the course of drug treatment) and underscore that single-cell data will be critical for resolving these cases. Second, in our retrospective analysis of patient tumors profiled with scATAC-seq, we found that ecDNA+ cancer cell fractions were highly variable (ranging between ~10% to 100%) and that some tumors harbored evidence of ongoing subclonal evolution of new ecDNA (Figure 3c-d). This finding is reminiscent of the ongoing evolution of ecDNA amplifications observed medulloblastoma and glioblastoma and, along with other recent large-scale analyses of scWGS data, point to the utility of single-cell assays to study the relative timing and influence of amplification evolution in cancer. Third, we leveraged the molecular insights gained from scATAC-seq to identify putative gene expression programs associated with ecDNA, including increased glycolysis and hypoxia signaling (Extended Data Figure 9). Finally, we introduce*

> *a proof-of-concept analysis demonstrating that scAmp's principles may also enable patient stratification using measurements gained from clinically-relevant modalities like DNA FISH on FFPE samples.*
>
> *Beyond the potential of scAmp there are important limitations and future directions to acknowledge. First, scAmp's workflow relies on predicting ecDNA status of individual genes and then heuristically labeling cells based on copy-number. This may conflate cells that have chromosomal amplifications with those that contain ecDNA, and thus new algorithms that deconvolve these two populations will be useful. Second, while scATAC-seq has been used widely for copy-number estimation, it is sparse and does not provide as robust copy-number quantification as scWGS approaches that directly measure genetic content. Third, the benchmarking of scAmp in tumors from the TCGA dataset is limited by the lack of gold-standard validation of ecDNA status (i.e. DNA FISH on metaphase spreads). Lastly, while our analysis of DNA FISH in FFPE tissue is promising, it remains a proof-of-concept and generalizability to clinical samples will require more thorough benchmarking."*

Together, we believe these additions that the reviewer suggested have markedly improved the readability and interpretation of our manuscript.

I note that a very recent study published during the review process (*) appears to address a closely related objective. While this does not diminish the value of the present work, it would be probably important to acknowledge this study and to clarify the relationship between the two approaches, highlighting similarities and differences in scope, implementation, and applicability.
(*) https://doi.org/10.1186/s13059-026-03933-2

We thank the reviewer for raising this paper to our attention and have now edited the manuscript to better contextualize our advances compared to other methods (see response above). We have included a referent to this recent paper in our revised manuscript (**p.3**):

> *"scAmp operates on single-cell copy-number data (which can be obtained from assays such as single-cell WGS or single-cell assay for transposase-accessible chromatin by sequencing [scATAC-seq]) and predicts a likelihood for each gene that it is amplified on ecDNA. This principle is similar to algorithms for predicting ecDNA-amplified regions from allelic imbalance in single-cell gene expression data, though the focus on copy-number distributions extends across many platforms. From these predicted ecDNA amplifications, cells can be labeled as ecDNA+ based on their copy-numbers (for example, a threshold based on copy-number heuristics."*

Overall, I am positive about the manuscript, and I believe it will be suitable for publication after the last suggested very minor revisions.

Reviewer #3 (Remarks to the Author):

Review for

scAmp analyzes focal gene amplifications at single cell resolution

Summary

This manuscript introduces scAmp, a method designed to infer whether focal gene amplifications are carried on extrachromosomal DNA (ecDNA) versus chromosomal amplification states such as homogeneous staining regions (HSRs) using single cell datasets. The core idea is that ecDNA's non Mendelian inheritance can increase cell to cell copy number variability, and that summary features of gene level copy number distributions across cells can be used to classify amplification mode. The authors train a supervised classifier largely on simulated copy number distributions, apply it to selected cell lines and TCGA scATAC seq tumor datasets, and present downstream analyses of heterogeneity and chromatin accessibility.

The biological question is important and the conceptual approach is interesting. At the same time, several aspects of the current training, benchmarking, validation, and framing limit how confidently the central claims can be interpreted. In my view, the manuscript would benefit from revisions that sharpen what is and is not being inferred at the gene versus cell level, strengthen independent validation, more explicitly address assay dependent limitations of scATAC derived copy number estimates, and include standard sensitivity and uncertainty analyses expected for ML based methods. In particular, robust differentiation of ecDNA versus chromosomal amplification across tumors and across assays is not established with the current training and validation strategy. I recommend major revision. If the validation additions below are not feasible, the manuscript should be reframed as a proof of concept in a limited set of canonical models and the language should be scaled accordingly. I would consider the structure anchored validation panel and the cross modality check to be required for supporting the headline claim of robust ecDNA versus chromosomal amplification discrimination in tumors.

Major concerns
1. Benchmarking and ground truth for tumors are not independent

What the authors show
For tumors, scAmp is evaluated against WGS based calls such as AmpliconSuite or AmpliconClassifier, and the manuscript also discusses cases in which bulk WGS may misclassify ecDNA versus integrated or ancestral ecDNA derived amplification states.

Why this is a concern
If the comparison labels are derived from the same class of bulk WGS approaches whose limitations are discussed, then performance metrics in tumors are best interpreted as concordance with WGS derived labels, rather than definitive accuracy.

The BT474 example is useful in highlighting that WGS based labels can be ambiguous, but it also underscores the need for independent truth sets when reporting accuracy like metrics.

Revisions suggested

Adjust wording in the Abstract and Results so tumor benchmarking is described as concordance with WGS based calls rather than accuracy, unless orthogonal truth is provided.

This is an excellent point, and we thank the reviewer for suggesting this revision of wording. We agree that bulk WGS is not a ground truth, and indeed this is a major feature of the manuscript. We have refined this point in three ways:

- First, as the reviewer suggested, we have amended the text to refer to “concordance” with bulk WGS when other ground truth is not available, such as with the TCGA analysis. For example on **p. 4**:

  “To refine our model selection, we assessed the concordance of predictions from single-cell and bulk WGS assays in tumor samples from the Cancer Genome Atlas (Extended Data Figure 2b-e; Extended Data Figure 3).”

  And on **p.6:**

  “As reported above, we found that scAmp’s predictions were largely concordant with bulk WGS data and improved over Random Forest predictions (80% agreement vs 70% on a per-gene level, and 79% vs 32% that a tumor contained at least one ecDNA-amplified gene; Extended Data Figure 5; Extended Data Figure 8a)”

  Also, in our Discussion, we note the lack of ground truth in the case of TCGA tumors as follows (**p.9**):

  “Third, the benchmarking of scAmp in tumors from the TCGA dataset is limited by the lack of gold-standard validation of ecDNA status (i.e. metaphase FISH).”

- Second, we have clarified that the current gold standard for assessing ecDNA status is DNA FISH on metaphase spreads. All of the cell lines presented in this manuscript and used for benchmarking have been characterized with this assay and we now include representative images of these experiments to accompany our scAmp-based analyses (revised **Extended Data Figure 1c**).

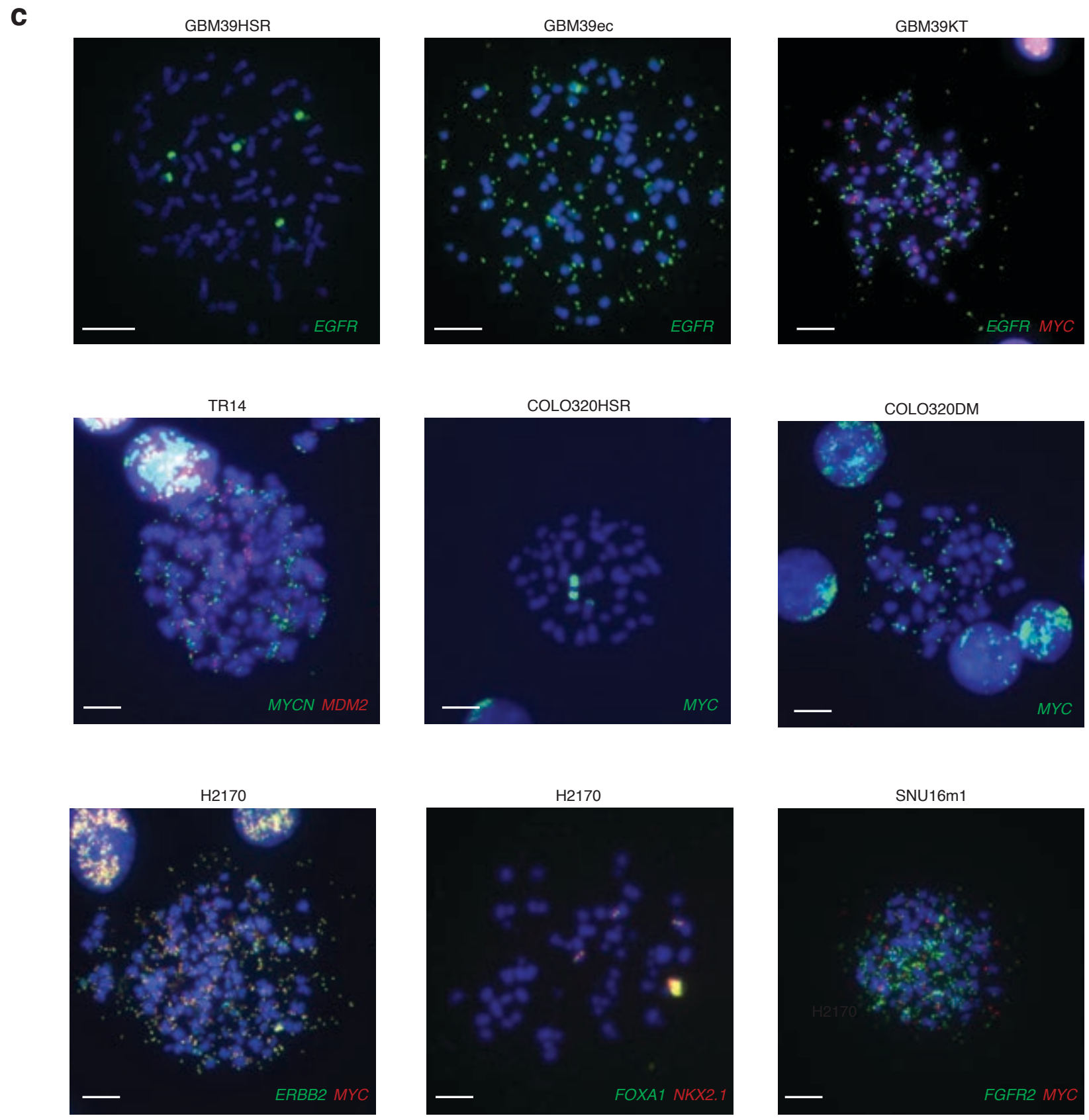

***Revised Extended Data Figure 1c**. (c) Representative images of DNA FISH on metaphase spreads from previous studies demonstrating the amplification mode of key oncogenes used for testing model predictions.*

- Third, we have altered our summary of benchmarking to now show agreement of *scAmp* and WGS with the gold-standard metaphase FISH characterization (revised **Figure 2e**). This figure emphasizes that bulk WGS can erroneously classify amplicons if they were historically amplified as ecDNA (i.e., COLO320-HSR and GBM39HSR) while scAmp predictions are consistent with all metaphase FISH results. We bolster this point with a revised **Extended Data Figure 7**, showcasing another example in which single-cell analysis identifies a chromosomal amplification carrying the sequence features of an ecDNA (*MYC* in Sw620).

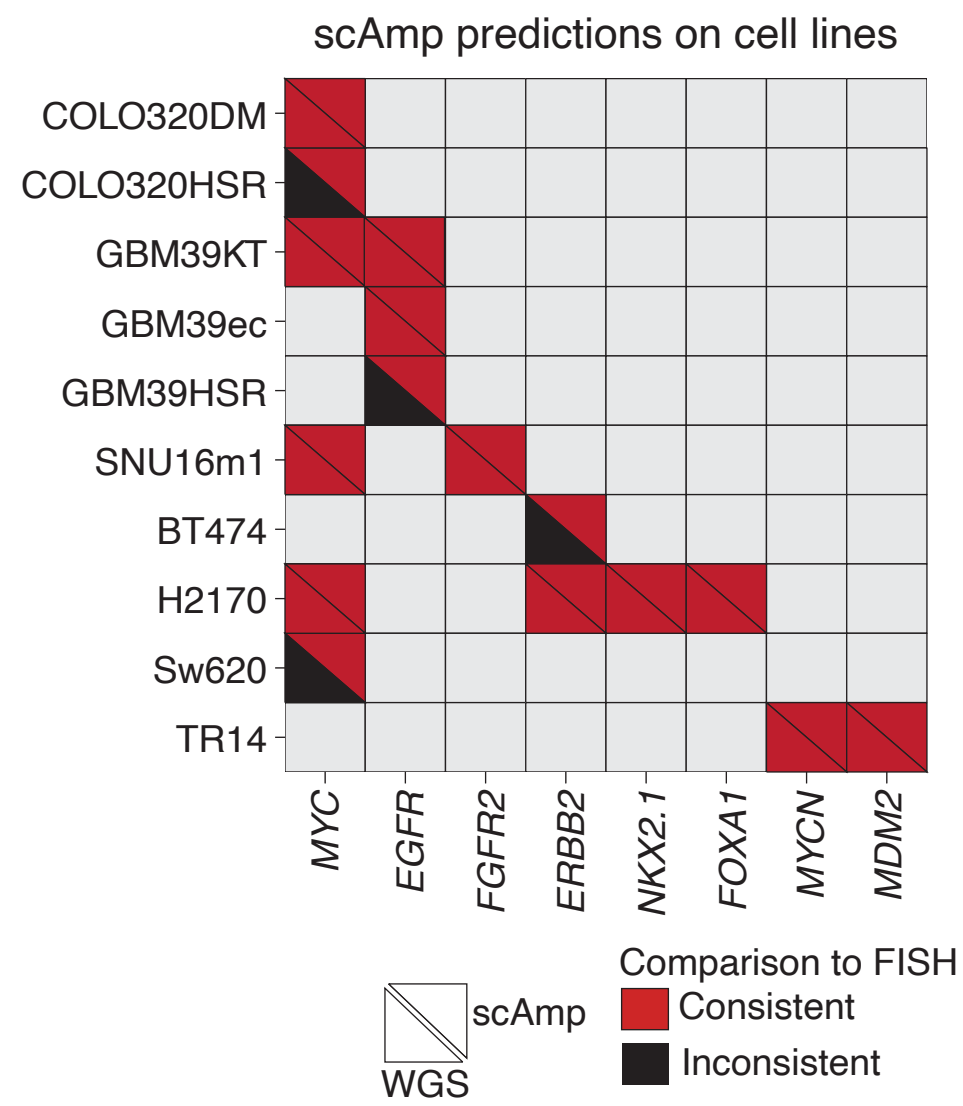

***Revised Figure 2e**. (e) Summary gene-level predictions from scAmp and bulk WGS across cell lines. Agreement between an assay and DNA FISH on metaphase spread ground-truth are marked in red; disagreement is marked in black.*

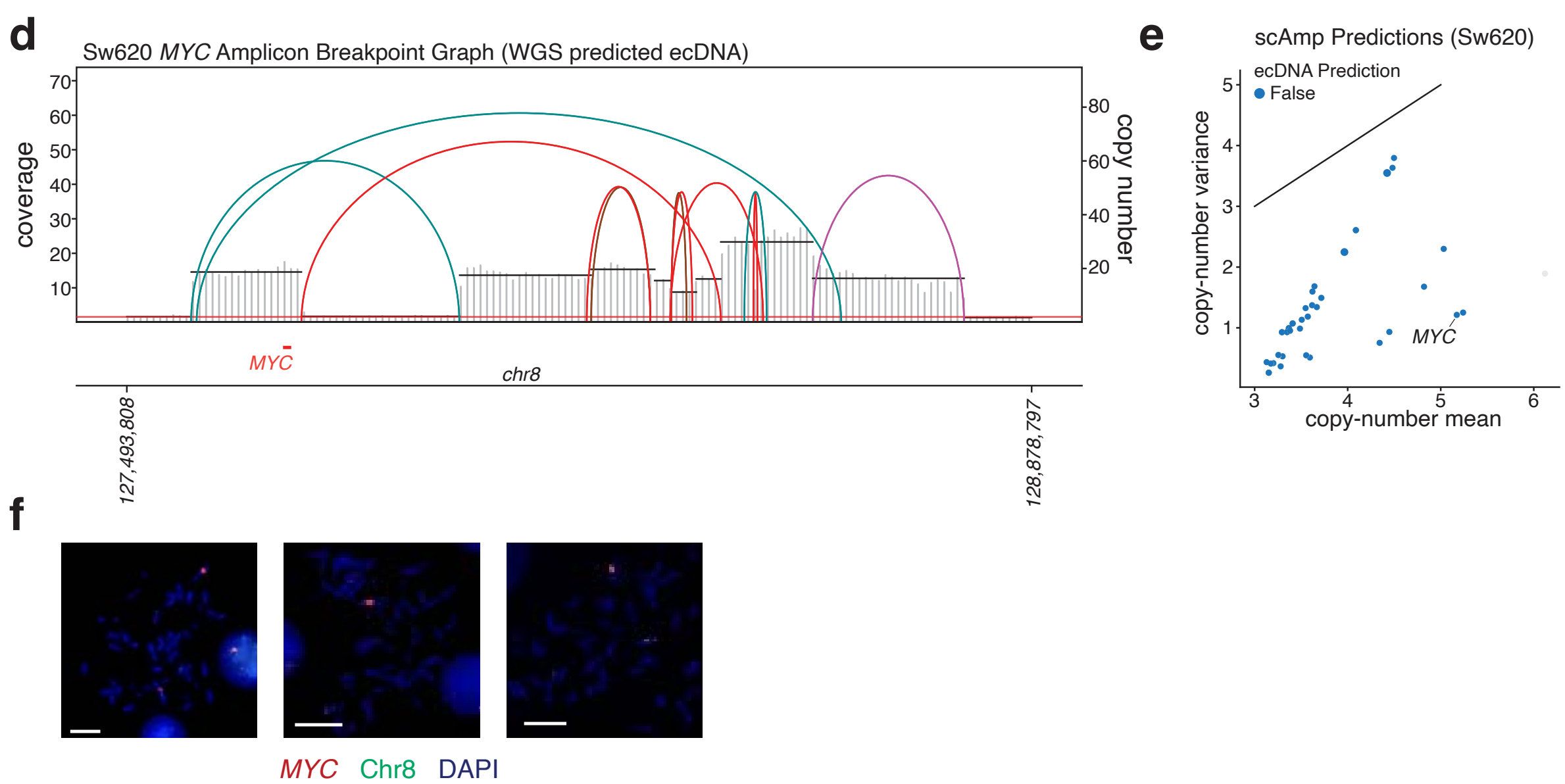

***Revised Extended Data Figure 7d-f.** (d) A breakpoint graph of the MYC amplicon in Sw620 predicted by AmpliconArchitect10 analysis of WGS data as ecDNA+ (obtained from AmpliconRepository). The coverage over the amplified intervals is shown as a grey distribution, and each arc represents a breakpoint inferred from AmpliconArchitect. Only the MYC gene locus is shown underneath for clarity. (e) Scatterplots summarizing the mean and variance of gene copy-number distributions for each gene in the Sw620 cell line, along with predictions from scAmp. MYC is predicted to be chromosomally amplified. (f) Metaphase spread images of cells with DNA FISH for MYC and centromere 8 probe. MYC co-localizes with DAPI+ chromosomal signal and is inferred to be chromosomally integrated in Sw620. Scale bars indicate 10µm.*

We believe these additional figures and changes to text address allow readers to properly interpret our benchmarking efforts.

Add a short subsection stating clearly that tumor labels are WGS derived and that these labels are not definitive for ecDNA versus integrated ecDNA like structures, so AUROCs in tumors should be interpreted accordingly.

We agree that this is a critical clarification, and have amended the text and figures (revised **Extended Data Figure 2**). Our textual changes are listed above (see earlier response) and include a specific callout in the Discussion of our revised manuscript (**p.9**):

> *"Third, the benchmarking of scAmp in tumors from the TCGA dataset is limited by the lack of gold-standard validation of ecDNA status (i.e. DNA FISH on metaphase spreads)."*

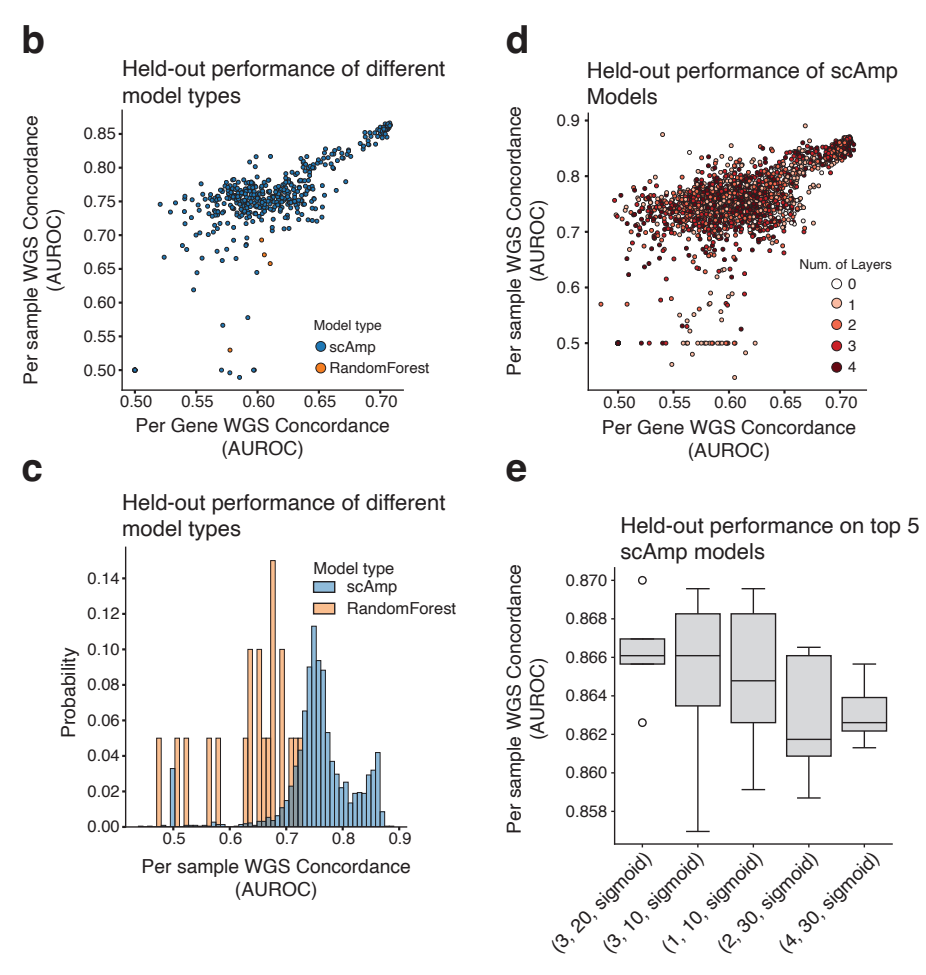

Revised Extended Data Figure 2b-e*. (b) Comparison of performance for all trained scAmp and RandomForest classifier models, as quantified by AUROC of per-sample or per-gene ecDNA concordance with held-out patient tumour scATAC-seq data from TCGA. Per-sample ecDNA concordance quantifies the agreement between a model and bulk WGS in predicting whether a patient has at least one ecDNA amplicon; per-gene ecDNA concordance quantifies the agreement between a model and bulk WGS of predicting whether a gene is amplified on ecDNA or not (Methods). (c) Histogram of AUROC of per-sample ecDNA concordance on held-out patient tumour scATAC-seq data for both RandomForest classifiers and scAmp models. (d) Scatterplot of scAmp model performances colored by number of hidden layers in scAmp model. (e) Boxplot of the top 5 scAmp models, as measured by the AUROC on the per-sample ecDNA concordance task for held-out patient tumour samples. The number of layers, size of hidden layer, and activation function are indicated for each top model. Individual points indicate the performance of a training replicate (i.e., training a model with a new random*

seed). Boxplots report the quartiles of the distributions, and whiskers extend to 1.5x the interquartile distance.

Provide at least one independent validation setting where ecDNA versus chromosomal amplification is established by a structure resolving approach rather than WGS calling alone.

As above, we agree that it is important to note that WGS is not an absolute ground truth whereas DNA FISH on metaphase spreads (i.e., a structure-resolving approach) is the current gold-standard. We now provide several independent validations of *scAmp* calls using this assay, including on new cell lines:

- First, we now include representative metaphase DNA FISH images for all the cell lines characterized in this study (revised **Extended Data Figure 1c**).

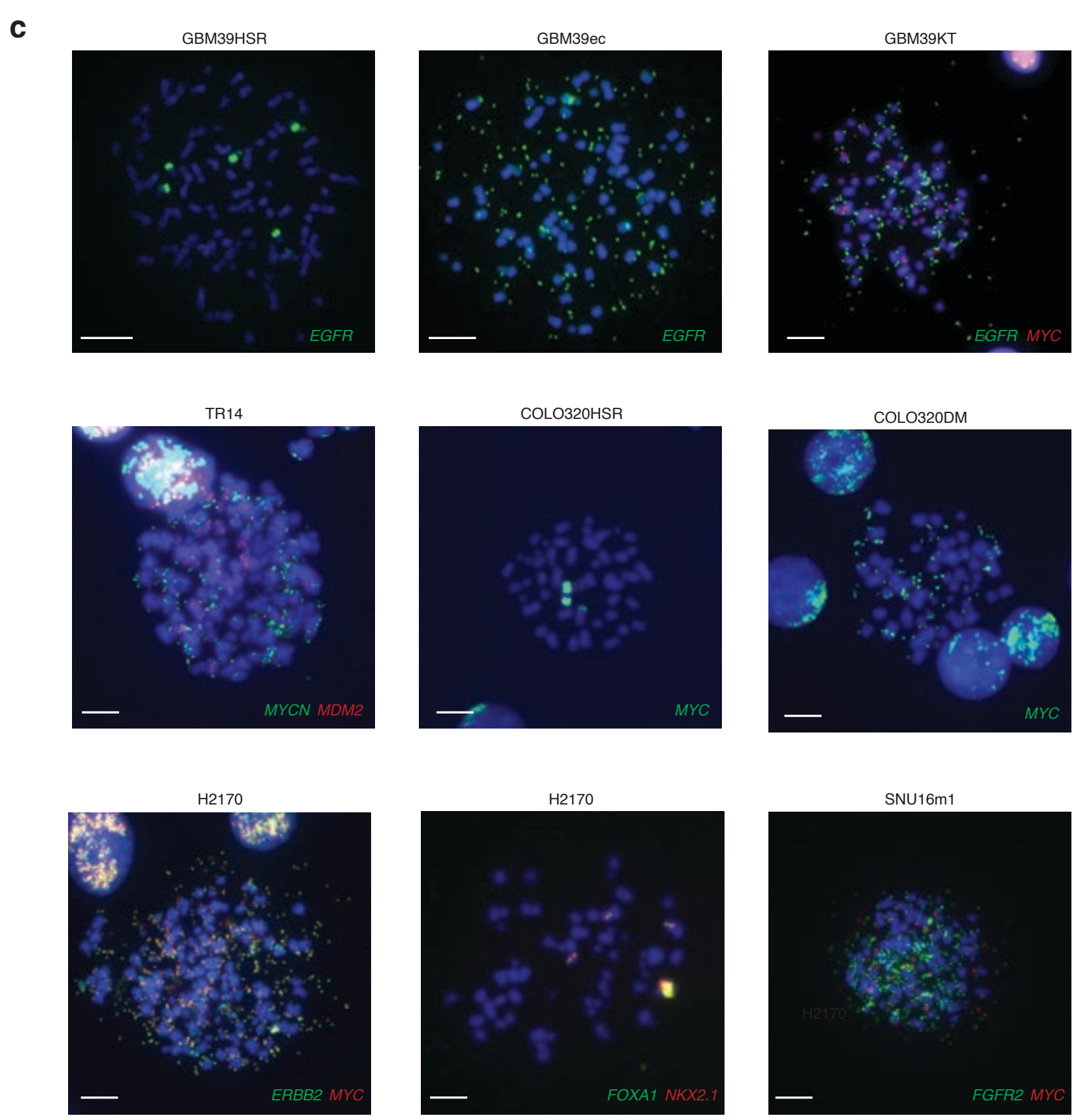

***Revised Extended Data Figure 1c**. (c) Representative images of DNA FISH on metaphase spreads from previous studies demonstrating the amplification mode of key oncogenes used for testing model predictions.*

- Second, we have amended our table summarizing benchmarking results to compare *scAmp* and bulk WGS calls to gold-standard metaphase FISH (revised **Figure 2e**). As discussed above, this analysis underscores that bulk WGS often cannot accurately classify amplifications if they were historically ecDNA (e.g., COLO320-HSR).

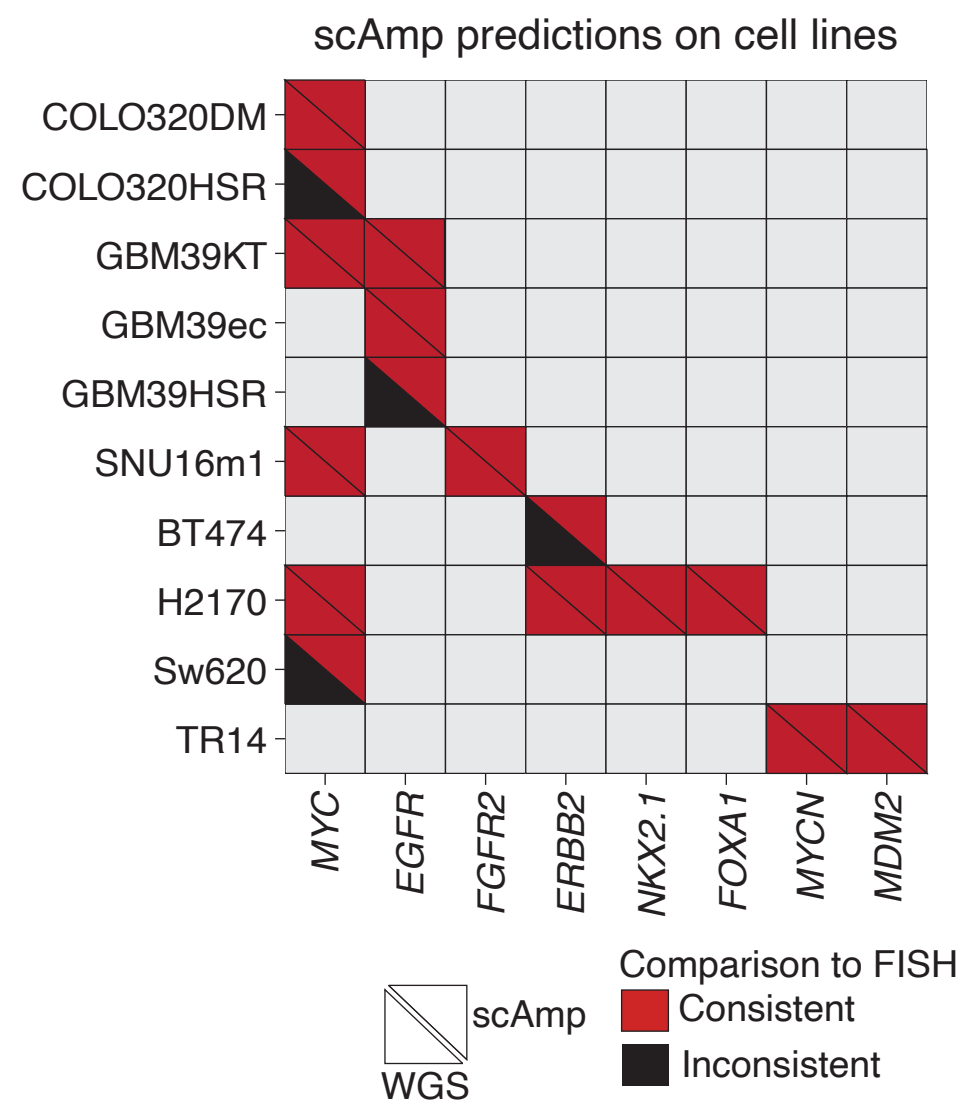

***Revised Figure 2e**. (e) Summary gene-level predictions from scAmp and bulk WGS across cell lines. Agreement between an assay and DNA FISH on metaphase spread ground-truth are marked in red; disagreement is marked in black.*

- Third, we now add single-cell data on cell lines characterized with metaphase DNA FISH lending support for *scAmp*'s effectiveness: the GBM39ec/HSR isogenic pair (revised **Extended Data Figure 1b**) and the colorectal cancer cell line Sw620 (revised **Extended Data Figure 7d-f**). As expected, *scAmp* correctly predicted the *EGFR* amplification status in the GBM39 cell lines, adding further support that *scAmp* can accurately identify chromosomal amplifications. In the Sw620 cell line, bulk WGS predicted an ecDNA amplicon containing *MYC*; however, *scAmp* analysis predicted *MYC* was chromosomally amplified (revised **Figure 2e**). Like the BT474 analysis, we demonstrate with new metaphase FISH data that *MYC* is indeed chromosomally amplified (revised **Extended Data Figure 7f**).

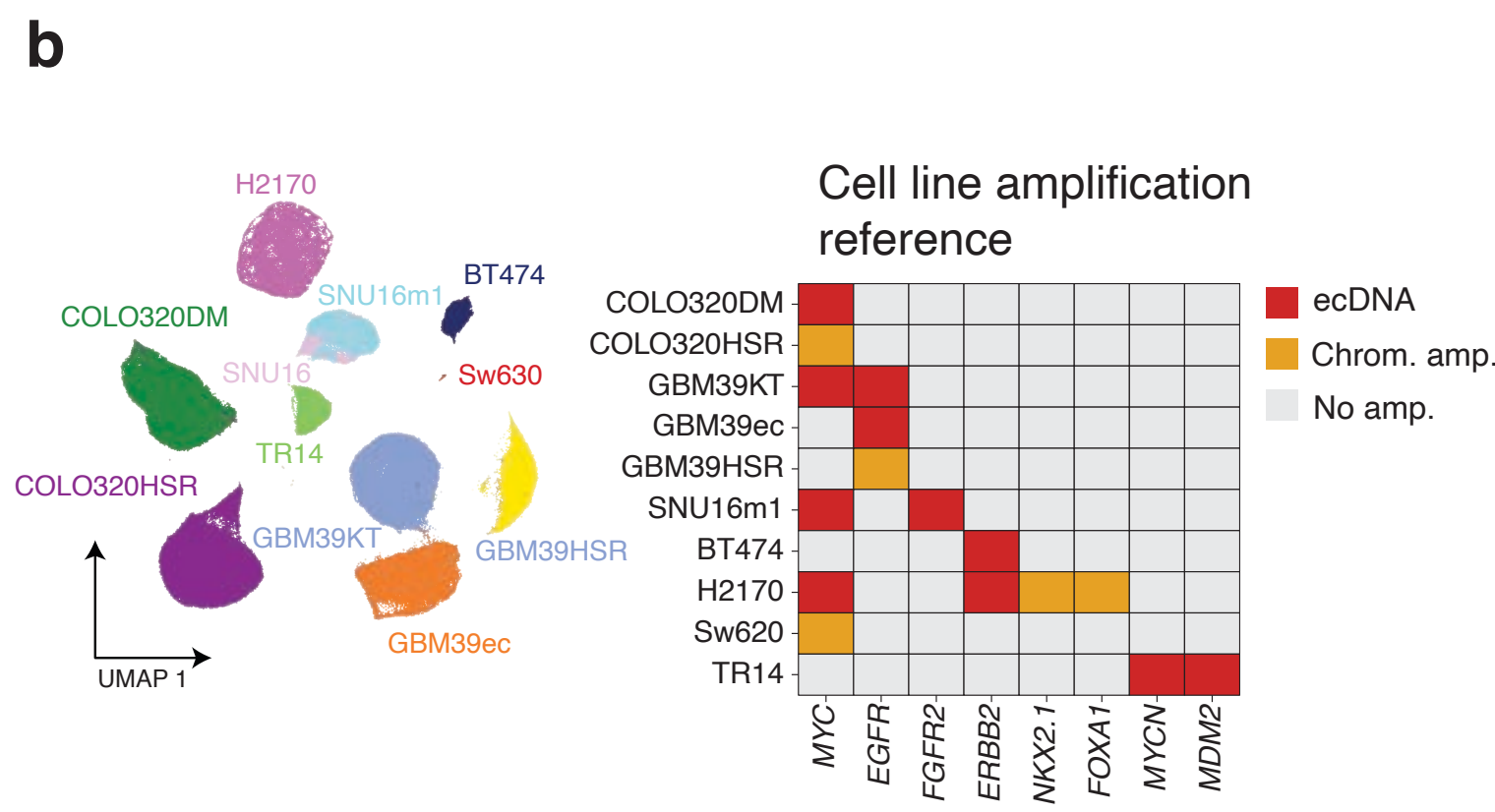

***Revised Extended Data Figure 1b**. A UMAP projection of single-cell ATAC-seq data for cell lines used in this study (left) and a summary of the characterized amplification mode of specific genes (right). Characterization of amplifications is derived from prior reports*

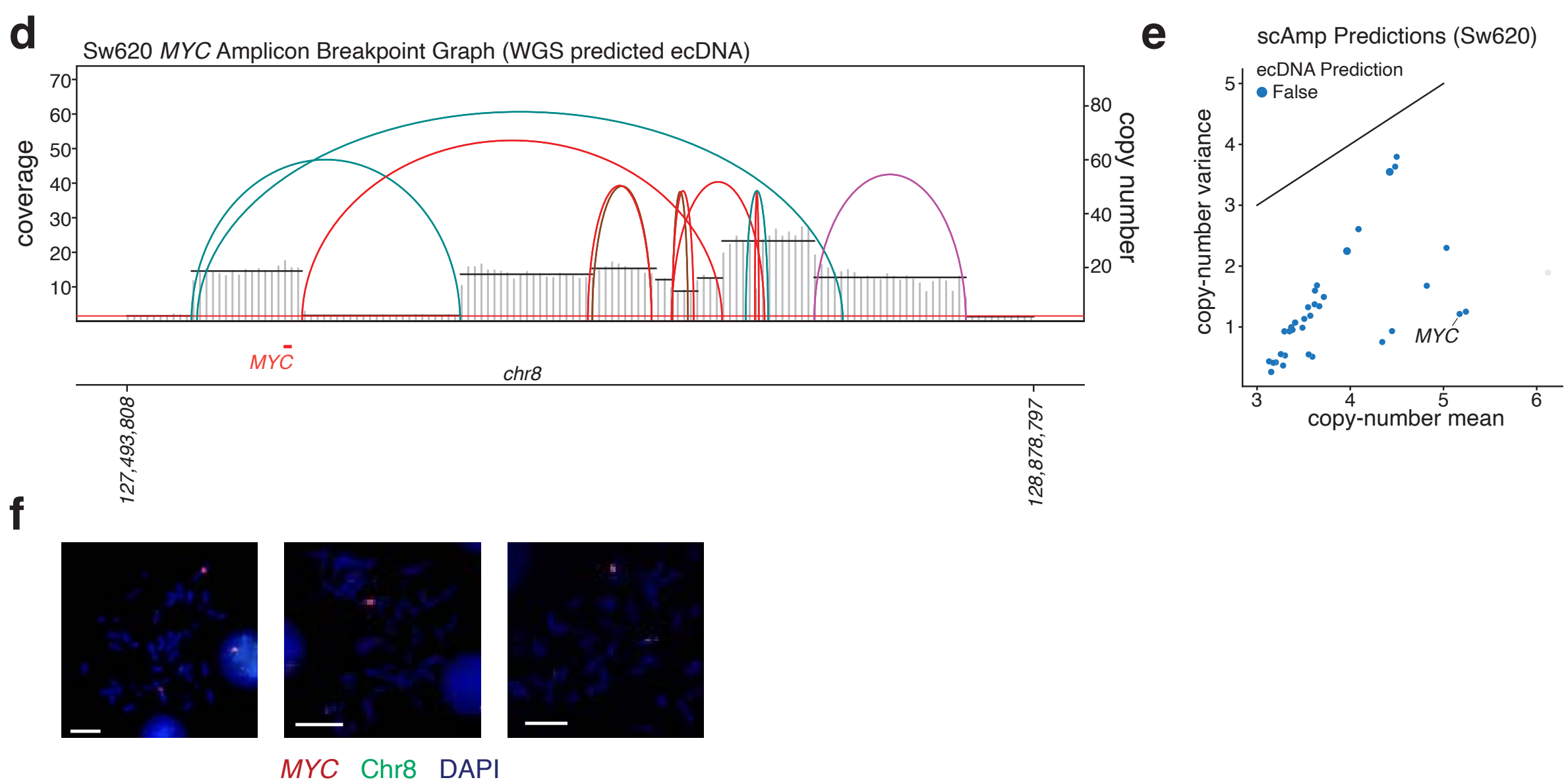

***Revised Extended Data Figure 7d-f.** (d) A breakpoint graph of the MYC amplicon in Sw620 predicted by AmpliconArchitect10 analysis of WGS data as ecDNA+ (obtained from AmpliconRepository). The coverage over the amplified intervals is shown as a grey distribution, and each arc represents a breakpoint inferred from AmpliconArchitect. Only the MYC gene locus is shown underneath for clarity. (e) Scatterplots summarizing the mean and variance of gene copy-number distributions for each gene in the Sw620 cell line, along with predictions from scAmp. MYC is predicted to be chromosomally amplified. (f) Metaphase spread images of cells with DNA FISH for MYC and centromere 8 probe. MYC co-localizes with DAPI+ chromosomal signal and is inferred to be chromosomally integrated in Sw620. Scale bars indicate 10µm.*

2. Single cell resolution language is likely to be misunderstood

What the authors show
scAmp featurizes each gene using distribution statistics computed across cells, then classifies genes as ecDNA associated or chromosomally amplified. The manuscript then defines ecDNA positive cells using thresholds applied to genes predicted to be ecDNA associated.

Why this is a concern
The method does not directly infer ecDNA status per cell. It infers amplification mode at the gene level from across cell distributions, then uses a secondary heuristic to label cells. This is not just a wording issue, since downstream conclusions about subclones and states depend on how cell labels are defined. Readers may interpret “single cell ecDNA calling” as per cell inference unless the two stage nature is explicit.

Revisions suggested

Replace language (including in the title) implying per cell ecDNA calling with more precise phrasing such as “infers ecDNA likelihood for amplified genes using distribution features across cells.”

We agree that this is an important nuance of this study, and we incorporated several suggestions from the reviewer.

Most notably, we have altered the Figure 1 schematic (see below) to reflect the *scAmp* pipeline, and we have also changed the title of the manuscript to “**scAmp enables focal gene amplification analysis from single-cell data**”. We have also rewritten the description of *scAmp* to better reflect this point, as follows (**p. 3**):

> *“scAmp operates on single-cell copy-number data (which can be obtained from assays such as single-cell WGS or single-cell assay for transposase-accessible chromatin by sequencing [scATAC-seq]) and predicts a likelihood for each gene that it is amplified on ecDNA. This principle is similar to algorithms for predicting ecDNA-amplified regions from allelic imbalance in single-cell gene expression data, though the focus on copy-number distributions extends across many platforms. From these predicted ecDNA amplifications, cells can be labeled as ecDNA+ based on their copy-numbers (for example, a threshold based on copy-number heuristics).”*

We have also included this point as a limitation in our Discussion (**p. 9**):

> *“First, scAmp’s workflow relies on predicting ecDNA status of individual genes and then heuristically labeling cells based on copy-number. This may conflate cells that have chromosomal amplifications with those that contain ecDNA, and thus new algorithms that deconvolve these two populations will be useful.”*

Together, we appreciate the reviewer's comments and we believe these changes reflect the utility of this tool: namely that *scAmp* enables the classification of ecDNA+ gene status from single-cell datasets.

Add a schematic or short box describing the two stages: gene level classification, then cell labeling heuristic.

We agree that this is an important clarification and have improved the clarity of the Figure 1 schematic with this suggestion.

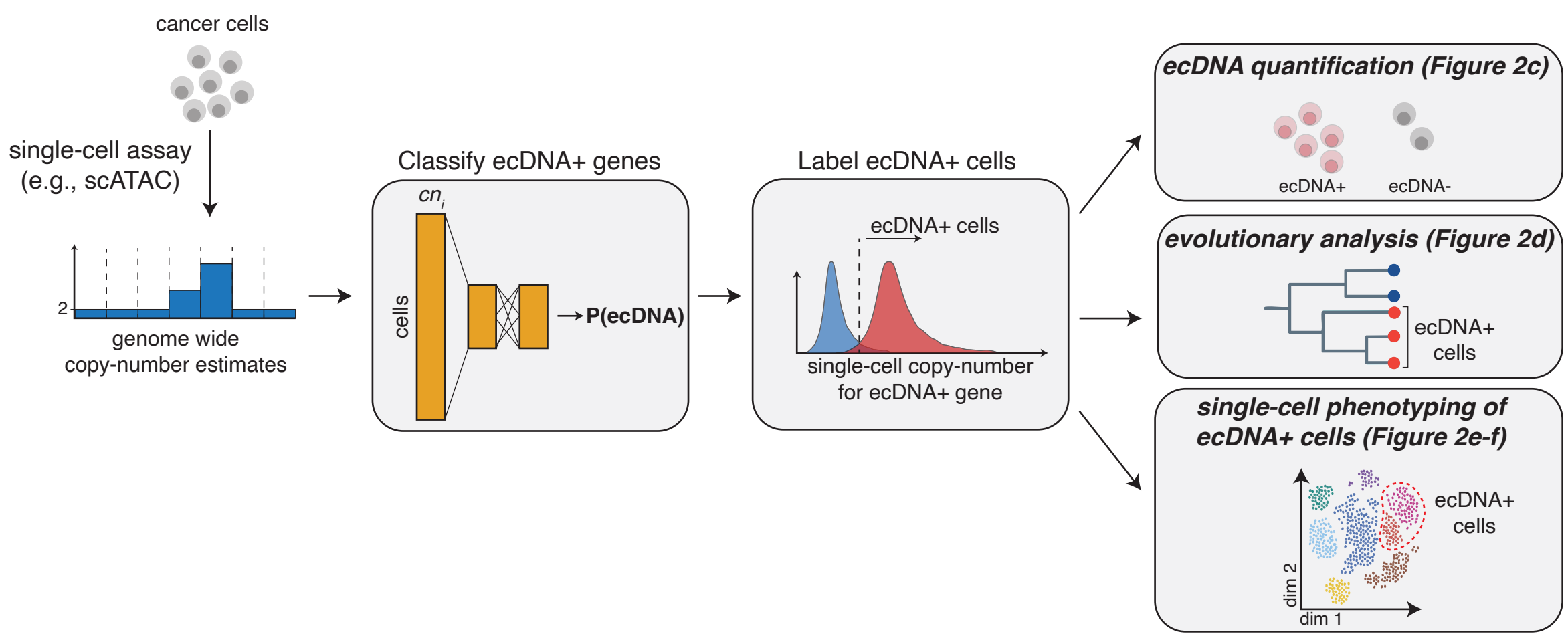

***Revised Figure 1a**. An overview of the scAmp workflow. Copy numbers are inferred from individual cells (e.g., from single-cell ATAC-seq data). For each gene, scAmp then infers the likelihood of that gene being amplified on ecDNA based on the properties of its copy-number distribution across cells. Then, cells are labelled as ecDNA+ or ecDNA- based on a heuristic thresholding scheme. This enables downstream analyses such as quantifying the prevalence of ecDNA in a tumour, assessing an ecDNA's evolutionary history, and investigating the phenotypic effects of ecDNA on cancer cell state.*

Provide sensitivity analysis for the cell labeling heuristic, including varying the copy number threshold and alternative definitions such as requiring multiple ecDNA predicted genes, or weighting by predicted probability rather than hard thresholds.

We thank the reviewer for this suggestion and agree that further characterization of our cell-labeling strategy is warranted. Because we believe that this is an area of future innovation, we have elected to maintain the same cell labeling strategy (while calling this out as a limitation in the discussion; p. 9, see response above) and now provide a sensitivity analysis of the copy-number threshold (revised **Extended Data Figure 9d**).

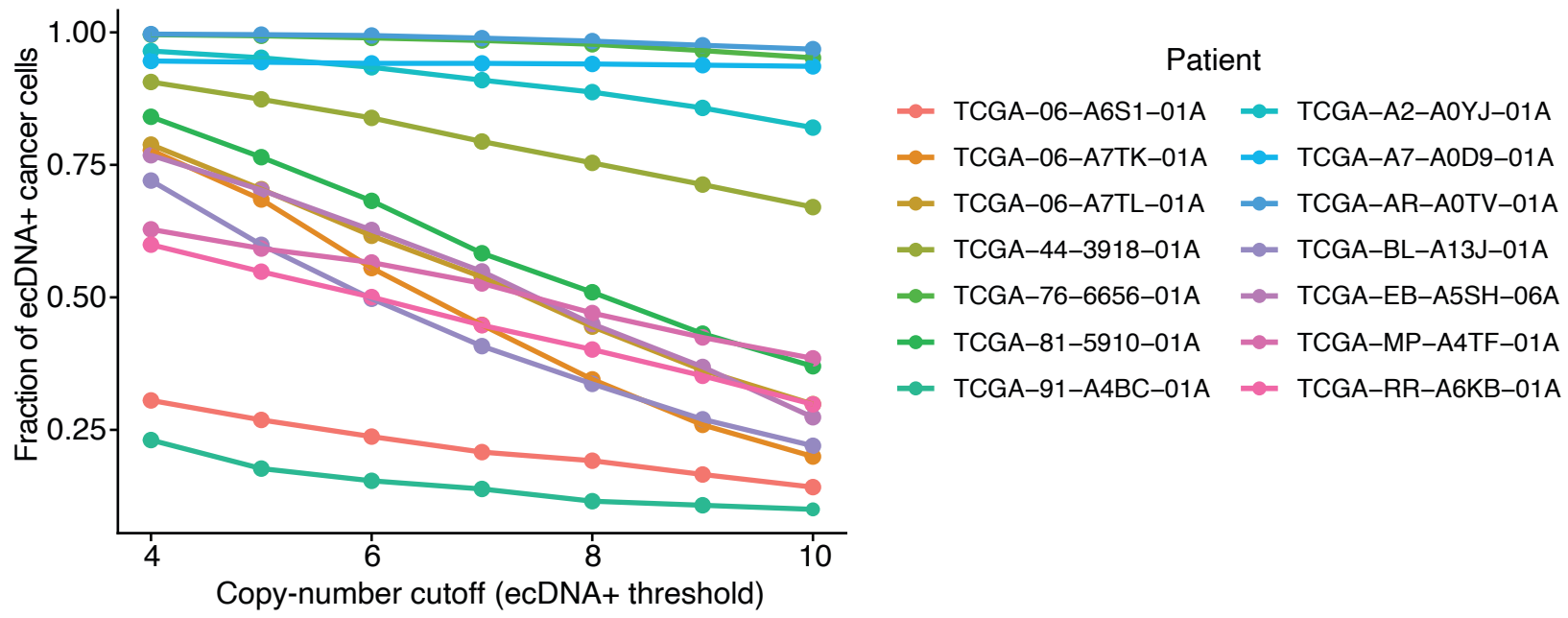

Revised Extended Data Figure 9d*. (d) Fraction of ecDNA+ cancer cells detected in each ecDNA+ tumour with varying copy-number thresholds. Each line indicates a different ecDNA+ tumour.*

Clarify that “single cell resolution” refers to using single cell data to estimate distributions, rather than per cell amplification mode inference.

We agree that this is an important nuance of our current study, and now alter the text in several places to reflect this change; for example in our introduction of *scAmp* on p.3:

> *“scAmp operates on single-cell copy-number data (which can be obtained from assays such as single-cell WGS or single-cell assay for transposase-accessible chromatin by sequencing [scATAC-seq]) and predicts a likelihood for each gene that it is amplified on ecDNA. This principle is similar to algorithms for predicting ecDNA-amplified regions from allelic imbalance in single-cell gene expression data though the focus on copy-number distributions extends across many platforms. From these predicted ecDNA amplifications, cells can be labeled as ecDNA+ based on their copy-numbers (for example, a threshold based on copy-number heuristics).”*

3. Simulation based training requires stronger evidence of robustness to biological variation

What the authors show
Training uses simulated distributions where ecDNA undergoes binomial segregation and chromosomal amplifications are stable, with added technical noise and mixtures of ecDNA amplified, HSR amplified, and non amplified populations.

Why this is a concern
Because scAmp is a distribution based classifier using features like variance, IQR, and quantiles, performance will depend on distribution shape, mixture fraction, copy number magnitude, and tail behavior. Real systems span high burden, low burden, and mixed

states, and some ecDNA associated loci show relatively modest variance. Without explicit evidence that the simulated distributions reflect the diversity of empirical distributions, it is difficult to know how well decision boundaries will generalize beyond settings that align with the simulation assumptions.

Revisions suggested

Demonstrate that simulated ecDNA and chromosomal distributions resemble empirically observed distributions across a panel of ecDNA positive cell lines (6+) spanning high burden, low burden, and mixed (ecDNA/HSR) states. The current cell lines are mainly high burden, which is not representative of all tumors/cell lines (I would think these would be the easiest to predict as well). This could be done by comparing distributions using quantile based metrics or Wasserstein distance with uncertainty, alongside visual overlays.

This is an excellent point and we agree it is important to verify our simulation framework.

To demonstrate that the robustness of our simulations, we now present an analysis comparing the distribution of simulated copy-numbers to validated ecDNA or chromosomal amplifications in cell lines. Our analysis shows that the dispersion of simulated copy-numbers (which we believe carries the largest predictive signal) closely matches that of the desired amplification type (revised **Extended Data Figure 2a**).

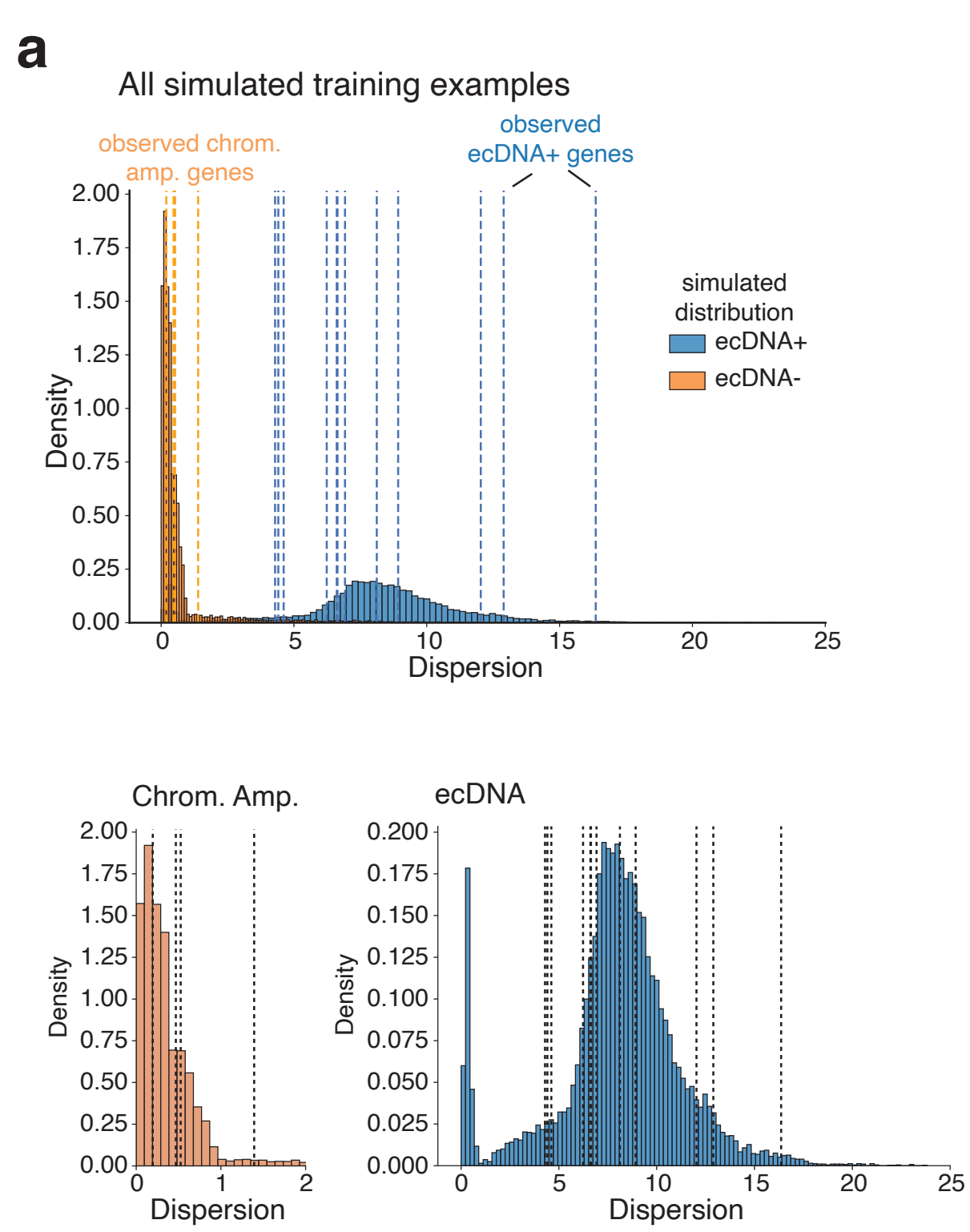

***Revised Extended Data Figure 2a**. Comparison of dispersion values between simulated and observed cell-line copy-number distributions, both the full distribution (top) and for chromosomal or ecDNA amplifications (bottom). In the full distribution, dispersions of observed ecDNA copy-number distributions are indicated in blue; dispersions of observed chromosomal amplification copy-number distributions are indicated in orange.*

Additionally, while our benchmarks on cell lines are not all high-burden (e.g., the mean copy-number of *MYC* in GBM39KT is ~6), these populations are pure. To further demonstrate the robustness of our model, we evaluated performance on *in silico* mixtures of real ecDNA+ and ecDNA- cells (revised **Extended Data Figure 6b**). As expected based off of our simulation setup, we find that our model performs perfectly if at least 20% of cell in a tumor are ecDNA+.

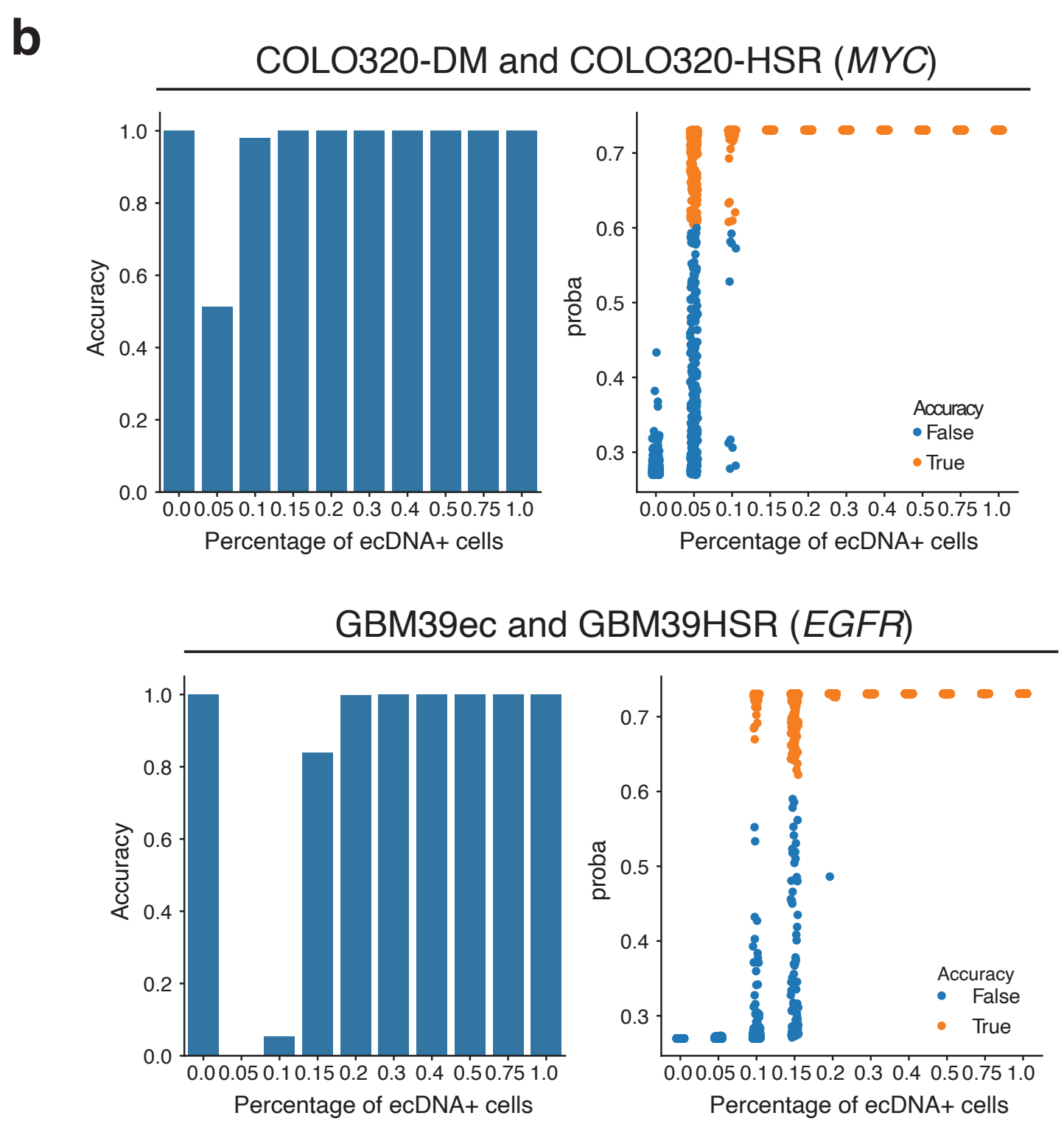

***Revised Extended Data Figure 6b**. Performance of scAmp model on synthetic mixtures of ecDNA+ and HSR+ cell lines (MYC in COLO320-DM/HSR and EGFR GBM39ec/HSR, top and bottom respectively). Model accuracy (left) and probability of amplification (right) are shown for varying amounts of ecDNA+ fraction.*

Thus, because our model performs well on well-characterized cell lines and has high concordance with WGS-based calls in tumors, we believe our simulation framework captures important features of real copy-number distributions.

Include a robustness analysis varying key simulation assumptions such as segregation behavior, technical noise regimes, chromosomal copy number instability, and ecDNA

mixture fraction, reporting AUROC and AUPRC with confidence intervals and model calibration.

We agree that is important to characterize the robustness of our simulation framework. We note that our simulator is built off of previously-published simulators (Hung*, Jones* et al, *Nature* 2024; Lange et al, *Nature Genetics* 2022) and in these previous studies several aspects of the simulation framework were verified, including segregation behavior.

In this study, we extended the simulation to model chromosomal amplification copy number dynamics as well. Based on our observed distributions (e.g., **Figure 1b**) we elected to model chromosomal amplifications as stably inherited. As suggested above, we now include an analysis demonstrating that our simulation recapitulates the variation in copy-number distributions of chromosomally-amplified oncogenes in cell lines (revised **Extended Data Figure 2a** above).

To bolster this analysis, we agree that a natural additional experiment is to test the robustness of our model with varying levels of ecDNA purity. We believe the most compelling evidence of model performance is in the context of real cell lines, and thus we performed experiments mixing together ecDNA+ and ecDNA- cells at varying proportions. We report these results in **Extended Data Figure 6b** and find that *scAmp* has near-perfect performance if at least 20% of cells contain ecDNA (see response above).

Finally, to test the stability of our model with varying levels of noise in the input data, we evaluated performance of *scAmp*, a RandomForest model, and the mean-only null model on predicting ecDNA status of a gene in our simulated data. We incrementally added increasing amounts of Gaussian noise to the feature data and evaluated the AUROC of predictions. The results are presented in revised **Extended Data Figure 6e** and demonstrate that *scAmp* is far more robust than other models tested.

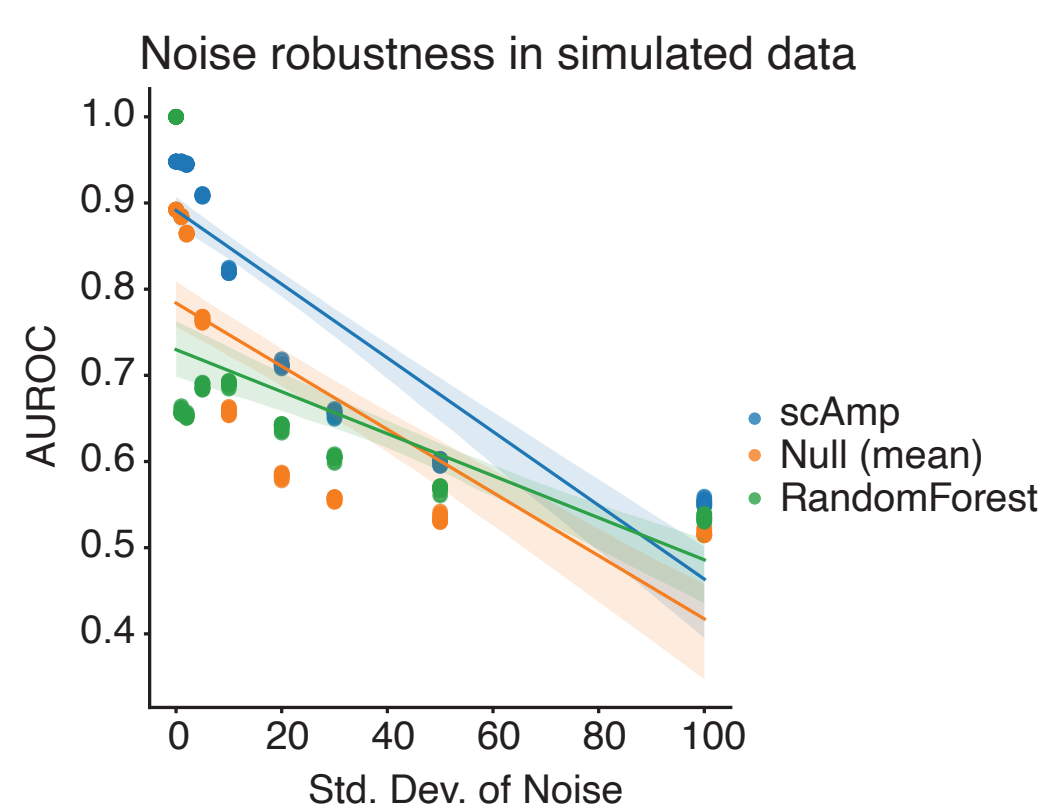

Revised Extended Data Figure 6e. *Performances (AUROC) for scAmp, mean-only null model, and Random Forest model on simulated data with increasing amounts of noise added to input feature matrix (noise modelled by a mean-centred Gaussian distribution with increasing standard deviation).*

Together, we believe these suggestions by the reviewer have markedly improved the trustworthiness of the model training and performance.

Clearly define expected operating regimes and failure modes, such as sparse ecDNA positive subpopulations or low copy ecDNA, so readers understand where the method should be interpreted cautiously.

Thanks to the suggestions from this reviewer, we have further demonstrated *scAmp*'s robustness in a variety of settings. In addition to finding that *scAmp* does well at disambiguating ecDNA status when ecDNA is at low copy-numbers (revised **Extended Data Figure 6c-d**), we now show that our method does well when very few cells contain ecDNA (down to 20% of cells).

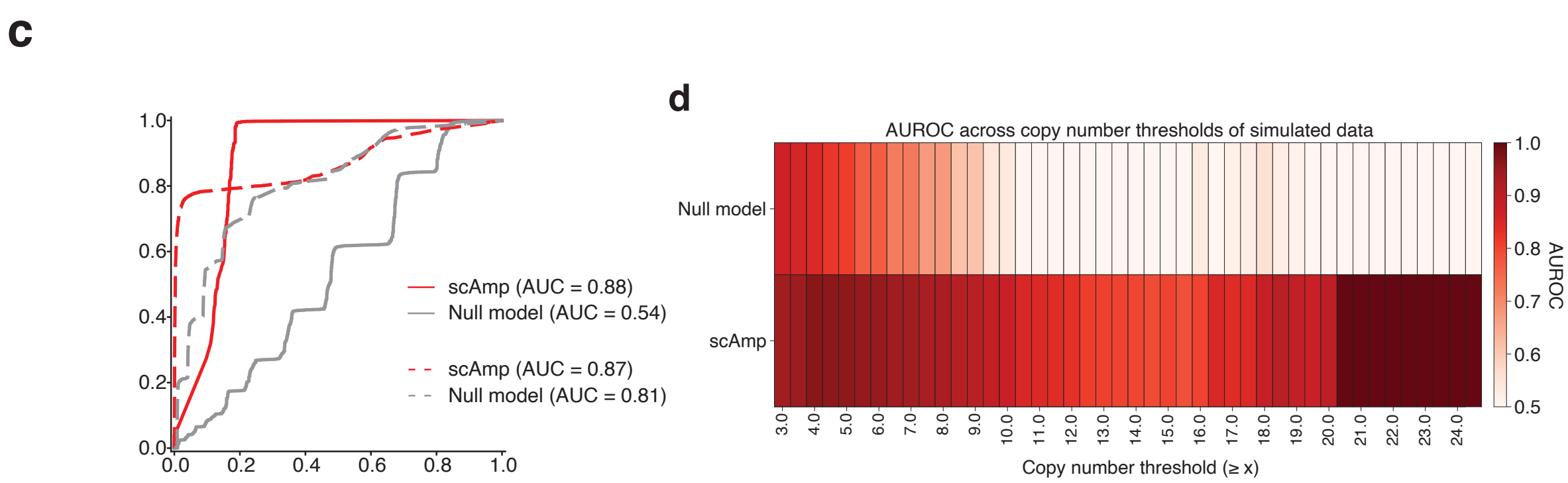

Revised Extended Data Figure 6c-d*. (c) ROC curve of scAmp and mean-only null model on simulated amplicons with average copy-number greater than, or less than, 10. AUROCs are reported in the legend. (d) Summary AUROCs of scAmp and null model on simulated amplicons subset by a minimum copy-number threshold*

Still, we agree that it is important to convey how to best interpret *scAmp*'s results and when it may perform sub-optimally; we have updated our Discussion with these comments (see p.9):

> *"Beyond the potential of scAmp there are important limitations and future directions to acknowledge. First, scAmp's workflow relies on predicting ecDNA status of individual genes and then heuristically labeling cells based on copy-number. This may conflate cells that have chromosomal amplifications with those that contain ecDNA, and thus new algorithms that deconvolve these two populations will be useful. Second, while scATAC-seq has been used widely for copy-number estimation, it is sparse and does not provide as robust copy-number quantification as scWGS approaches that directly measure genetic content."*

4. Validation panel is currently limited in scope for generalizability

What the authors show

Validation focuses on a small number of canonical systems including COLO320 DM versus COLO320 HSR, xenografts derived from these lines, and interphase FISH in FFPE tissue.

Why this is a concern
These experiments demonstrate feasibility and internal consistency, but they do not strongly test generalizability in global settings. COLO320 DM versus HSR is an expected positive control. Xenografts preserve the same underlying genomic architecture. Interphase FISH in FFPE can be suggestive but is not definitive for distinguishing ecDNA from chromosomal or integrated amplification states, especially in mixed or low burden contexts.

Revisions suggested
If the manuscript aims to claim broad discrimination of ecDNA versus chromosomal amplification across tumors, I would encourage a pre-specified validation panel anchored to structure resolving truth. For example:

A blinded set of cell lines (beyond the known and well-studied cases shown here) spanning ecDNA dominant, HSR dominant, and mixed or ambiguous amplification states, including low copy or sparse ecDNA contexts. Structure resolving confirmation by metaphase FISH or an equivalent assay at relevant loci. Application of scAmp to available single cell datasets (or generation of new) for these lines, followed by performance reporting stratified by copy number magnitude and ecDNA fraction.

We thank the reviewer for this comment and we agree that in the case of our study it is critical to present as many validations of our predictions as possible. Our revised manuscript clarifies that for all of the cell lines discussed in the study we have verified the amplification status of oncogenes with a structure resolving approach (i.e., metaphase DNA FISH). These cell lines are not used for training purposes and are only used for testing of models.

As discussed above, we have broadened these analyses in two important ways. First, we have added new cell lines (including three new examples of HSR amplifications: *EGFR* in GBM39HSR, *MYC* in Sw620, and *NXK2.1/FOXA1* in H2170; revised **Extended Data Figure 1c** and **Extended Data Figure 7d-f**). Second, we have demonstrated that *scAmp*'s performance remains high even when mixing together ecDNA+ and ecDNA- cells together (**Extended Data Figure 6b**). We believe that these analyses further underscore the generalizability of these results.

Nevertheless, we concur that that since none of the tumor predictions can be validated, there may be concerns about generalizability and we have thus included this as a limitation in our Discussion:

> *"Third, the benchmarking of scAmp in tumors from the TCGA dataset is limited by the lack of gold-standard validation of ecDNA status (i.e. DNA FISH on metaphase spreads)."*

In the context of the presentation of the interphase FISH results on FFPE, we agree that we demonstrate feasibility but not generalizability. In archival tumors, such as those used in this study, structure-resolving approaches like metaphase FISH are not possible because the samples are fixed. This highlights an important challenge when it comes to detecting ecDNA in patient tissue. We believe *scAmp*'s performance on interphase FISH samples is quite promising as a proof-of-concept test. It was not our intention to claim broad discrimination of ecDNA vs chromosomal amplification across tumors, but rather to demonstrate a proof of concept that the utility of scAmp could extend beyond single cell genomics and we thank the reviewer for the opportunity to clarify our interpretation in the revised manuscript. We have amended the text to provide some additional context for this aspect of our study and to emphasize that we provide proof of concept only and that further study will be needed to establish scAmp's utility in this realm:

> *"Finally, we hypothesized that scAmp's copy-number based approach to assessing ecDNA status could have applications beyond single-cell genomic data. Most archival patient tumors are stored as formalin-fixed paraffin-embedded (FFPE) tissue, which precludes the use of metaphase FISH for determining ecDNA status, as metaphase FISH requires actively cycling cells. Furthermore, although tumor WGS and single cell ATAC-seq are not part of routine clinical practice, DNA FISH on FFPE tissue is routinely used in clinical cytogenetics laboratories for assessing oncogene copy number. Therefore, we asked whether scAmp has the potential to generalize beyond single-cell genomic assays to enable the classification of ecDNA+ tumors from clinically relevant modalities like DNA FISH in FFPE. To establish the utility of scAmp for ecDNA calling in FFPE tissue, we first confirmed that scAmp could accurately distinguish ecDNA amplifications from chromosomal amplifications in FFPE tissue from mouse xenografts of the COLO320DM and COLO320HSR cells (Figure 4a-b; Methods). In order to control for potential segmentation effects, we normalized the MYC signal to centromere 8 signal prior to scAmp analysis. We next extended these results to demonstrate the feasibility of this approach on patient tissues with a proof-of-concept analysis of a tissue microarray (TMA) generated from 14 tumors reported to have focal amplifications in the Stanford Pathology archive, including five tumors reported to amplify MYC (Figure 4c). In the ~1mm tissue cores represented on the TMA, we detected MYC ecDNA amplifications in 2 samples and MYC chromosomal amplification in 1 sample, consistent with the overall appearance of FISH signal in these tumors (Figure 4d-e; Methods). These experiments demonstrate the feasibility of scAmp as a tool for ecDNA detection from DNA FISH, though further study will be needed to establish its generalizability and robustness."*

For FFPE and interphase FISH, frame them as feasibility and explicitly state limitations of predicting ecDNA vs HSR and potential segmentation effects.

We agree that this is an important point and have amended the text accordingly:

> *"In order to control for potential segmentation effects, we normalized the MYC signal to centromere 8 signal prior to scAmp analysis. We next extended these results to demonstrate the feasibility of this approach on patient tissues with a proof-of-concept analysis of a tissue microarray (TMA) generated from 14 tumors reported to have focal amplifications in the Stanford Pathology archive, including five tumors reported to amplify MYC (Figure 4c). In the ~1mm tissue cores represented on the TMA, we detected MYC ecDNA amplifications in 2 samples and MYC chromosomal amplification in 1 sample, consistent with the overall appearance of FISH signal in these tumors (Figure 4d-e; Methods). These experiments demonstrate the feasibility of scAmp as a tool for ecDNA detection from DNA FISH, though further study will be needed to establish its generalizability and robustness."*

We have additionally titled our figure displaying these results as "**Proof-of-concept classification of ecDNA status in FFPE tumours".**

5. scATAC derived copy number estimates need explicit limitation and cross modality checks

What the authors show
A major application is to scATAC seq tumor data, using background signal to infer copy number and variance across cells as a discriminative feature.

Why this is a concern
scATAC seq measures chromatin accessibility, not DNA abundance. Accessibility is influenced by chromatin state and transcription. This is particularly relevant for ecDNA since ecDNA is often highly accessible. This raises the possibility that the method captures a composite of copy number and accessibility effects, which could inflate the variance signal in an assay dependent manner and affect classification and downstream biological interpretations.

Revisions suggested

Add an explicit limitation statement that scATAC derived copy number is an assay dependent proxy and may deviate from DNA based estimates.

We agree that it is important to note that scATAC-seq may produce inaccurate copy-number estimates and have explicitly addressed this limitation in our Discussion:

> *"Second, while scATAC-seq has been used widely for copy-number estimation, it is sparse and does not provide as robust copy-number quantification as scWGS approaches that directly measure genetic content."*

We also note that *scAmp*'s performance is platform-agnostic and can be used in the context of any single-cell copy-number data (including scWGS).

Where paired modalities exist, compare inferred distributions and scAmp outputs between scATAC and DNA based measurements such as scWGS, ResolveOME, or other suitable assays.

We and others have demonstrated previously that copy-numbers from scATAC can recapitulate copy-numbers measured with additional assays.

In our previous work (Hung*, Jones* et al, *Nature* 2024), we used the same scATAC-seq copy-number pipeline to study ecDNA copy numbers. Not only did we demonstrate that scATAC-based quantification could identify elevated copy-numbers of known ecDNA, we also found concordant results between copy-numbers derived from scATAC, scCircle-seq, and metaphase FISH. Moreover, in other previous work from our group (Hung*, Yost* et al, *Nature* 2021), we found that scATAC-based copy-numbers in COLO320DM closely matched the copy-numbers distributions derived from metaphase FISH (see **Reviewer Figure 1**):

[Figure Redacted]

Reviewer Figure 1 (adapted from Hung et al, Nature 2021, Extended Data Figure 5m)*. Cumulative probability of MYC amplicon copy number distributions (mean-centred, scaled) of single-cell ATAC–seq data and DNA FISH data, suggesting that copy number estimates from single cell ATAC-seq data reflect heterogeneity in ecDNA copy number as measured by DNA FISH. P-values determined by Kolmogorov-Smirnov test (1,000 bootstrap simulations).*

Following the reviewer's suggestion, we have additionally compared the copy-number estimates derived from scATAC and bulk WGS in the TCGA tumor samples. We find strong agreement between these samples (Pearson's correlation of 0.54 overall and 0.59 for amplified genes; revised **Extended Data Figure 3c**), though it must be noted that these assays were run on different aliquots and there may be distributional differences between copy-numbers. Together, we believe that these results further add confidence to the scATAC-based analyses presented in this study.

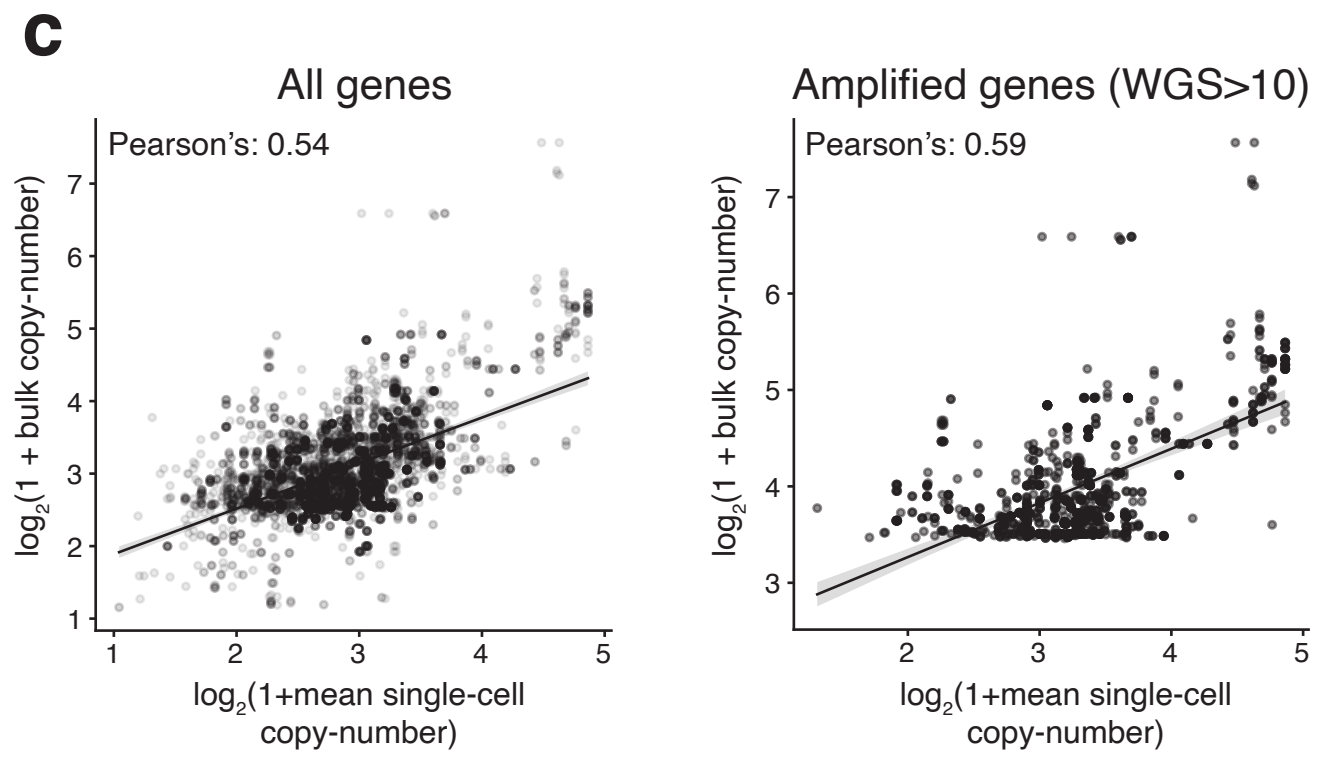

***Revised Extended Data Figure 3c**. Scatter-plot reporting the agreement between gene-level copy-numbers inferred from scATAC-seq and bulk WGS data. Plots and Pearson correlations (ρ) are reported for all genes (left; ρ = 0.54) and just amplified genes (right; ρ = 0.59).*

Include at least one control analysis showing that accessibility alone does not trivially drive ecDNA classification, for example by testing highly accessible non amplified regions or applying normalization approaches and assessing stability.

We agree that this is a critical consideration. We have included this analysis in **Extended Data Figure 1a**, demonstrating that both COLO320HSR and COLO320DM have similar levels of accessibility in the *MYC* locus; however, our model correctly classifies *MYC* amplification status in these two lines (revised **Figure 2e**). We additionally present three new cell lines where *scAmp* accurately identifies chromosomal amplifications (as verified by DNA FISH on metaphase spreads): *MYC* in Sw620, *EGFR* in GBM39HSR, and *NKX2.1* and *FOXA1* in H2170.

a

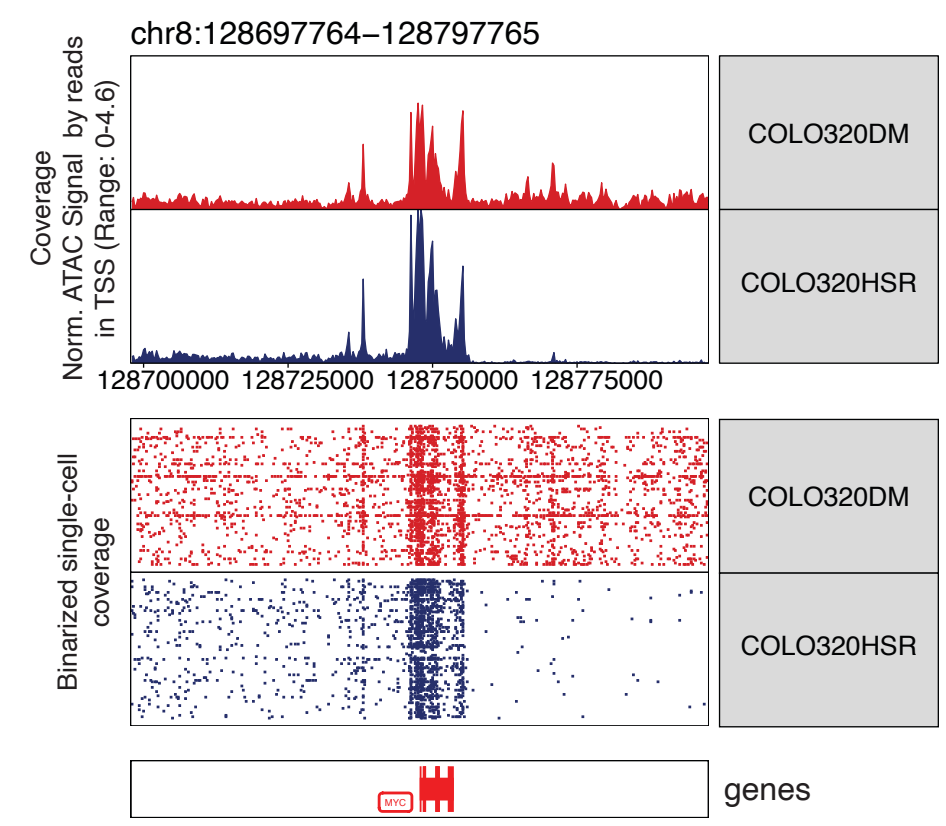

***Revised Extended Data Figure 1a**. Single-cell ATAC-seq coverage for COLO320DM and COLO320HSR cell lines in the MYC locus. Pseubulk coverage (top) and binarized coverage of individual cells (bottom) demonstrate that while the average signal is similar, the COLO320DM ATAC signal appears to have greater variability.*

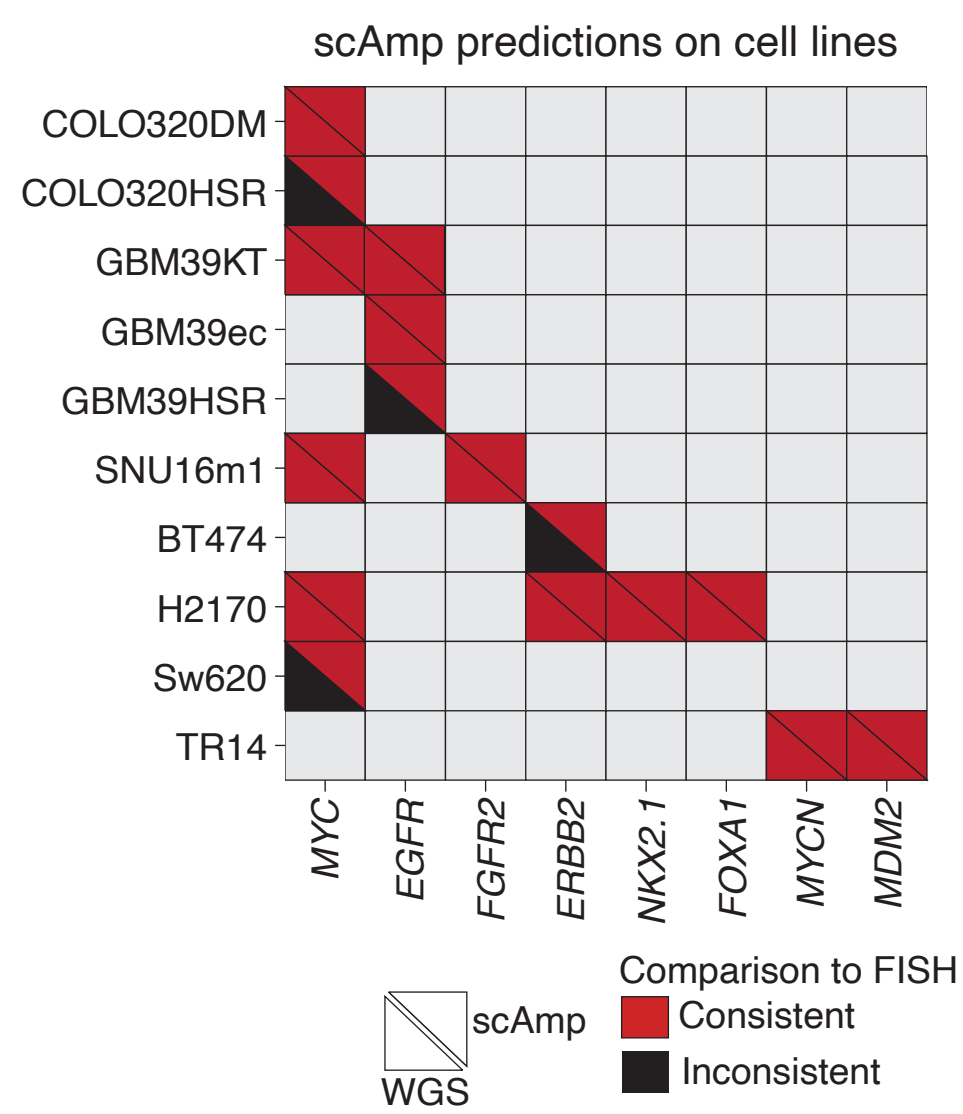

***Revised Figure 2e**. (e) Summary gene-level predictions from scAmp and bulk WGS across cell lines. Agreement between an assay and DNA FISH on metaphase spread ground-truth are marked in red; disagreement is marked in black.*

6. Thresholds and terminology would benefit from sensitivity analyses and standardization

Several thresholds are used for feature extraction, amplification calling, ecDNA likelihood, and cell labeling. These should be justified and sensitivity tested. In addition, clarify what is included under “chromosomal amplification,” for example HSRs, BFB

derived amplicons, integrated ecDNA like structures, and other focal amplification architectures.

We thank the reviewer for these suggestions, and now include several new analyses testing the sensitivity of these parameters:

Revised **Extended Data Figure 6a** reports the effect of several pre-processing choices on *scAmp*'s performance in the cell line data where ground truths are available. We find that *scAmp* remains highly accurate across a variety of pre-processing choices, including the parameter set used in this study.

Revised **Extended Data Figure 9c** reports the fraction of ecDNA+ cancer cells as a function of the minimum copy-number used for ecDNA labeling. As noted above, this is an opportunity for further computational development, though our claims about ecDNA+ fraction variability across tumors remains consistent across a variety of copy-number cutoffs.

Revised **Extended Data Figure 6e** reports the performance of several models (including *scAmp,* a Random Forest model, and our null model) on simulated data that has been adulterated with increasing levels of noise. We find that *scAmp* remains highly accurate, especially compared to other models, across a variety of noise levels.

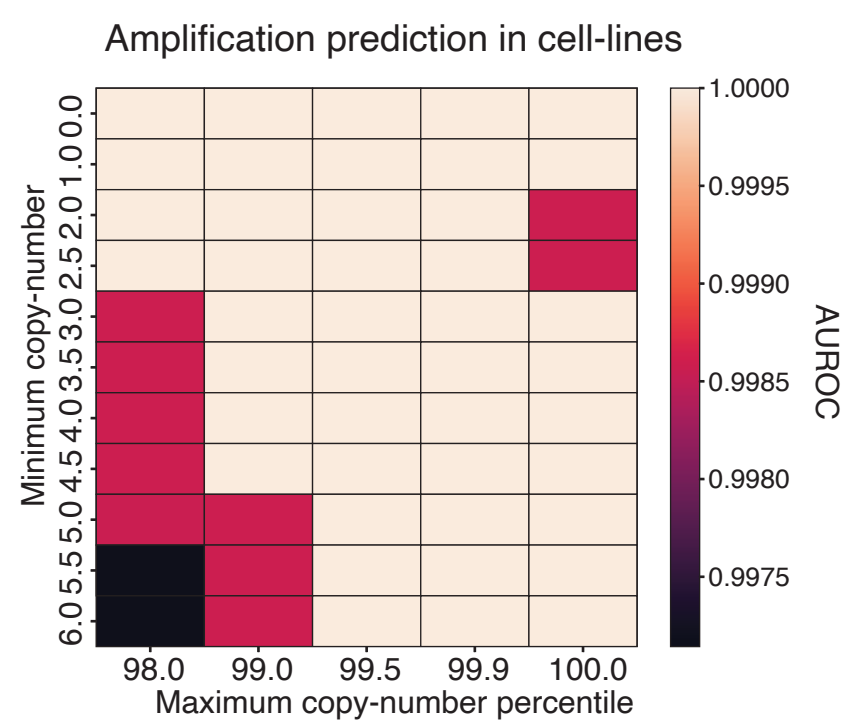

Revised Extended Data Figure 6a. *(a) scAmp model performance, measured by AUROC, on predicting oncogene amplification status in cell lines using various data preprocessing strategies.*

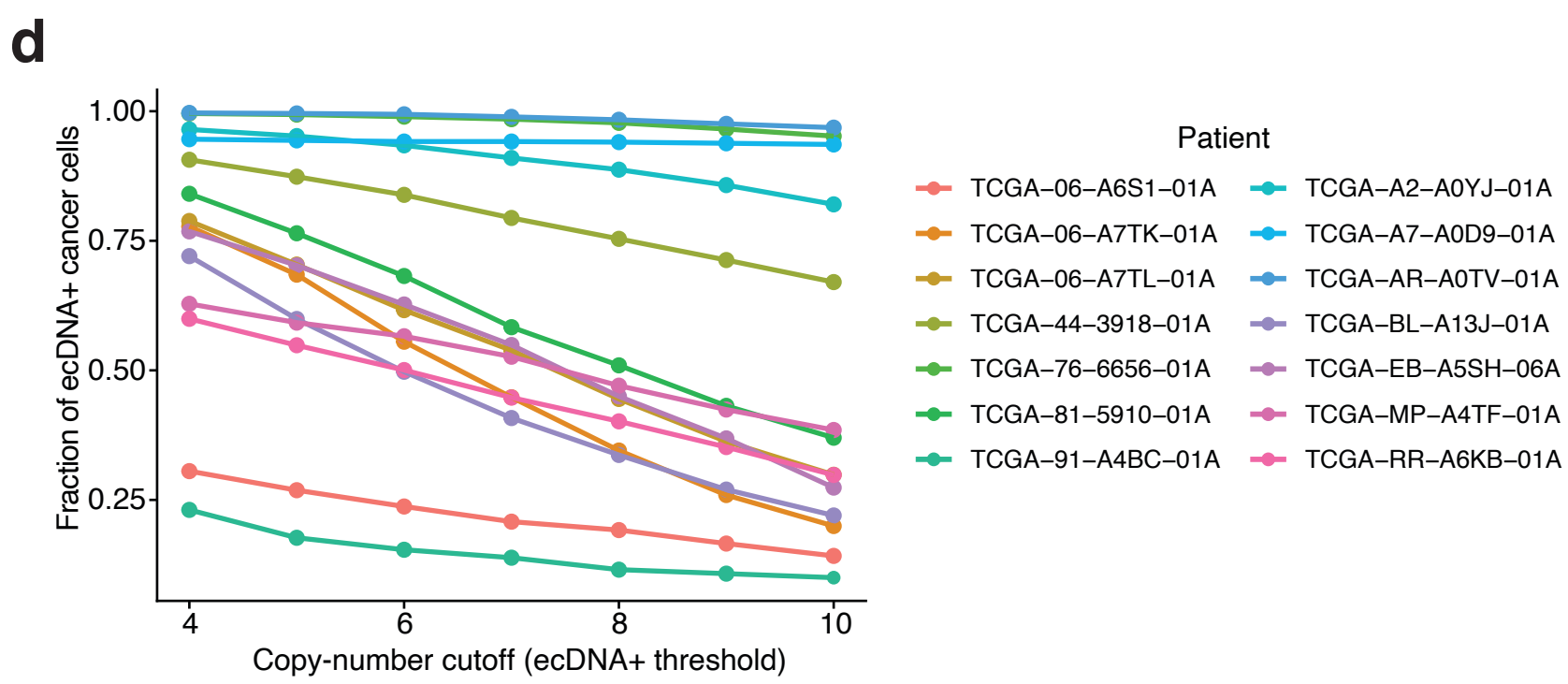

***Revised Extended Data Figure 9d**. (d) Fraction of ecDNA+ cancer cells detected in each ecDNA+ tumour with varying copy-number thresholds. Each line indicates a different ecDNA+ tumour.*

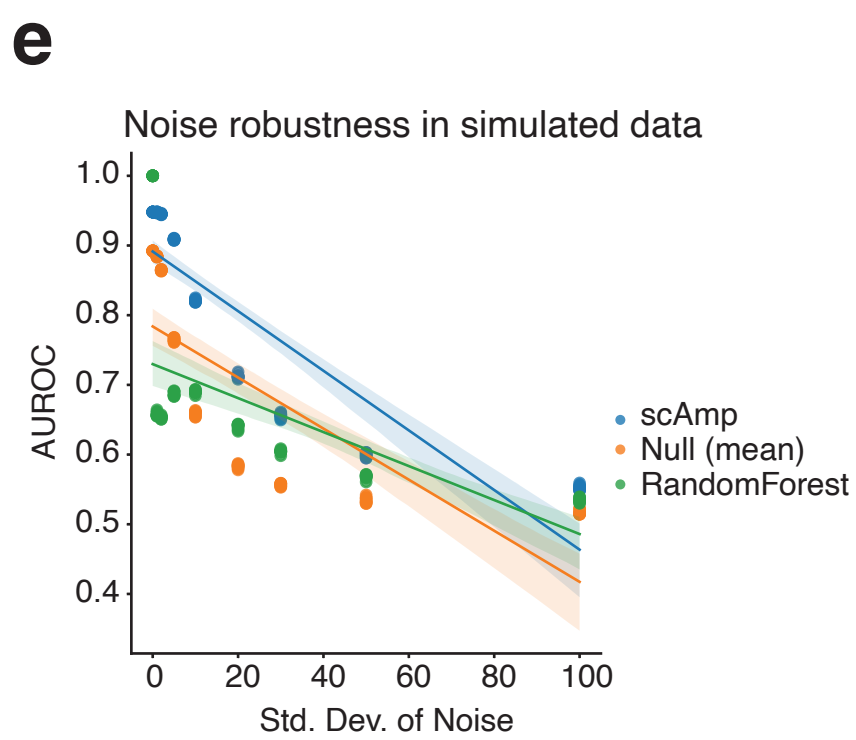

***Revised Extended Data Figure 6e.** Performances (AUROC) for scAmp, mean-only null model, and Random Forest model on simulated data with increasing amounts of noise added to input feature matrix (noise modelled by a mean-centred Gaussian distribution with increasing standard deviation).*

We have also clarified our use of the term "chromosomal amplification" to encompass a variety of linear and complex non-cyclic chromosomal amplifications, including breakage-fusion-bridges (BFBs) and homogeneously staining regions (HSRs), as follows (**p. 2**):

> *"Circular extrachromosomal DNA (ecDNA, or "double minutes") amplifications of are found in approximately 17% of primary tumors overall and are associated with significantly worse patient outcomes and increased drug resistance as compared to chromosomal amplifications (including linear amplifications and complex non-cyclic amplifications like those formed via breakage-fusion-bridge [BFB] processes)."*

Minor revisions

Replace "improved accuracy over WGS" type statements with more precise language

such as concordance with WGS labels, or improved specificity in selected independently validated cases.

We agree and have made these changes throughout the manuscript, see response to Reviewer 3 Comment 1.

Avoid interpreting disagreement with WGS as evidence that WGS is wrong unless orthogonal validation is presented for those cases.

We agree and now diminish these claims unless orthogonal validation is presented (see above, response to Reviewer 3 Comment 1).

Ensure figure captions and Results text clearly separate model inputs, model outputs, and post hoc cell labeling heuristics.

We agree and, as discussed above, have especially amended Figure 1's schematic to reflect this point.

Add explicit caveats where scATAC based copy number estimates are discussed, avoiding phrasing that implies direct DNA copy number measurement without qualification.

We agree and have clarified our interpretation of scATAC-based predictions, while also noting that our method would extend to other single-cell assays (see response to Reviewer 3 Comment 5).

scAmp enables focal gene amplification analysis from single-cell data

Introduction to the Response

We greatly appreciate the time and effort from reviewers and editorial staff for reviewing our manuscript. We are pleased that the reviewers found that our revisions improved the manuscript and that the revised manuscript is suitable for publication in *Nature Communications*.

Please find our responses to all reviewer comments in blue below and highlighted within the main PDF file.

REVIEWERS' COMMENTS

Reviewer #1 (Remarks to the Author):

I thank the authors for their answers and clarifications. I am happy with the revisions and have no further questions. The manuscript introduces a very useful method to identify ecDNA in single cell data.

We thank the reviewer for their comments.

Reviewer #3 (Remarks to the Author):

Review for

scAmp analyzes focal gene amplifications at single cell resolution

The authors have substantially improved the manuscript and addressed the major methodological concerns. The addition of new validation cell lines strengthens the benchmarking framework.

Before publication, I have one remaining concern regarding the provenance and citation of the cell line models used for validation. Because amplification structure (ecDNA versus chromosomal) is central to the claims of the method, each model system should be explicitly anchored to the primary literature that established its structural status (and use as an ecDNA/HSR model). At present, several of the added cell lines are not clearly

linked to their originating studies, which limits transparency, reproducibility and recognition of original work.

To facilitate this, I am asking that the authors explicitly cite the following primary references in a way that corresponds to each model system used in benchmarking in the main text.

COLO320DM / COLO320HSR: https://pubmed.ncbi.nlm.nih.gov/498117/ and Turner et al., Nature 2017.

GBM39 (ecDNA and HSR variants): Nathanson et al., Science 2014.

SNU16 and KATOIII: https://aacrjournals.org/cancerres/article/50/9/2773/496583/Characteristics-of-Cell-Lines-Established-from

Sw620: Turner et al Nature 2017 ?

H2170: https://pubmed.ncbi.nlm.nih.gov/39091515/ and https://www.biorxiv.org/content/10.1101/2025.04.26.650733v1

Including these references, along with a brief sentence in the Methods or Results describing how each model was selected and how its amplification status was determined, would significantly improve clarity and transparency. Without this, it is difficult for the reader to assess how ground truth was defined across the validation panel.

We thank the reviewer for the postive comments and agree that the revised manuscript has improved with the thoughtful input of the reviewers. We thank the reviewer for this suggestion and have modified the text as follows to include the correct references for the cell lines that we used for the validation panel. These cell lines were chosen because they are well-characterized in the field, this rationale is now also included.

> *"We first evaluated model performance on a scATAC-seq dataset consisting of 10 well-characterized cell lines profiled with fluorescence in situ hybridization (FISH) of DNA on metaphase spreads, the gold-standard for assessing amplification status (including COLO320DM and HSR[5,20]; GBM39ec, HSR, and KT[5,14,21]; SNU16m1[14,22,23]; TR14[14,22]; and H2170[15,24,25]; **Supplementary Figure 1b-c).**"*

KATOIII was not a part of our validation panel so is not included in this list. Sw620 is also not part of this validation panel but is used later in the manuscript and is cited at that time:

> *"Similarly, in the colorectal cancer cell line Sw620, we found that scAmp correctly identified MYC as chromosomally amplified (as previously verified with DNA FISH on metaphase spreads[5]"*
